# Dual‐Functional Polystyrene‐Based Borate Complexes: Recyclable Alkyl Radical Sources and Photodegradable Absorbents

**DOI:** 10.1002/chem.71420

**Published:** 2026-07-17

**Authors:** Nami Jimba, Ryosei Ishii, Kazuki Wako, Tomoki Hirohata, Kosuke Sato, Tomohiro Kubo, Kotaro Satoh, Yuto Sumida, Hirohisa Ohmiya, Shinsuke Inagi

**Affiliations:** ^1^ Department of Chemical Science and Engineering School of Materials and Chemical Technology Institute of Science Tokyo Yokohama Japan; ^2^ Department of Chemical Science and Engineering School of Materials and Chemical Technology Institute of Science Tokyo Tokyo Japan; ^3^ Laboratory For Biomaterials and Bioengineering Institute of Integrated Research Institute of Science Tokyo Tokyo Japan; ^4^ Graduate School of Engineering, Kyoto University Kyoto University Katsura Kyoto Japan

**Keywords:** direct excitation, homolysis, organogel, polymer borate complex, radical reaction

## Abstract

Recyclable polymeric materials capable of generating radicals under mild conditions are valuable materials in synthetic chemistry. Herein, we report polystyrene‐based copolymers bearing 2,2′‐(pyridine‐2,6‐diyl)diphenol (PDP) moieties that form alkyl borate complexes and release alkyl radicals upon blue‐light irradiation. The generated primary and secondary alkyl radicals efficiently participate in Giese‐type addition reactions, affording products in good to excellent yields. The polymer‐supported system enables straightforward purification by simple reprecipitation at both the borate complex formation and Giese reaction stages. Notably, the PDP polymer can be recovered through fluoride‐mediated cleavage and regenerated via recomplexation, allowing reuse in subsequent reaction cycles. In addition, crosslinked PDP–borate networks function as photodegradable organogels that selectively absorb organic solvents from water and release them upon light irradiation. This polymer‐supported strategy provides a versatile platform for photo‐driven radical transformations and responsive materials applications, demonstrating proof‐of‐concept for polymer recovery (50% PDP regeneration) and practical advantages in product separation.

## Graphical abstract

Dual‐functional polystyrene‐based polymers bearing 2,2′‐(pyridine‐2,6‐diyl)diphenol (PDP) moieties form photoexcitable alkyl borate complexes: (i) As recyclable radical sources, they enable Giese‐type additions with facile purification by reprecipitation and demonstrate proof‐of‐concept for polymer recovery (50% PDP regeneration). (ii) As photodegradable organogels, crosslinked networks selectively absorb organic solvents and release them upon blue‐light irradiation at ambient temperature.

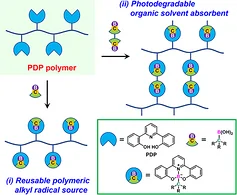

## 1 Introduction

Photochemical generation of carbon‐centered radicals via ultraviolet (UV) irradiation has attracted considerable interest across diverse areas of chemistry, including elucidation of photochemical reaction mechanisms [1, 2] and their use as radical initiators in polymerization [3, 4, 5]. Recent advances in photoredox catalysis have enabled the selective and controlled generation of alkyl radicals from redox‐active precursors for applications in organic synthesis and polymer modification [6, 7, 8]. In such systems, efficient photoexcitation of the catalyst followed by electron transfer under mild conditions is essential. However, the oxidation and reduction steps inherent to the photoredox catalytic cycle can complicate reaction pathways [9]. In this context, direct excitation of a suitably designed radical precursor—thereby bypassing the photocatalyst redox cycle—has emerged as an alternative strategy for generating alkyl radicals under visible‐light irradiation [10]. In this mechanism, energy gained upon photoexcitation induces homolytic bond cleavage, generating carbon‐centered radicals. This approach offers a straightforward and energy‐efficient strategy for visible‐light‐driven organic synthesis. For example, visible‐light excitation of tailored alkyl radical precursors, such as dihydropyridines, dihydropyrimidoquinolinones, and alkyl borate complexes, efficiently produces alkyl radicals for diverse transformations [11, 12, 13, 14, 15, 16, 17, 18, 19, 20, 21, 22, 23, 24, 25, 26]. Among these, 2,2'‐(pyridine‐2,6‐diyl)diphenol (PDP)‐based alkyl borate complexes are quite useful radical sources [21, 22, 23, 24, 25]. However, the separation of products from the resulting boron‐containing byproducts and the recycling of the PDP ligands remain significant challenges. Small‐molecule PDP borate complexes typically require column chromatography for purification, which is time‐ and resource‐intensive.

Herein, we report the development of a polystyrene‐based polymer bearing PDP moieties in the side chains as a platform for the formation of polymeric PDP alkyl borate complexes and their application in light‐driven radical reactions. These PDP polymer–borate complexes offer distinct advantages as versatile functional materials (Figure 1).

- Reusable polymeric radical source: The PDP polymer–borate complexes can be purified by simple reprecipitation during borate formation (a). Upon light irradiation, selective C─B bond cleavage generates carbon‐centered radicals for synthetic transformations, and the products can be readily separated from the polymer by reprecipitation (b,c). The recovered PDP polymer can then be regenerated and reused in subsequent reaction cycles (d).
- Photodegradable organic solvent absorbent: Crosslinked networks derived from PDP polymer–borate complexes absorb organic solvents and release them via light‐induced decrosslinking (e–g).

**FIGURE 1:**
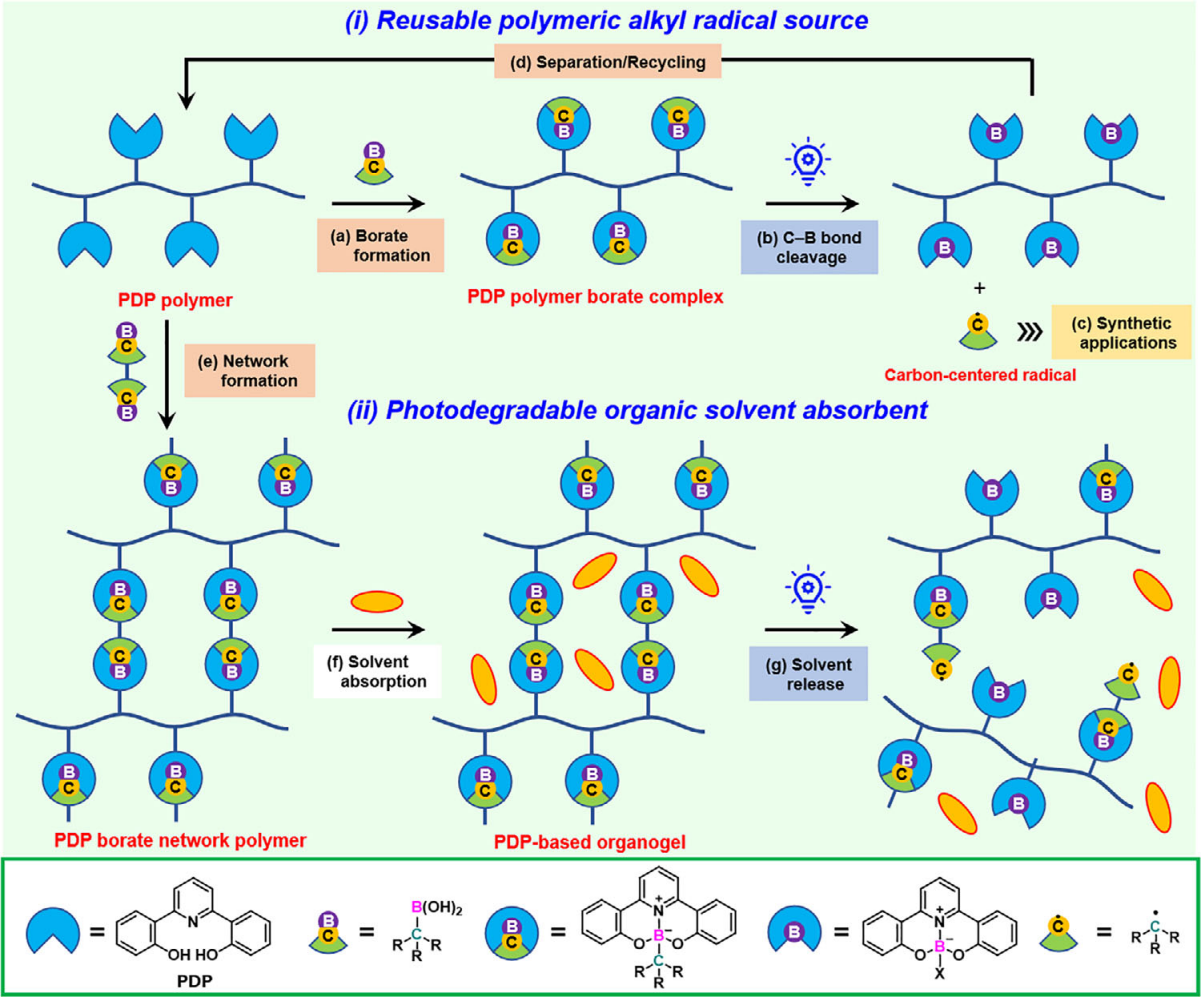
Concept of this study. (i) Reusable polymeric radical *source*: (a) Formation of alkyl borate complexes from the PDP polymer and organoboron species. (b,c) Generation of carbon‐centered radicals upon blue‐light irradiation and their application in organic synthesis. (d) Recovery and regeneration of the PDP polymer for subsequent reaction cycles. (ii) Photodegradable organic solvent absorbent: (e) Formation of crosslinked networks via complexation with bifunctional organoboron species. (f) Application as an organic solvent absorbent. (g) Light‐induced solvent release through photodegradation.

These dual functionalities establish PDP polymer–borate complexes as promising functional materials that combine facile purification with practical applications.

## 2 Results and Discussion

### 2.1 Synthesis of the PDP Polymer and its Borate Complexes

We designed and synthesized a key vinyl monomer bearing a protected PDP moiety. As shown in Scheme S1, 2,6‐bis(2‐methoxyphenyl)‐4‐vinylpyridine (**S1**) was successfully obtained with good yield. Considering the solubility of the polymer in various solvents, we chose to copolymerize **S1** with styrene monomer. Free radical copolymerization of **S1** and styrene (at a 1:9 feed ratio) was carried out in the presence of AIBN as the radical initiator in tetrahydrofuran (THF) at 60°C (Scheme S2). After purification by reprecipitation from methanol, copolymer **P1** was obtained in 93% yield with a number‐average molecular weight (*M*_n_) of 10,700 and a dispersity (*Đ*) of 2.4, as determined by gel permeation chromatography (GPC) relative to polystyrene standards. In the ^1^H nuclear magnetic resonance (NMR) spectrum of **P1**, integration of the aromatic signals relative to the methoxy resonances indicated an **S1**‐to‐styrene incorporation ratio of 1:9, consistent with the monomer feed ratio. Considering that copolymerization of 4‐vinylpyridine and styrene proceeds in a random manner [27], the **S1** units are presumed to be randomly distributed along the polystyrene backbone. Subsequently, **P1** was demethylated by treatment with pyridine hydrochloride (Scheme S3). After purification by reprecipitation, copolymer **P2** bearing the PDP moieties was obtained in 70% yield. Compared to **P1**, **P2** exhibited changes in its GPC parameters (*M*_n_ = 16,500, *Đ* = 4.3), likely due to altered hydrodynamic volume arising from the introduction of polar phenolic groups in the repeating units. Although a high‐molecular‐weight shoulder is apparent in the GPC trace of **P2**, indicating a small degree of crosslinking during the demethylation step, the main distribution remains unimodal, suggesting that the overall polymer backbone structure was largely preserved despite the relatively harsh deprotection conditions.

Borate complex formation between PDP copolymer **P2** and alkylboronic acids was investigated. Initially, 2‐phenylethylboronic acid was employed as a primary alkylboronic acid. **P2** efficiently formed the corresponding borate complex, **P3a**, in 81% yield (*M*_n_ = 13,100, *Đ* = 3.9). In the ^1^H NMR spectrum of **P3a**, new signals appeared at approximately 0.6 and 2.5 ppm, assignable to the ethylene protons of the borate moiety (Figure 2). The degree of borate formation was estimated to be ∼80% based on the integration ratio of the signal at 0.6 ppm relative to the aromatic signals. Next, 1‐*N*‐Boc‐pyrrolidin‐2‐ylboronic acid was used as a secondary alkylboronic acid, affording polymer complex **P3b** in 85% yield (*M*_n_ = 17,100, *Đ* = 5.0). In the ^1^H NMR spectrum of **P3b**, characteristic signals at approximately 3.0 ppm, attributed to the pyrrolidine moiety of the borate, were observed. The degree of alkylation was estimated to be ∼65%. In both primary and secondary alkyl borate formations, relatively high degrees of alkylation were achieved. Notably, the polymer–borate complexes were readily purified by reprecipitation from poor solvents such as methanol, whereas the corresponding small‐molecule borate complexes typically require column chromatography, which is more time‐ and resource‐intensive [21].

**FIGURE 2:**
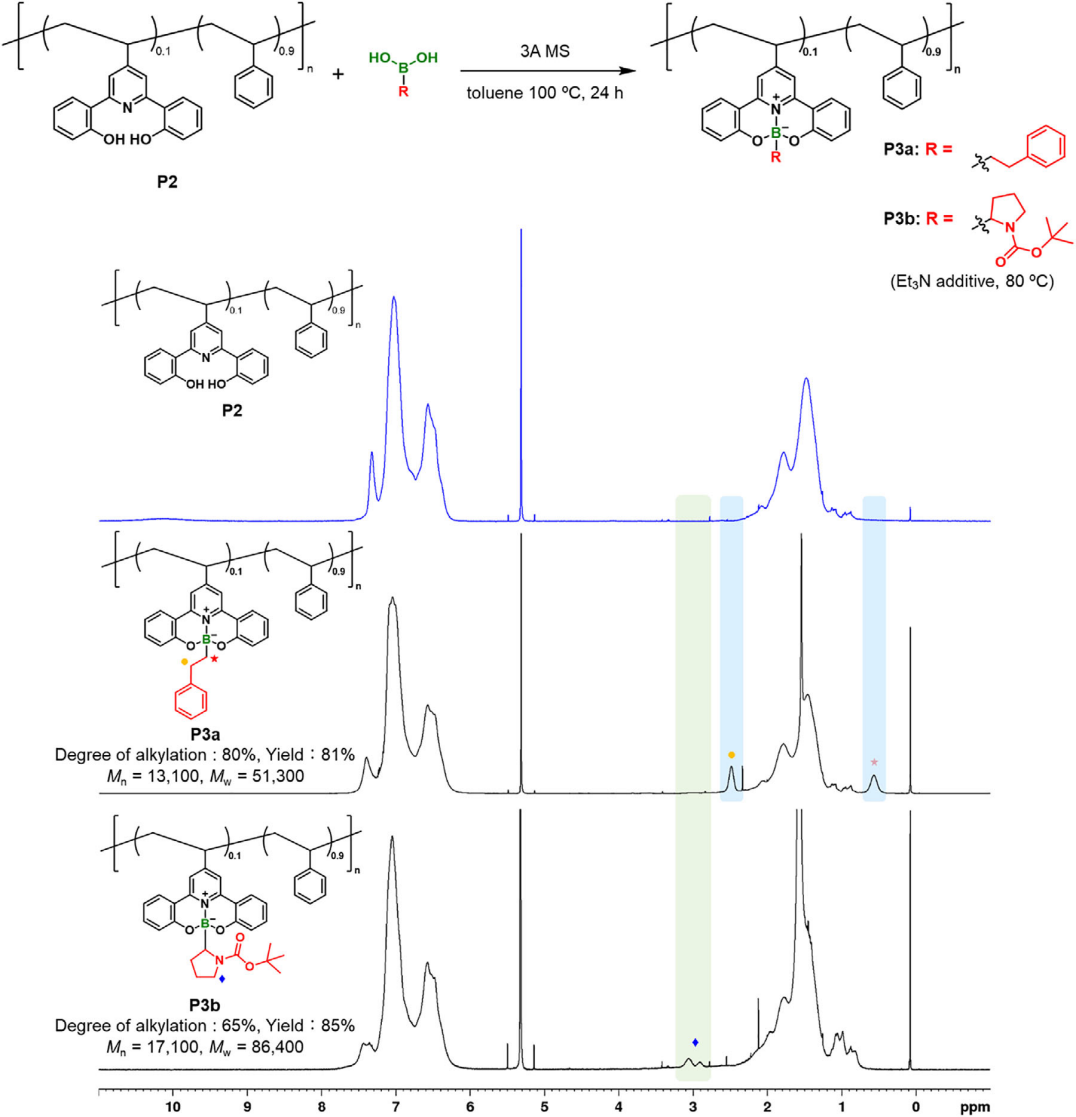
(a) Schematic representation of borate complex formation between **P2** and alkylboronic acids. (b) ^1^H NMR spectra (500.13 MHz, CD_2_Cl_2_) of **P2**, **P3a**, and **P3b**.

### 2.2 Use of PDP Polymer–Borate Complexes as Alkyl Radical Sources for Organic Reactions

To investigate the optical properties of the PDP polymers upon borate complex formation, UV–visible absorption spectroscopy was performed in solution (Figure S2). PDP polymer **P2** exhibited an absorption maximum at 320 nm. After borate complexation, both **P3a** and **P3b** displayed two absorption bands at approximately 330 and 370 nm, with an absorption edge extending to 420 nm. These results indicate that the polymer–borate complexes absorb in the blue‐light region and are therefore suitable for direct photoexcitation.

Following direct photoexcitation of small‐molecule PDP–borate complexes to generate alkyl radicals, the photoirradiation of PDP polymer–borate complexes in solution was examined. Alkyl radical generation was first evaluated in the presence of the persistent radical 2,2,6,6‐tetramethylpiperidine 1‐oxyl (TEMPO). A solution of **P3a** (2 mL) containing TEMPO (2 equiv per PDP–borate moiety) was irradiated with a blue LED (Figure S1) at room temperature for 1 h in various solvents (Table S1). In the ^1^H NMR spectrum of the crude product obtained under the conditions in entry 3, the broad signals at 0.6 and 2.5 ppm disappeared completely, and new sharp signals emerged at 2.9 and 4.0 ppm (Figure S3). These changes indicate quantitative C─B bond cleavage to afford the phenylethyl–TEMPO adduct (**1a**) in 89% NMR yield. In contrast, polar solvents such as acetonitrile and methanol—commonly used in photochemical reactions—were unsuitable because **P3a** is not soluble. 1,2‐Dichloroethane (DCE) provided the highest yield and was therefore selected for subsequent experiments. Under these conditions, photochemical C─B bond cleavage of **P3b** also proceeded to completion, as confirmed by the ^1^H NMR spectrum (Figure S4). The broad signal at approximately 3.0 ppm disappeared, and new sharp signals appeared at 3.4 and 5.7 ppm, consistent with formation of the pyrrolidinyl–TEMPO adduct (**1b**, 65% NMR yield). These results demonstrate that PDP polymer–borate complexes (**P3a** and **P3b**) are readily photoexcited to generate the corresponding primary and secondary alkyl radicals, respectively.

To gain insight into the mechanism of photoinduced C─B bond cleavage, TD‐DFT calculations were performed on a small‐molecule PDP–borate model complex at the CAM‐B3LYP/ 6–31G+(d,p) level of theory (Figure S5). Relaxed potential energy surface scans along the C─B bond coordinate in the S1 excited state revealed that elongation of the C─B bond is accompanied by planarization of the PDP moiety, leading to a stabilized intermediate. The estimated Gibbs free energy for the radical‐releasing state (C─B = 4.0 Å) was +18.9 kcal mol^−1^ relative to the S1 minimum, consistent with efficient radical generation at room temperature. These calculations support the proposed mechanism of homolytic C─B bond cleavage upon direct photoexcitation of the PDP–borate complex.

Based on previous reports describing small‐molecule PDP–borate complexes, Giese‐type addition reactions of photo‐generated alkyl radicals derived from PDP polymer–borate complexes were investigated. A solution of **P3a** containing dimethyl fumarate (**2A**) as the radical acceptor was irradiated with blue light at room temperature for 14 h (Table S2). In entry 1, using DCE as the solvent afforded the desired phenylethyl radical adduct (**3aA**) in 30% NMR yield. However, gelation of the reaction mixture was observed, likely due to network formation involving the borate‐derived radical anion (Figure S6), which reduced reaction efficiency. The addition of a small amount of methanol (entry 2) suppressed gelation and improved the yield of **3aA** to 43%. Previous studies on small‐molecule systems [25] have shown that borate concentration is critical in Giese‐type reactions, with lower concentrations significantly enhancing product yields. Consistent with this trend, decreasing the concentration of the polymer–borate complex slightly increased the yield of **3aA** to 46% (entry 3), although the effect was modest. This limited improvement may reflect the fact that the local concentration of borate moieties within the polymer is dictated by the copolymer composition (PDP:styrene = 1:9) and therefore remains constant irrespective of the overall solution concentration. Figure S7 shows the ^1^H NMR spectra of the crude product obtained in entry 3 of Table S2 and that of **P3a**. In addition to the signals corresponding to adduct **3aA**, a new methoxy signal appeared at 3.2 ppm [21]. This signal provides evidence for the formation of a methoxy borate species within the polymer (**P5**) following photochemical alkyl radical release.

With the optimal conditions established, Giese‐type reactions of **P3a** with several radical acceptors (**2A–2C**) were investigated (Figure 3). When **2B** was employed as the radical acceptor, adduct **3aB** was obtained in a modest 24% yield. In contrast, acceptor **2C** afforded adduct **3aC** in 84% yield. The combination of a highly reactive primary alkyl radical (phenylethyl radical) and electron‐deficient radical acceptors generally provided moderate to high yields. Next, **P3b**, a precursor of a secondary alkyl radical (pyrrolidinyl radical), was examined in reactions with the same radical acceptors (Figure 3). Under these conditions, adducts **3bA** and **3bB** were obtained in excellent yields (94% and quantitative yield, respectively). In contrast, the reaction of the pyrrolidinyl radical with the internal alkene **2C** afforded **3bC** in low yield, likely due to steric hindrance. As a representative example, the ^1^H NMR spectra of **P3b** before and after reaction with **2A** are shown in Figure S8. The yield variations observed across the substrate scope are consistent with the electronic and steric properties of the radical acceptors, as commonly reported for Giese‐type radical additions.

**FIGURE 3:**
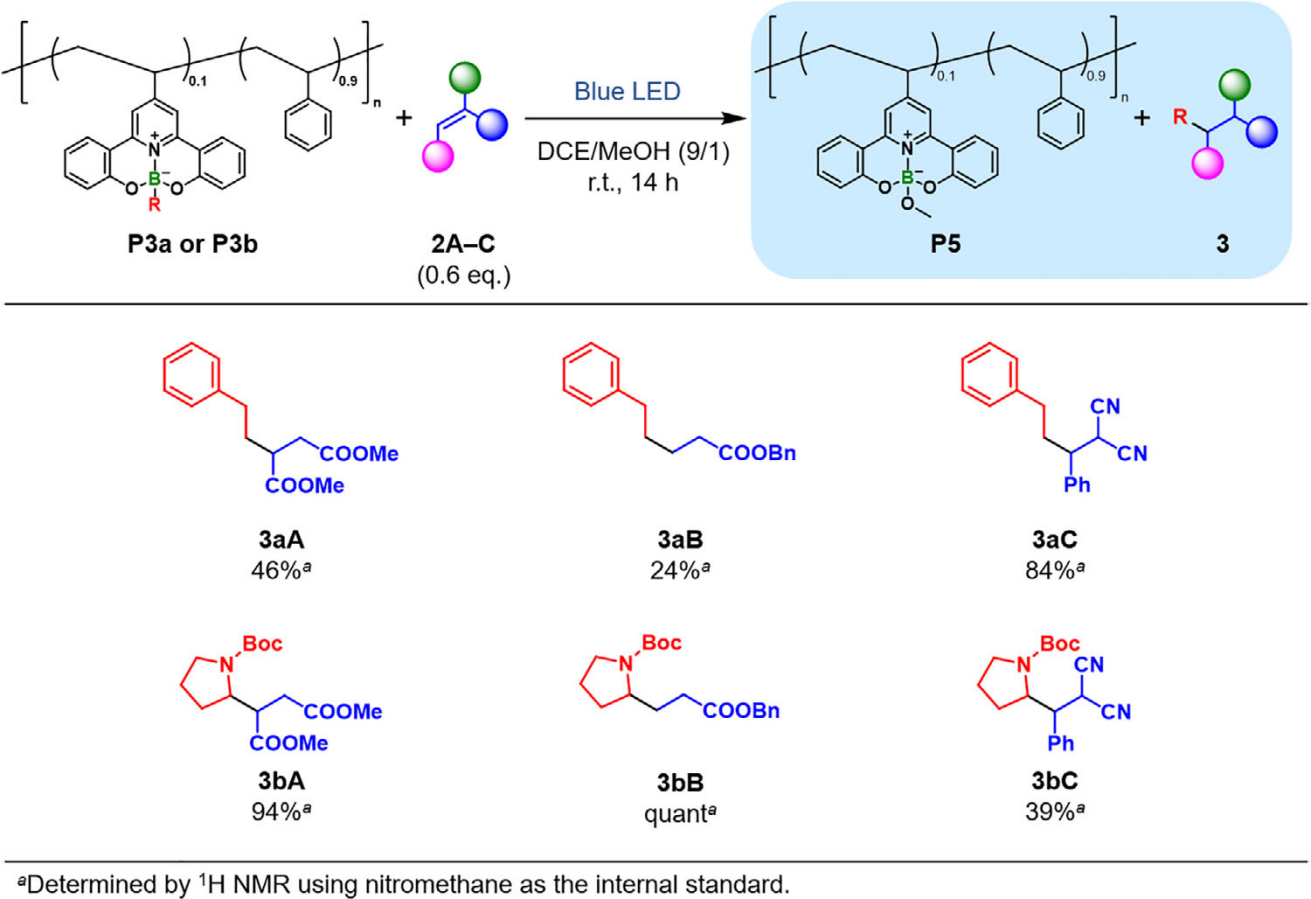
Substrate scope of Giese‐type addition reactions using **P3a** and **P3b**.

These results demonstrate that PDP polymer–borate complexes serve as effective precursors of alkyl radicals for organic synthesis.

### 2.3 Recycle Use of PDP Polymer

As described above, the PDP polymer bearing a methoxy borate moiety (**P5**) was obtained as a residue of the Giese‐type reactions. This stable polymeric borate complex was readily isolated by reprecipitation using chloroform as a good solvent and methanol as a poor solvent. The regeneration of the PDP polymer was then examined (Table S3). **P5** was converted back to the PDP polymer (**Re‐P2**) upon treatment with tetrabutylammonium fluoride (TBAF). The fluoride ions from TBAF were activated in diglyme [28]. The ^1^H NMR spectrum of **Re‐P2** showed pronounced changes in the aromatic region and in the methoxy signal compared with that of **P5** (Figure S9a). In addition, a substantial decrease in the characteristic signal was observed in the ^11^B NMR spectrum of **Re‐P2** (Figure S9b). These results indicate that the PDP moieties in the polymer were largely regenerated. To quantify the recovery of PDP units, the phenolic groups in **Re‐P2** were converted to the corresponding triflates. As a control experiment, triflation of **P2** was first examined, and quantitative conversion to **P2OTf** was confirmed by ^19^F NMR analysis using an internal standard (Scheme S7). Accordingly, triflation of **Re‐P2** enabled estimation of the recovery efficiency of PDP moieties, revealing that approximately 50% of the PDP units were regenerated under the optimal conditions (entry 4, Table S3). While the recovery efficiency requires further optimization, this demonstrates the proof‐of‐concept for polymer regeneration via fluoride‐mediated cleavage. The polymer‐supported approach offers the practical advantage of facile purification by simple reprecipitation, compared to the column chromatography required for small‐molecule analogues. The incomplete recovery likely reflects both the inherent reactivity of the present PDP moiety toward fluoride‐mediated cleavage and diffusion limitations within the polymer matrix. Further optimization, including the design of alternative polymer architectures and reaction conditions, remains a subject for future investigation.

Recomplexation of **Re‐P2** with phenylethylboronic acid was subsequently examined to regenerate the PDP polymer–borate complex (**Re‐P3a**) (Scheme S8). The alkyl borate was successfully re‐formed with a high degree of alkylation (∼80% relative to the recovered PDP moieties in **Re‐P2**), as determined by ^1^H NMR analysis (Figure S10). Based on the overall PDP content of the original polymer, this corresponds to approximately 34% recomplexation efficiency (Figure 4). The GPC profiles of the polymers obtained at each stage are summarized in Figure S11. Although slight variations in the profiles were observed during borate complexation, PDP recovery, and recomplexation, unimodal distributions were maintained throughout the entire process. These results indicate that neither gelation nor significant backbone degradation occurred during the photochemical reaction or the fluoride treatment steps.

**FIGURE 4:**
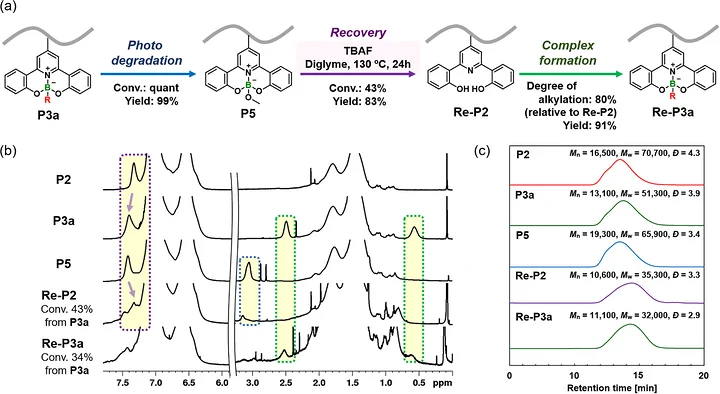
(a) Conversion and yields for the sequential reaction steps, including recomplexation. (b) ^1^H NMR spectra and (c) GPC traces of the polymers obtained at each stage.

Figure 4 summarizes the quantitative data for the sequential recovery processes, including photochemical C─B bond cleavage (**P3a** → **P5**), regeneration of the PDP polymer via TBAF treatment (**P5** → **Re‐P2**), and recomplexation (**Re‐P2** → **Re‐P3a**). Each polymer intermediate was readily isolated and purified by simple reprecipitation with high collection efficiency, owing to its polymer‐supported architecture. This straightforward recovery protocol for both the PDP polymer and its borate complex, enabled by the polymer‐supported architecture, represents a promising strategy for developing recyclable alkyl radical sources.

### 2.4 Network Polymer Formation and Its Application to an Absorbent Material

Based on the efficient borate formation of PDP polymer **P2** and the selective C─B bond cleavage of **P3a** under photoirradiation, we next investigated network polymer formation and its application as a photodegradable gel. A mixture of **P2** and bistrifluoroborate **4** in dimethylformamide (DMF) was treated dropwise with chlorotrimethylsilane (TMSCl). Gelation occurred within 1 min upon addition of TMSCl, and network formation was completed by thermal treatment at 80°C for 72 h. After washing with DMF and dichloromethane (DCM) and drying under reduced pressure, the crosslinked polymer **P6‐NW** was obtained (Figure 5).

**FIGURE 5:**
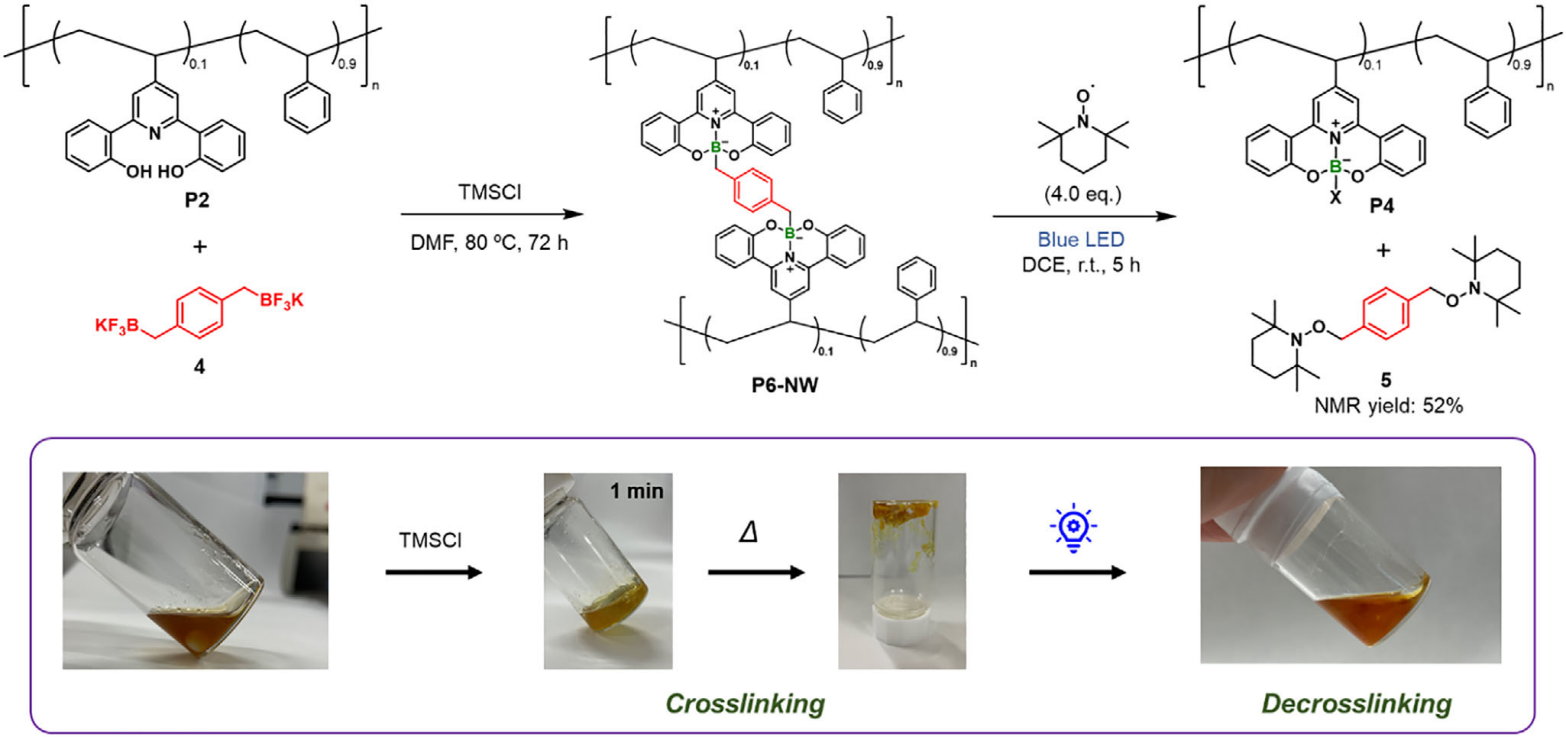
Gel formation via crosslinking of **P2** with bistrifluoroborate **4** in DMF. Blue‐light irradiation of the **P6‐NW** gel swollen in DCE in the presence of TEMPO resulted in network degradation through C─B bond cleavage, affording polymer fragments and the bis‐TEMPO adduct **5**. The B–X unit in **P4** represents an unidentified boron‐containing residue.

The photoinduced decrosslinking behavior of **P6‐NW** was then examined. The network polymer was swollen in DCE in the presence of TEMPO and irradiated with a blue LED for 5 h. The resulting mixture was dissolved in DCM and poured into methanol to induce reprecipitation. The collected yellow powder was identified as **P4** by ^1^H NMR analysis (Figure S12). ^1^H NMR analysis of the filtrate confirmed formation of the bis‐TEMPO adduct **5** in 52% NMR yield (Figures 5 and S13). These results demonstrate that compound **4** functions effectively as a crosslinker for **P2** and that alkyl radicals are generated under blue‐light irradiation even in the gel state. The released *para*‐xylylene biradical was trapped by TEMPO to afford adduct **5**.

Finally, the application of **P6‐NW** as an organic solvent absorbent and its subsequent photoinduced solvent release were investigated. Absorption tests were conducted by immersing dried **P6‐NW** samples in various liquids for 2 h. The absorption degree was calculated according to Equation (1): [29]

(1) $$\mathrm{Absorption}\;\mathrm{degree}\;\left(\%\right)=\frac{W_{s}\;-\;W_{d}}{W_{d}}\;\times \;100,$$

where *W*_s_ and *W*_d_ represent the weights of the swollen (after absorption) and dry (before absorption) **P6‐NW** samples, respectively. Figure 6a summarizes the absorption capacities of **P6‐NW** for nine liquids, including organic solvents, water, and pump oil. Consistent with its hydrophobic character, **P6‐NW** exhibited high affinity for organic solvents such as DCM, THF, DMF, toluene, and ethylbenzene, while showing minimal uptake of water. Based on these results, selective absorption of organic solvent from an organic solvent/water mixture was examined (Figure 6b). Dry **P6‐NW** (96 mg) was immersed in a biphasic mixture of toluene (0.2 mL) and distilled water (0.3 mL, colored with CuSO_4_) for 10 min. Subsequent filtration through a cotton plug separated the swollen **P6‐NW** gel from the aqueous phase, indicating preferential absorption of toluene into the polymer network. The resulting organogel was then irradiated with a blue LED for 2 h, leading to the release of absorbed toluene. Photoirradiation partially cleaved the PDP–borate crosslinks, inducing decrosslinking of the **P6‐NW** network and consequent solvent release. Conventional absorbent materials typically require thermal or vacuum treatment to release absorbed organic solvents, and volatile components are often lost during heating [30]. In contrast, this polymer–borate network forms organogels upon organic solvent uptake and enables solvent release under blue‐light irradiation at ambient temperature, demonstrating an alternative approach for photoresponsive absorbent materials.

**FIGURE 6:**
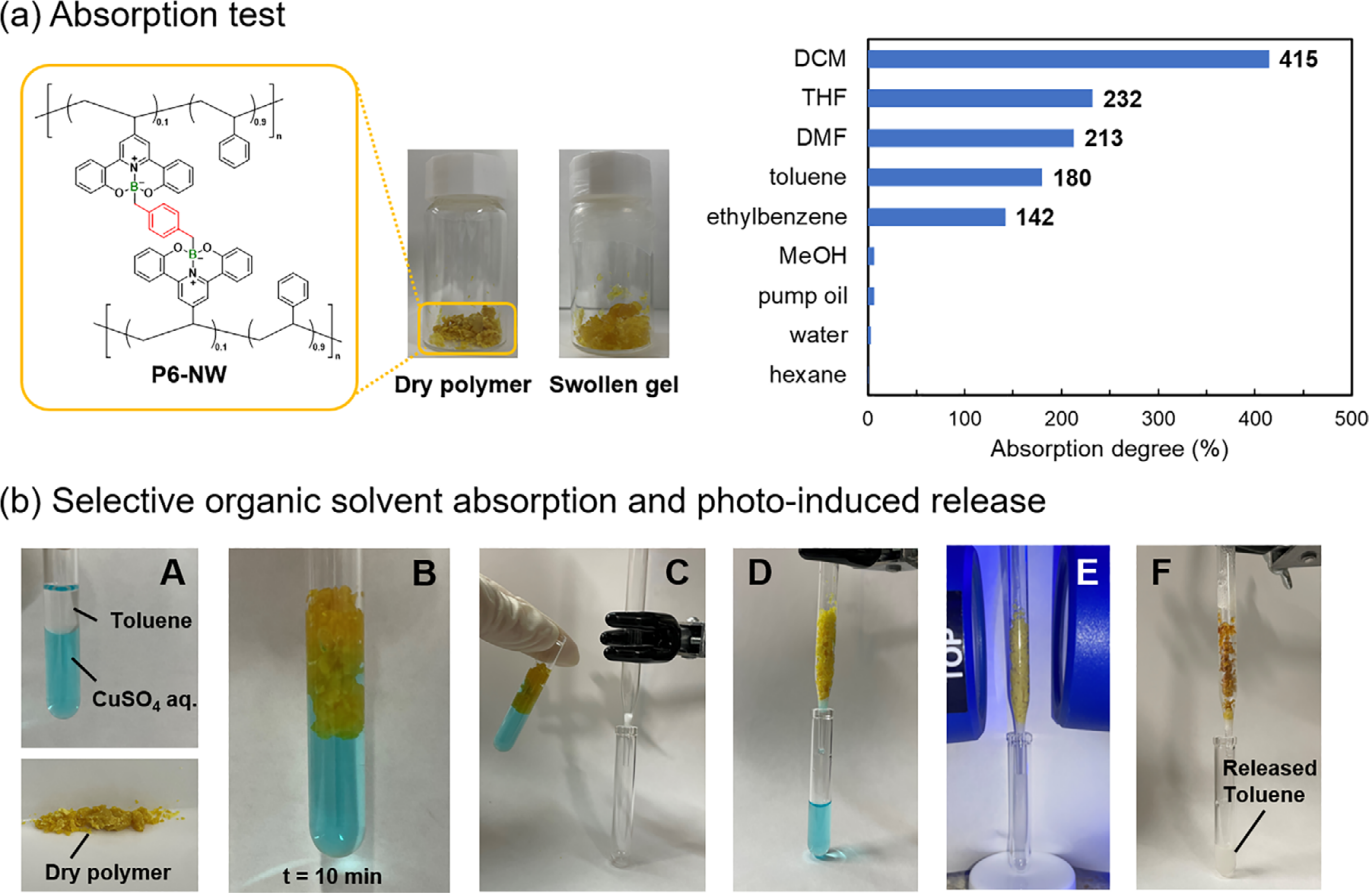
(a) Absorption capacities of **P6‐NW** for various solvents. (b) Demonstration of selective organic solvent absorption from an organic solvent/water mixture using **P6‐NW** and subsequent release of the absorbed organic solvent upon blue‐light irradiation.

## 3 Conclusion

In conclusion, we have developed polystyrene‐based polymers bearing PDP moieties that function as versatile platforms for photo‐driven alkyl radical generation and photodegradable materials. These PDP polymers form borate complexes with alkylboronic acids, which efficiently generate alkyl radicals under blue‐light irradiation for Giese‐type addition reactions. The polymer‐supported system enables straightforward purification by reprecipitation—a practical advantage over the column chromatography required for small‐molecule analogues—and facile separation of products from the polymer matrix. The PDP polymer can be recovered via fluoride treatment and regenerated through recomplexation (50% PDP recovery), demonstrating proof‐of‐concept for polymer recycling. While the recovery efficiency and reaction conditions require further optimization, this work establishes that the polymer‐supported approach transforms purification challenges into practical advantages.

Beyond radical generation, crosslinked PDP–borate networks operate as photodegradable organogels that selectively absorb organic solvents from water and release them upon light irradiation at ambient temperature, demonstrating an alternative approach to conventional thermal or vacuum‐based methods. Overall, this work demonstrates that PDP polymer–borate complexes are versatile materials with dual functionalities applicable to both synthetic radical chemistry and photoresponsive absorbent systems.

## Funding

Japan Society for the Promotion of Science.

## Conflicts of Interest

The authors declare no conflicts of interest.

## Supporting information



The authors have cited additional references within the Supporting Information [31, 32, 33, 34, 35, 36, 37, 38, 39, 40, 41]. **Supporting File**: chem71420‐sup‐0001‐SuppMat.pdf.

## Data Availability

The data that support the findings of this study are available in the Supporting Information of this article.
