# Supplementary material for: Dual‐Functional Polystyrene‐Based Borate Complexes: Recyclable Alkyl Radical Sources and Photodegradable Absorbents
Source: Chemistry. 2026 Jul 17;32(39):e71420. doi: 10.1002/chem.71420 (PMC13715469; doi:10.1002/chem.71420)
Supplement: Supplementary file 1 [file CHEM-32-e71420-s001.pdf]

Supporting Information

Dual-Functional Polystyrene-Based Borate Complexes: Recyclable Alkyl Radical Sources and Photodegradable Absorbents

Nami Jimba,[a] Ryosei Ishii,[a] Kazuki Wako,[a] Tomoki Hirohata,[a] Kosuke Sato,[a] Tomohiro Kubo,[b] Kotaro Satoh,[b] Yuto Sumida,[c] Hirohisa Ohmiya,[d] Shinsuke Inagi*[a]

[a] Department of Chemical Science and Engineering, School of Materials and Chemical Technology, Institute of Science Tokyo, 4259 Nagatsuta-cho, Midori-ku, Yokohama 226-8501, Japan

[b] Department of Chemical Science and Engineering, School of Materials and Chemical Technology, Institute of Science Tokyo, 2-12-1 Ookayama, Meguro-ku, Tokyo 152-8550, Japan

[c] Laboratory for Biomaterials and Bioengineering, Institute of Integrated Research, Institute of Science Tokyo, 2-3-10 Kanda-Surugadai, Chiyoda-ku, Tokyo 101-0062, Japan

[d] Graduate School of Engineering, Kyoto University, Kyoto University Katsura, Nishikyo-ku, Kyoto 615-8510, Japan

*E-mail: inagi@mct.isct.ac.jp

Table of Contents

- **Materials** **S2**
- **Instrumentation** **S4**
- **Monomer synthesis** **S6**
- **Polymer synthesis** **S7**
- **UV-vis spectra** **S10**
- **Radical trapping experiment** **S11**
- **TD-DFT calculations** **S14**
- **Giese addition** **S20**
- **Recycle use** **S23**
- **GPC profiles** **S27**
- **Network polymer formation** **S28**
- **NMR spectra** **S31**

■ Materials

All reagents and solvents were obtained from commercial sources including super dehydrated grade solvents and used without further purification unless otherwise noted.

Tokyo Chemical Industry Co., Ltd.

Benzyl acrylate (stabilized with MEHQ, purified with activated alumina prior to use)
Benzylidenemalononitrile
Bis(dibenzylideneacetone)palladium(0) ($Pd(dba)_2$)
1,4-Bis(chloromethyl)benzene
Bis[di-*tert*-butyl(4-dimethylaminophenyl)phosphine]dichloropalladium(II) ($(AMPhos)_2PdCl_2$)
Chlorotrimethylsilane (TMSCl)
2,6-Dibromopyridine
2,6-Dichloro-4-iodopyridine
Dimethyl fumarate
2-Methoxyphenylboronic acid
Palladium(II) acetate ($Pd(OAc)_2$)
2-Phenylethylboronic acid
Potassium hydrogen fluoride (KHF_2)
Pyridine hydrochloride
Tetrabutylammonium fluoride (*ca*. 1 mol/L in tetrahydrofuran) (TBAF)
4,4,5,5-Tetramethyl-2-vinyl-1,3,2-dioxaborolane
Triethylamine trihydrofluoride (Et_3N-3HF)
Trifluoromethanesulfonic anhydride (Tf_2O)
Tris(4-methoxyphenyl)phosphine

FUJIFILM Wako Pure Chemical Corporation

Alumina, activated
2,2'-Azobis(isobutyronitrile) (AIBN)
Copper(II) sulfate
Molecular Sieves 3A
Potassium acetate (KOAc)
Potassium carbonate (K_2CO_3)
Sodium carbonate (Na_2CO_3)
Styrene monomer (purified with activated alumina prior to use)
Triethylamine (NEt_3)
Tripotassium phosphate (K_3PO_4)
Nitromethane
1,1,2,2-Tetrachloroethane

1,4-Dioxane
Dimethyl sulfoxide (DMSO)
N,*N*-Dimethylformamide
Tetrahydrofuran (THF)
Tetrahydrofuran (THF) stabilizer free
Toluene

Kanto Chemical Co., Inc.

Chloroform-d_1 99.8% D (0.03% TMS included, with silver foil) ($CDCl_3$)
Dichloromethane-d_2, 99.8 atom % D (CD_2Cl_2)
1,2-Dichloroethane (DCE)
Ethyl acetate (EtOAc)
^{n}Hexane

Sigma-Aldrich

[1,1'-Bis(diphenylphosphino)ferrocene]palladium(II) Dichloride (Pd(dppf)Cl_2)
Cesium fluoride (CsF)
2,2,6,6-Tetramethylpiperidine 1-oxyl free radical (TEMPO)
Benzotrifluoride

Angene International Limited

Bis(pinacolato)diboron

EURISO-TOP

Dimethyl sulfoxide-d_6 (+ 0.06% TMS) (DMSO-d_6)

Shin-Etsu Chemical Co., Ltd.

Chloroform ($CHCl_3$)

AGC Inc.

Dichloromethane (DCM)

Godo Chemical Co., Ltd.

Methanol (MeOH)

■ Instrumentation

NMR measurement

Measurements were performed using a nuclear magnetic resonance (NMR) spectrometer (Bruker Biospin, AVANCE III HD500) with the frequencies shown as follows: ^{1}H NMR (500.13 MHz), ^{13}C NMR (125.76 MHz), ^{11}B NMR (128.38 MHz or 160.46 MHz), and ^{19}F NMR (470.59 MHz). Internal standards used for each solvent are as follows:

$CDCl_3$; ^{1}H: tetramethylsilane (TMS) (δ = 0.0 ppm), ^{13}C: $CDCl_3$ (δ = 77.16 ppm), ^{19}F: C_6H_5F (δ = −63.2 ppm)

CD_2Cl_2; ^{1}H: CH_2Cl_2 (δ = 5.33 ppm), ^{13}C: CD_2Cl_2 (δ = 54.24 ppm)

DMSO-d_6; ^{1}H: DMSO (δ = 2.50 ppm), ^{13}C: DMSO-d_6 (δ = 39.52 ppm)

Additionally, for qualitative analysis, the ^{11}B NMR spectrum is presented as a magnitude spectrum.

GPC measurement

The number-average molecular weight (M_n) and molecular weight distribution (M_w/M_n) of the product polymers were determined by gel permeation chromatography (GPC) in DMF containing 100 mM LiCl at 40 °C using two hydrophilic polymer gel columns [Tosoh α-M and α-3000 (7.8 mm i.d. × 30 cm); flow rate 1.0 mL min^{-1}] connected to a JASCO PU-2080 precision pump and a JASCO RI-2031 and UV-2075 detectors. The columns were calibrated against polystyrene standards (Agilent Technologies; M_p = 580–3209000, M_w/M_n = 1.02–1.12).

ESI-TOF MS measurement

ESI-TOF-MS measurements were performed on Bruker micrOTOFII.

Melting point measurement

Melting points were measured using a Yanaco MP-500P micro melting point apparatus.

UV-vis measurement

UV-vis spectra were measured using a Shimadzu UV-1800 spectrophotometer with a quartz glass cell containing a DCE solution of the sample (0.2 mg/mL or 2 × 10^{-4} M).

Photochemical reaction

A Kessil PR-160L 440 nm lamp was used as the light source for photoirradiation experiments. Two lamps were used to irradiate the sample from both sides at maximum intensity. A Sanwa Supply VEH-FAN1 was used as a cooling fan (Figure S1).

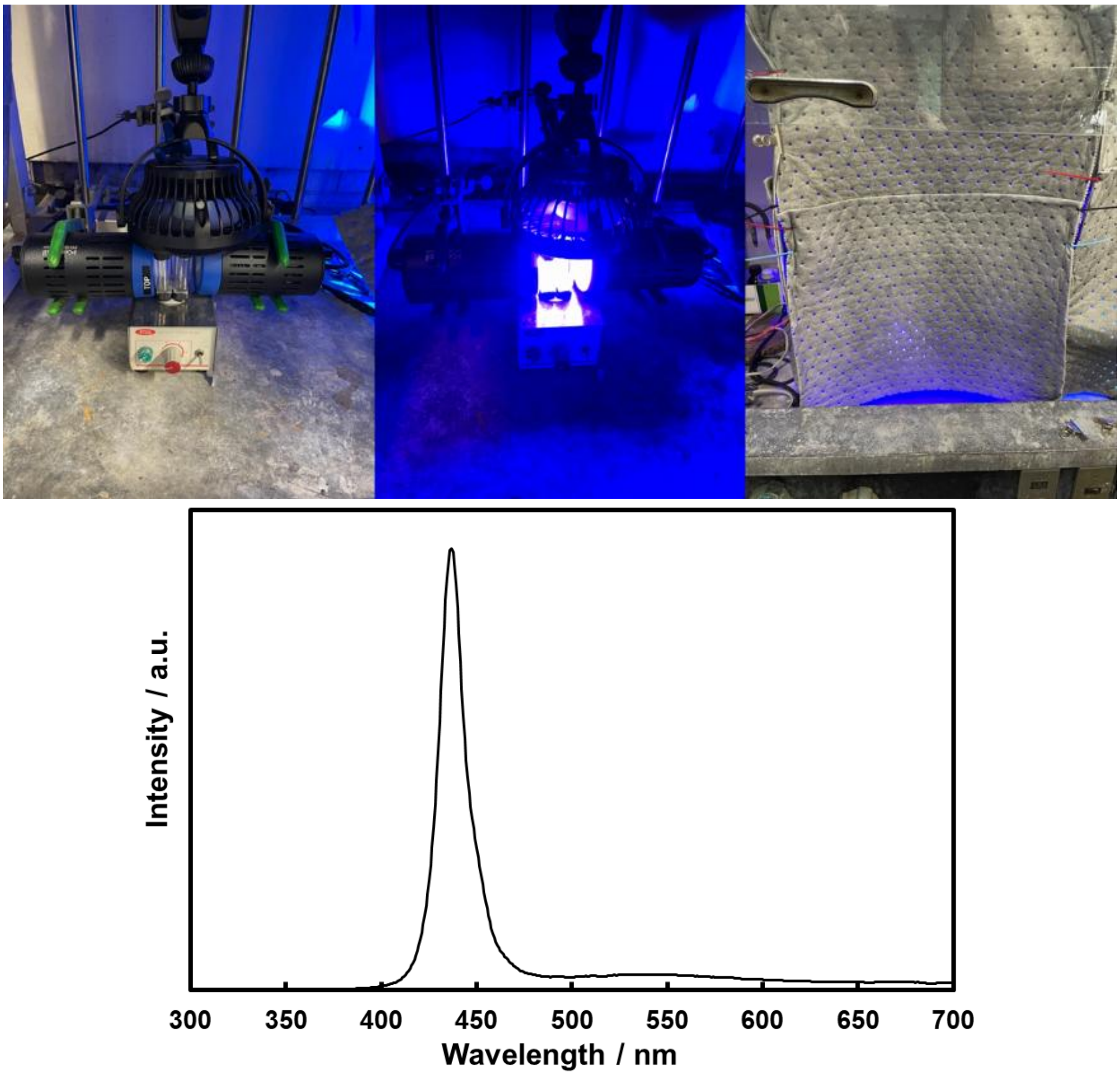

Figure S1. Photographs of the setup for light irradiation and a spectrum of the light used.

■ Monomer synthesis

2,6-Bis(2-methoxyphenyl)-4-vinylpyridine (S1)

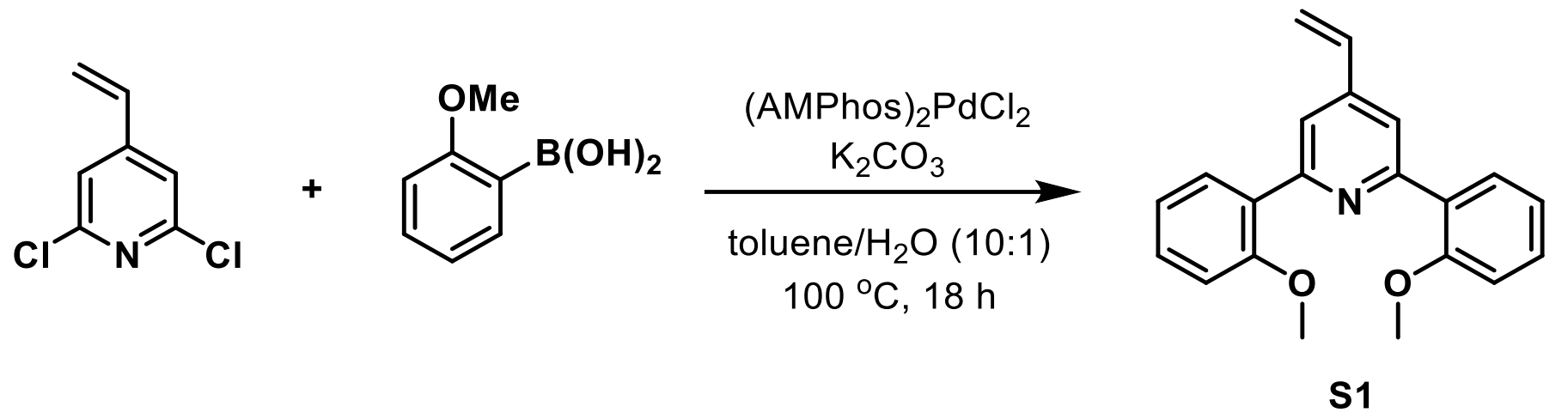

Scheme S1. Synthesis of monomer **S1**.

2,6-Dichloro-4-vinylpyridine was prepared from 2,6-dichloro-4-iodopyridine according to the literature.[31] To a 1 L two-necked round-bottom flask were added 2,6-dichloro-4-vinylpyridine (450.3 mg, 2.59 mmol, 1.0 eq), 2-methoxyphenylboronic acid (1180.44 mg, 7.77 mmol, 3.0 eq), $(AMPhos)_2PdCl_2$ (94.94 mg, 0.134 mmol, 0.05 eq), and K_2CO_3 (719.2 mg, 5.18 mmol, 2.0 eq). The flask was dried under vacuum and backfilled with Ar. Ar-bubbled toluene/H_2O (100 mL/10 mL) was added, and the mixture was heated at reflux with stirring at 100 °C for 18 h. After cooling to room temperature, distilled water was added and the mixture was extracted with EtOAc. The organic layer was washed with brine, dried over anhydrous Na_2SO_4, and filtered through Celite. The solvent was removed under reduced pressure. Purification by silica gel column chromatography (eluent: hexane/EtOAc/Et_3N = 20:1:0.2) afforded a white solid (711.52 mg, 2.24 mmol, 87%).

S1: white solid; m.p. 115.1–115.9 °C; ^{1}H NMR (400.13 MHz, $CDCl_3$, ppm): δ = 7.89 (dd, *J* = 7.6, 1.8 Hz, 2H, Ar*H*), 7.75 (s, 2H, Py*H*), 7.36 (ddd, *J* = 8.2, 8.2, 1.8 Hz, 2H, Ar*H*), 7.08 (ddd, *J* = 7.5, 7.5, 0.9 Hz, 2H, Ar*H*), 7.01 (d, *J* = 8.2 Hz, 2H, Ar*H*), 6.79 (dd, *J* = 17.6, 10.8 Hz, 1H, C*H*(CH_2)), 6.00 (d, *J* = 17.5 Hz, 1H, CH(CH_2)), 5.48 (d, *J* = 11.0 Hz, 1H, CH(CH_2)), 3.89 (s, 6H,O CH_3); ^{13}C NMR (125.76 MHz, $CDCl_3$, ppm): δ =157.0, 1565.9, 144.1, 135.8, 131.5, 129.7, 129.5, 121.0, 120.4, 117.7, 111.4, 55.7; HRMS (ESI-TOF) calcd for $C_{21}H_{20}NO_2$ ($[M]^+$) *m/z* 318.1489, found 318.1486

■ Polymer synthesis

Poly(2,6-bis(2-methoxyphenyl)-4-vinylpyridine-co-styrene) (P1)

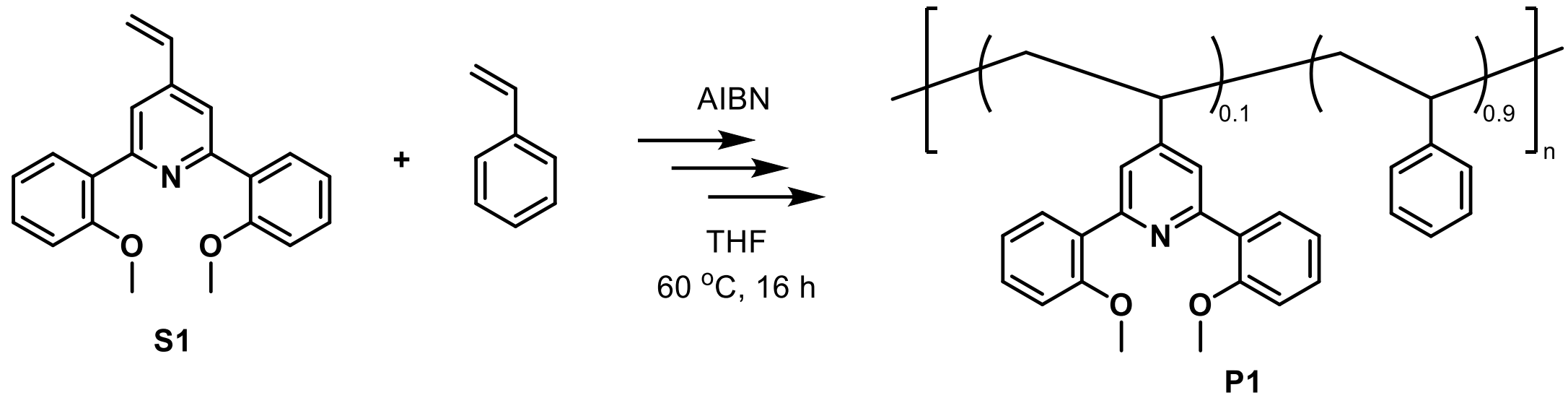

Scheme S2. Synthesis of **P1** by radical copolymerization of S1 and styrene.

To a Schlenk tube were added **S1** (800.8 mg, 2.52 mmol, 1.0 eq) and AIBN (207.4 mg, 1.26 mmol, 0.5 eq). The tube was dried under vacuum and backfilled with Ar. Ar-bubbled styrene (2362.1 mg, 22.7 mmol, 9.0 eq) and THF (2.4 mL) were added, and the mixture was heated at reflux with stirring at 60 °C for 16 h. After cooling to room temperature, the mixture was dissolved in chloroform and reprecipitated using methanol as a poor solvent. After decantation, the precipitate was collected by filtration to afford a white solid (2937.9 mg, 2.34 mmol, 93%).

P1: white solid; ^{1}H NMR (500.13 MHz, CD_2Cl_2, ppm): δ = 7.73 (br), 7.34 (br), 7.04 (br), 6.57 (br), 3.66 (br), 1.79 (br), 1.46 (br); ^{13}C NMR (125.76 MHz, CD_2Cl_2, ppm): δ =157.5, 155.3, 145.8, 131.8, 129.9, 128.36, 128.3, 127.9, 126.1, 123.2, 121.1, 111.8, 55.9, 46.6, 44.8, 43.9, 42.5, 40.8. M_n = 10,700, M_w = 25,200, $Đ$ = 2.4 (GPC relative to polystyrene standards); elemental analysis (%): calcd.: C 88.93, H 7.30, N 1.15, O 2.63, found: C 88.41, H 7.21, N 1.76, O 2.69.

Poly(2,2'-(4-vinylpyridine-2,6-diyl)diphenol-co-styrene) (P2)

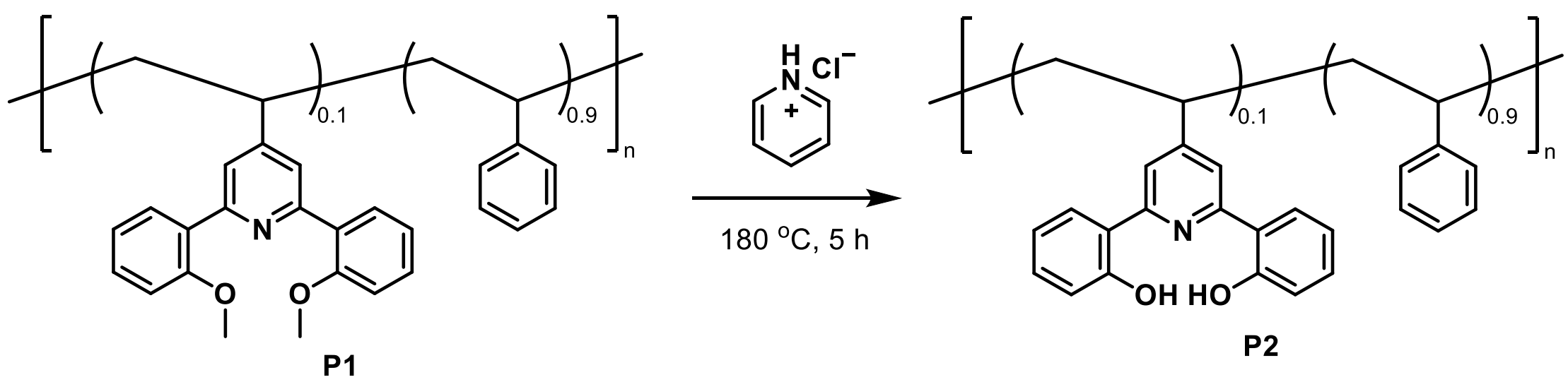

Scheme S3. Synthesis of **P2** from **P1**.

To a 50 mL round-bottom flask were added **P1** (805.4 mg, 0.642 mmol) and pyridine hydrochloride (21.97 g, 190 mmol). The flask was dried under vacuum and backfilled with Ar. The mixture was stirred at 180 °C for 5 h. After cooling to room temperature, the mixture was extracted with DCM, washed with saturated aqueous Na_2CO_3 solution, dried over anhydrous Na_2SO_4, and filtered. The solvent was removed under reduced pressure. The residue was dissolved in chloroform and reprecipitated using methanol as a poor solvent. After decantation, the precipitate was collected by filtration to afford a yellow solid (1.226 g, 0.488 mmol, 70%).

P2: yellow solid; ^{1}H NMR (500.13 MHz, CD_2Cl_2, ppm): δ = 7.33 (br), 7.04 (br), 6.57 (br), 1.79 (br), 1.47 (br); ^{13}C NMR (125.76 MHz, CD_2Cl_2, ppm): δ =157.0, 155.8, 145.7, 131.7, 128.6, 128.4, 128.3, 128.0, 127.9, 126.3, 126.1, 125.9, 122.1, 120.2, 119.9, 118.0, 46.4, 44.4, 40.8. M_n = 16,500, M_w = 70,700, $Đ$ = 4.3 (GPC relative to

polystyrene standards); elemental analysis (%): calcd.: C 89.01, H 7.13, N 1.18, O 2.69, found: C 88.28, H 7.11, N 1.76, O 2.72.

Poly(PDP borate) (P3a)

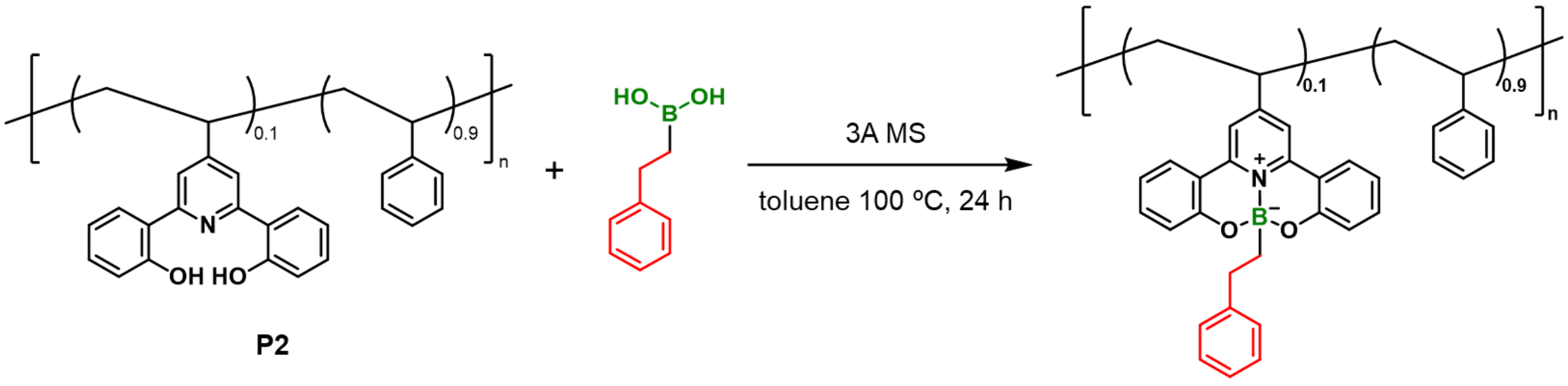

Scheme S4. Borate complex formation of **P2** with 2-phenylethylboronic acid.

To a Schlenk tube were added **P2** (850.0 mg, 0.693 mmol, 1.0 eq), 2-phenylethylboronic acid (208.4 mg, 1.39 mmol, 2.0 eq), and molecular sieves 3A. The tube was dried under vacuum and backfilled with Ar. Ar-bubbled toluene (1 mL) was added, and the mixture was stirred at 100 °C for 24 h. After cooling to room temperature, the mixture was dissolved in chloroform and reprecipitated using methanol as a poor solvent. After decantation, the precipitate was collected by filtration to afford a yellow solid (754.7 mg, 0.563 mmol, 81%).

P3a: yellow solid; ^{1}H NMR (500.13 MHz, CD_2Cl_2, ppm): δ = 7.40 (br), 7.05 (br), 6.58 (br), 2.49 (br), 1.79 (br), 1.46 (br), 0.57 (br); ^{13}C NMR (125.76 MHz, CD_2Cl_2, ppm): δ =157.7, 147.1, 145.7, 134.01, 128.4, 128.2, 126.1, 125.1, 120.8, 119.7, 118.8, 117.3, 46.5, 44.4, 40.8, 30.8; ^{11}B NMR (160.46 MHz, CD_2Cl_2, ppm): δ = 2.01. M_n = 13,100, M_w = 51,300, PDI = 3.9 (GPC relative to polystyrene standards); elemental analysis (%): calcd.: C 88.68, H 7.06, N 1.09, O 2.50, B 0.66, found: C 87.04, H 6.93, N 1.62. B prevented detection of O.

Poly(PDP borate) (P3b)

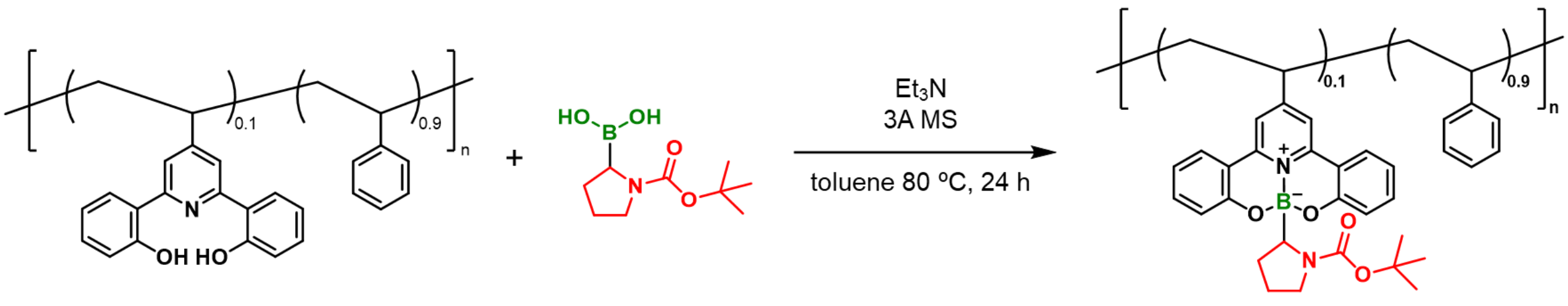

Scheme S5. Borate complex formation of **P2** with 1-*N*-Boc-pyrrolidin-2-ylboronic acid.

To a Schlenk tube were added **P2** (470.0 mg, 0.395 mmol, 1.0 eq), 1-*N*-Boc-pyrrolidin-2-ylboronic acid (170.7 mg, 0.794 mmol, 2.0 eq), Et_3N (0.766 mL, 5.52 mmol, 14 eq), and molecular sieves 3A. The tube was dried under vacuum and backfilled with Ar. Ar-bubbled toluene (5 mL) was added, and the mixture was stirred at 80 °C for 24 h. After cooling to room temperature, the mixture was reprecipitated using methanol as a poor solvent. After decantation, the precipitate was collected by filtration to afford a yellow solid (436.1 mg, 0.333 mmol, 85%).

P3b: yellow solid; ^{1}H NMR (500.13 MHz, CD_2Cl_2, ppm): δ = 7.45 (br), 7.37 (br), 7.07 (br), 6.50 (br), 6.51 (br), 3.09 (br), 2.94 (br), 1.81 (br), 1.47 (br), 1.08 (br), 1.02 (br), 0.90 (br), 0.85 (br); ^{13}C NMR (125.76 MHz, CD_2Cl_2, ppm): δ =145.3, 133.6, 128.0, 125.7, 119.8, 46.0, 44.4, 40.4, 28.3; ^{11}B NMR (128.38 MHz, CD_2Cl_2, ppm): δ = 1.78. M_n = 17,100, M_w = 86,400, PDI = 5.0 (GPC relative to polystyrene standards); elemental analysis (%): calcd.: C 86.38, H 7.24, N 1.78, O 4.06, B 0.54, found: C 85.10, H 7.14, N 2.35. B prevented detection of O.

■ **UV-vis spectra**

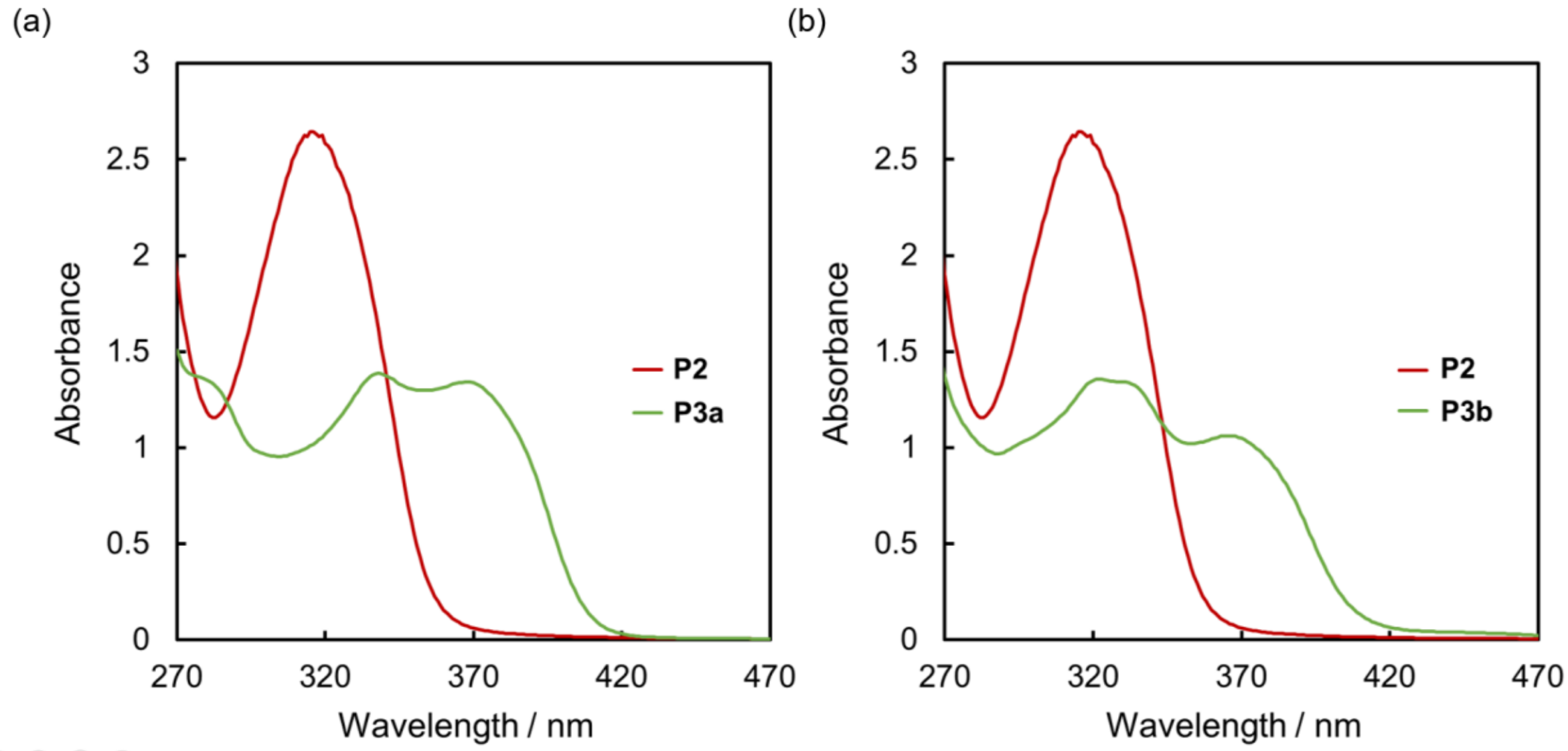

Figure S2. UV–vis absorption spectra of **P2** and **P3** measured in a 0.2 mg/mL CH_2Cl_2 solution. (a) **P3a** with phenylethylboronic acid and (b) **P3b** with 1-*N*-Boc-pyrrolidin-2-ylboronic acid were used as alkyl radical sources.

■ Radical trapping experiment

Photochemical reaction of P3a in the presence of TEMPO

Table S1. Optimization of solvents for photochemical reaction of **P3a** in the presence of TEMPO.

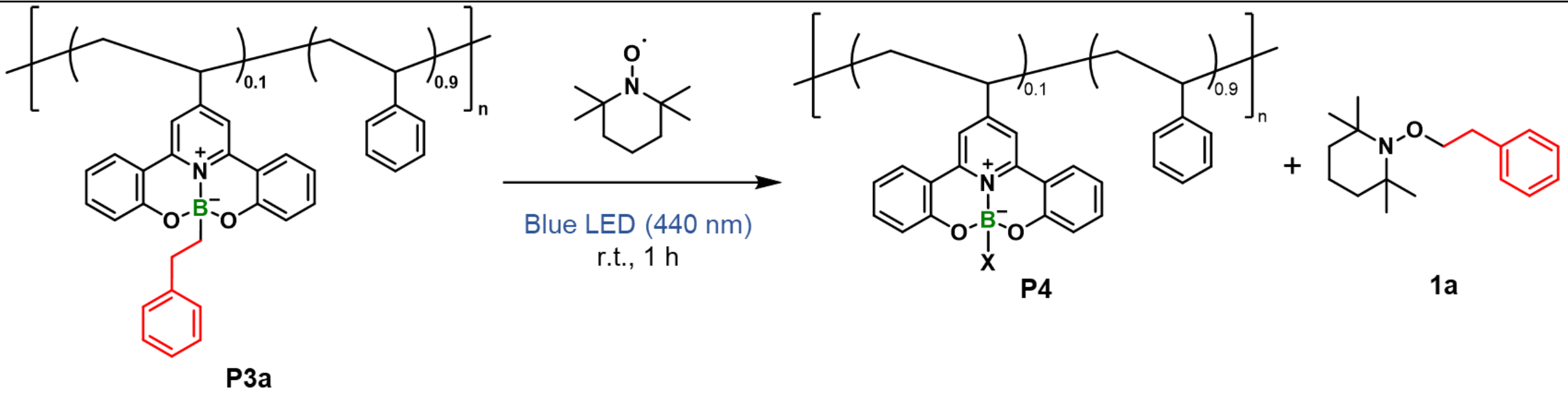

Entry	Solvent	Yield of **1a** (%)[a]
1	Dichloromethane (DCM)	72
2	1,4-Dioxane	53
3	1,2-Dichloroethane (DCE)	89

[a]Determined by ^{1}H NMR using nitromethane as the internal standard. B–X in **P4** is an unidentified boron-residue.

Typical reaction conditions are as follows. To a 4 mL vial were added **P3a** (41.04 mg, 0.0183 mmol, 1.0 eq), TEMPO (5.90 mg, 0.0378 mmol, 2.1 eq), and DCE (2 mL). The vial was sealed with a rubber septum and bubbled with Ar for 5 min. The mixture was stirred at room temperature for 1 h under irradiation with 440 nm light. The solvent was removed under reduced pressure, and the residue was dried under vacuum to afford the crude product. **1a** was obtained in 89% NMR yield. The ^{1}H NMR signals of the product were consistent with those reported in the literature.[32]

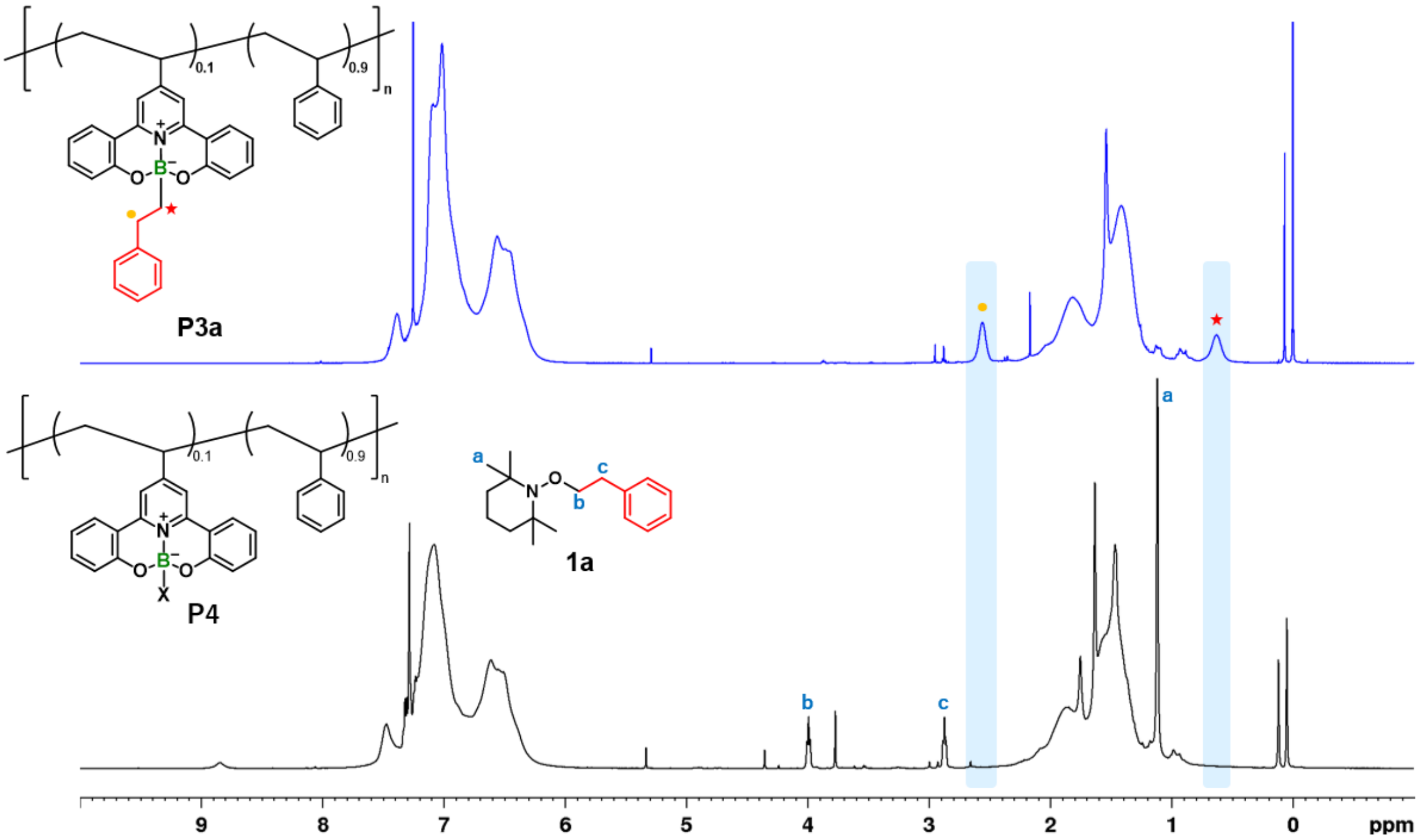

Figure S3. ^{1}H NMR spectra (500.13 MHz, $CDCl_3$) of **P3a** before and after blue light irradiation in the presence of TEMPO. B–X in **P4** is an unidentified boron-residue.

Photochemical reaction of P3b in the presence of TEMPO

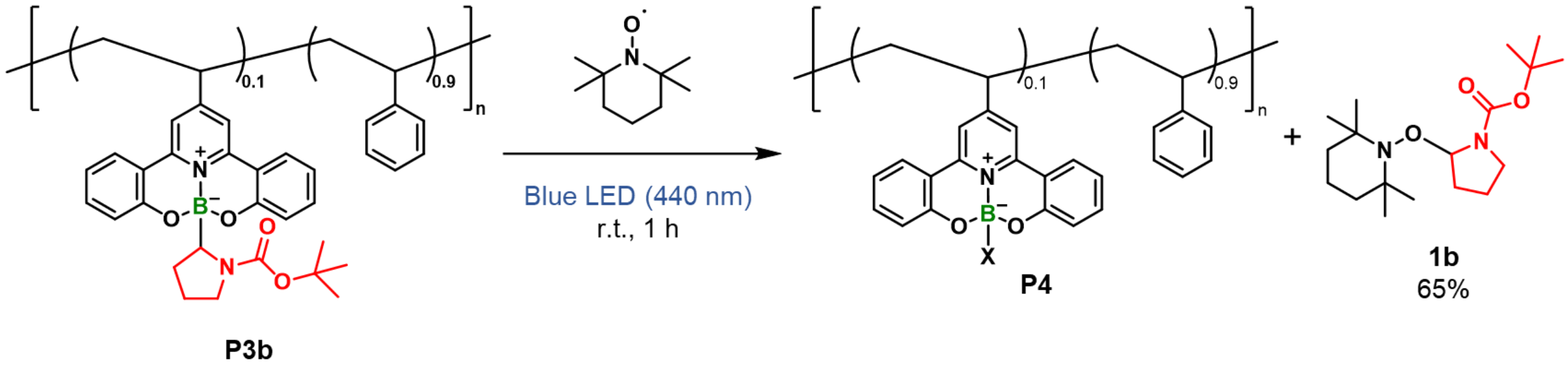

Scheme S6. Photochemical reaction of **P3b** in the presence of TEMPO. B–X in **P4** is an unidentified boron-residue.

To a 4 mL vial were added **P3b** (37.57 mg, 0.0186 mmol, 1.0 eq), TEMPO (6.37 mg, 0.0407 mmol, 2.2 eq), and DCE (2 mL). The vial was sealed with a rubber septum and bubbled with Ar for 5 min. The mixture was stirred at room temperature for 1 h under irradiation with 440 nm light. The solvent was removed under reduced pressure, and the residue was dried under vacuum to afford the crude product. **1b** was obtained in 65% NMR yield. The ^{1}H NMR signals of the product were consistent with those reported in the literature.[33]

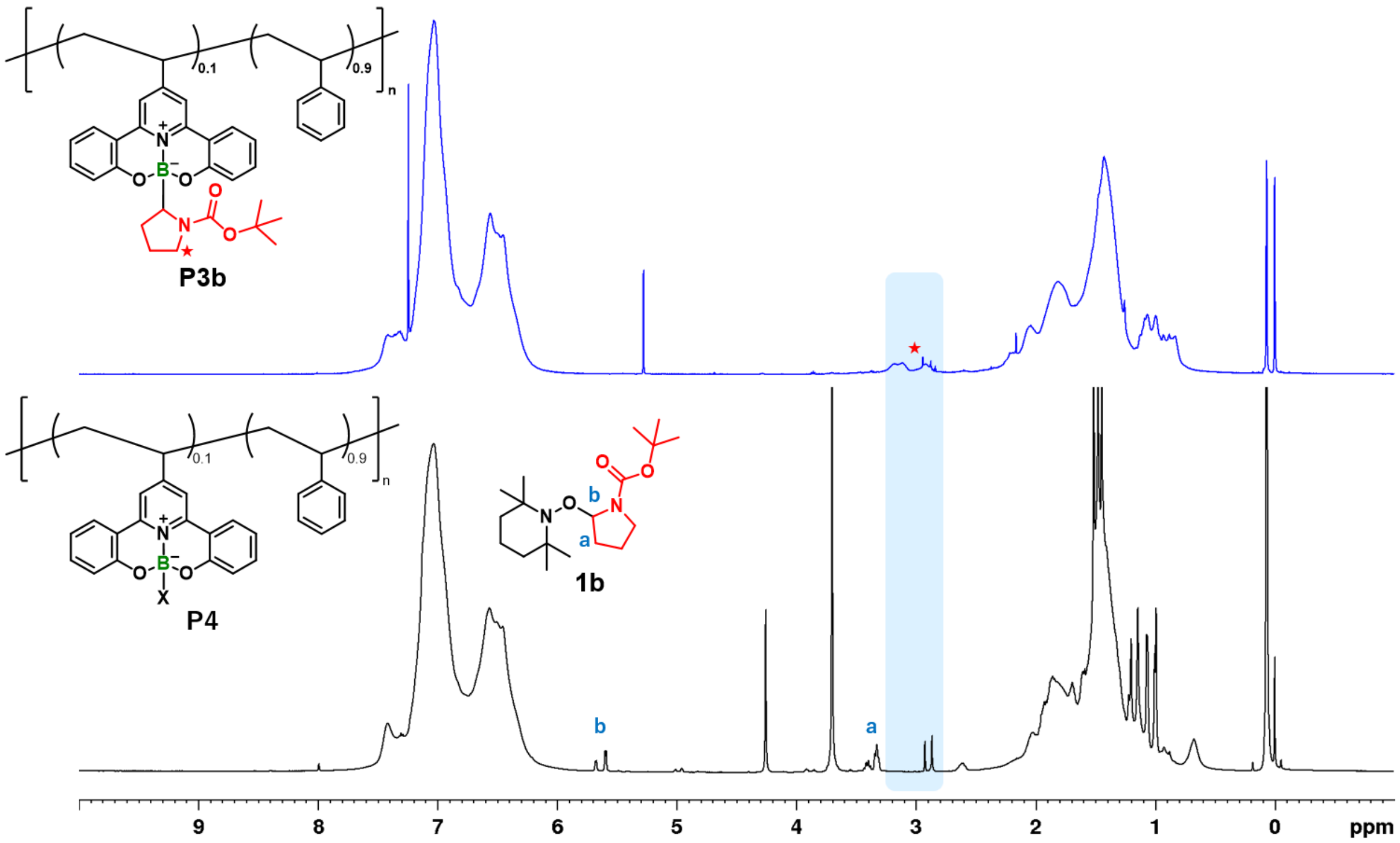

Figure S4. ^{1}H NMR spectra (500.13 MHz, $CDCl_3$) of **P3b** before and after blue light irradiation in the presence of TEMPO. B–X in **P4** is an unidentified boron-residue.

■ TD-DFT Calculations

Mechanistic insight into photoinduced C–B bond cleavage

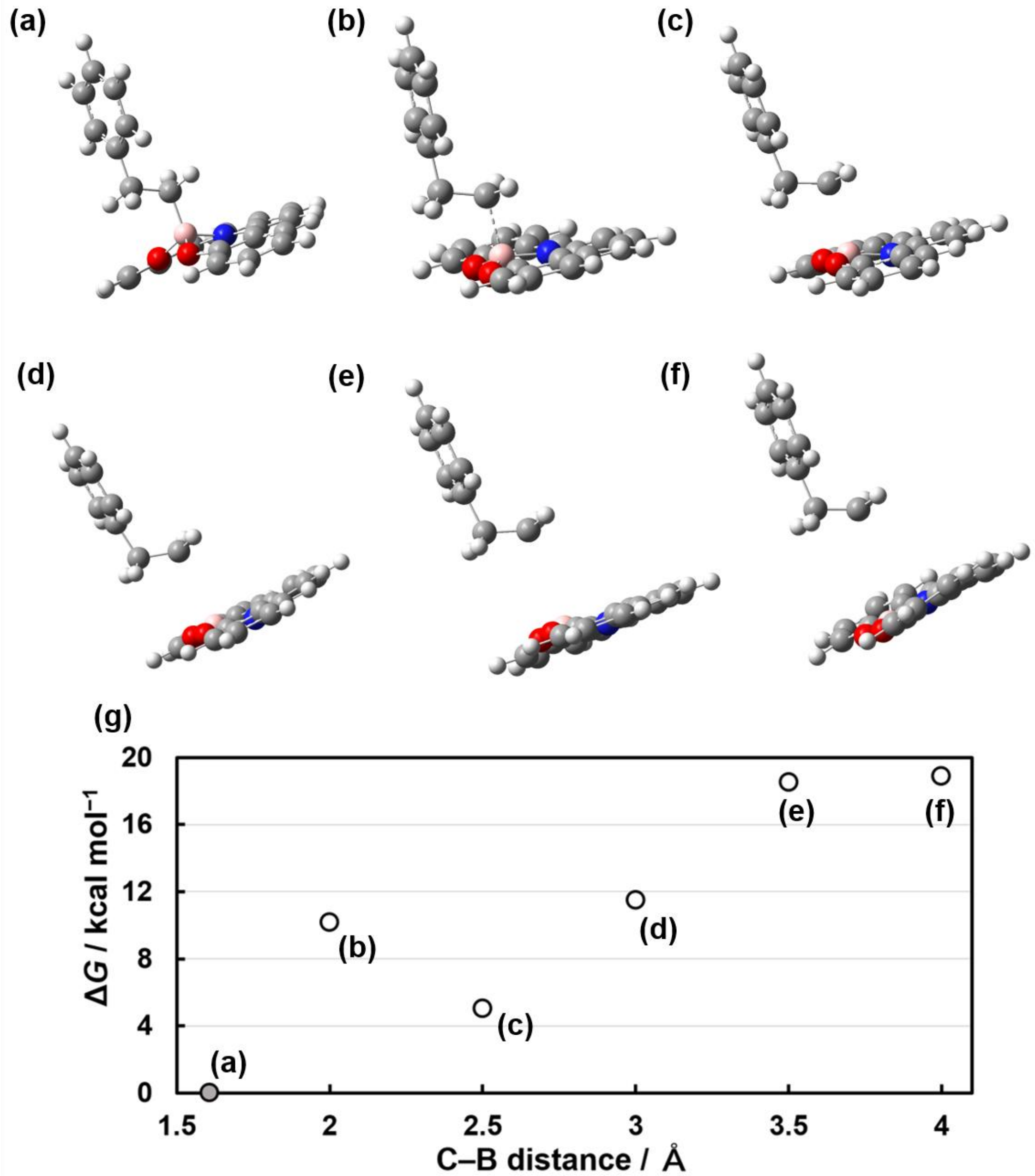

Figure S5. (a) Optimized structure of the PDP-phenylethylborate complex at S_1 excited state. (b–d) Relaxed structures with C–B distance of (b) 2.0 Å, (c) 2.5 Å, (d) 3.0 Å, (e) 3.5 Å (f) 4.0 Å at the S_1 state. (e) Gibbs free energy as a function of C–B distance under standard conditions. All TD-DFT calculatios were performed at the CAM-B3LYP/6-31G+(d,p) level of theory.

In the optimized structure (c), the PDP moiety adopts a planer conformation, which corresponds to a decrease in Gibbs free energy. In structure (f), the phenylethyl group is significantly separated from the PDP borate moiety, and this structure can be regarded as the radical-releasing state capable of subsequent reactions. The relatively low Gibbs free energy of this state (+18.9 kcal mol^{-1}) is consistent with efficient generation of the reactive radical even at room temperature.

**(a)**				**(b)**			
Symbol	X	Y	Z	Symbol	X	Y	Z
C	1.978865	1.20468	-0.929226	C	2.130079	-1.20572	0.740907
C	1.978806	-1.204765	-0.92923	C	2.130068	1.205745	0.740904
C	2.685844	-1.200032	-2.126608	C	2.995068	1.212845	1.876697
C	3.022132	-0.000064	-2.760738	C	3.398003	0.000018	2.407246
C	2.685899	1.199917	-2.126606	C	2.995076	-1.21281	1.876701
H	3.003682	-2.138299	-2.56151	H	3.345892	2.143356	2.292576
H	3.56756	-0.000076	-3.695707	H	4.061791	0.000021	3.26768
H	3.003777	2.138171	-2.561509	H	3.345903	-2.14332	2.292587
O	0.573907	1.158981	1.553535	O	0.642999	-1.16533	-1.66098
O	0.573859	-1.159004	1.553535	O	0.642989	1.16533	-1.66098
C	1.683214	2.405814	-0.179213	C	1.658499	-2.41634	0.105507
C	1.042198	2.300258	1.101533	C	0.933834	-2.36814	-1.10272
C	2.058375	3.697996	-0.604265	C	1.926533	-3.6992	0.631595
C	0.894396	3.437736	1.920406	C	0.51616	-3.51266	-1.76284
C	1.870286	4.806798	0.192737	C	1.504815	-4.84661	-0.01986
H	2.519013	3.829353	-1.57569	H	2.467261	-3.79521	1.564021
C	1.296741	4.675021	1.474157	C	0.80251	-4.76291	-1.2235
H	0.429226	3.297368	2.889681	H	-0.02575	-3.40137	-2.69556
H	2.174389	5.784133	-0.165993	H	1.726646	-5.81658	0.412791
H	1.162208	5.549985	2.101256	H	0.479304	-5.66231	-1.73604
C	1.683091	-2.40589	-0.179223	C	1.658473	2.416356	0.105506
C	2.058183	-3.698089	-0.604287	C	1.926492	3.699214	0.631594
C	1.042088	-2.300307	1.101522	C	0.933809	2.368149	-1.10272
C	1.870041	-4.806887	0.192707	C	1.504762	4.846624	-0.01986
H	2.518808	-3.829462	-1.575716	H	2.467217	3.795237	1.564021
C	0.894232	-3.437781	1.920388	C	0.516123	3.512665	-1.76284
C	1.29651	-4.675085	1.47413	C	0.802458	4.762912	-1.22351
H	2.174091	-5.784236	-0.166031	H	1.726581	5.816597	0.41279
H	0.429075	-3.297396	2.889668	H	-0.02579	3.401371	-2.69557
H	1.161934	-5.550044	2.101225	H	0.479244	5.662307	-1.73604
C	-1.024315	0.000028	-0.09144	C	-0.98881	-4E-06	0.081393
H	-1.093781	-0.873292	-0.756036	H	-0.85953	0.911021	0.663378
H	-1.09374	0.873363	-0.75602	H	-0.85953	-0.91103	0.663374
C	-2.22669	0.000045	0.867962	C	-2.13573	-5E-06	-0.889
H	-2.162911	0.876328	1.522531	H	-2.08591	-0.88194	-1.53187
H	-2.162946	-0.876251	1.522517	H	-2.08592	0.88193	-1.53186

B	0.406495	-0.000012	0.645956	B	0.742419	0.000003	-0.92003
N	1.572644	-0.000036	-0.366295	N	1.746925	0.000008	0.226945
C	-3.560233	0.000076	0.160923	C	-3.43522	-1.4E-05	-0.10448
C	-4.18735	1.197978	-0.190707	C	-4.03484	-1.20192	0.27885
C	-4.187484	-1.197792	-0.190575	C	-4.03485	1.201883	0.278863
C	-5.398896	1.201914	-0.875606	C	-5.21272	-1.20355	1.018635
H	-3.71923	2.140984	0.080374	H	-3.57853	-2.14382	-0.01326
C	-5.399032	-1.201665	-0.875475	C	-5.21273	1.203496	1.018648
H	-3.719472	-2.140821	0.080608	H	-3.57855	2.143794	-0.01324
C	-6.010198	0.000139	-1.222027	C	-5.80502	-0.00003	1.391228
H	-5.868957	2.145817	-1.135202	H	-5.67049	-2.14616	1.30143
H	-5.869199	-2.145545	-1.134967	H	-5.67051	2.1461	1.301453
H	-6.95686	0.000164	-1.753173	H	-6.7249	-3.7E-05	1.9669

(c)

Symbol	X	Y	Z
C	2.209029	-1.213573	0.816868
C	2.208914	1.213733	0.816869
C	2.922549	1.202172	2.001588
C	3.309123	0.00013	2.584749
C	2.922663	-1.201949	2.001586
H	3.157166	2.132208	2.50131
H	3.892355	0.000157	3.498088
H	3.157371	-2.131964	2.501305
O	0.618548	-1.182166	-1.518136
O	0.618451	1.18218	-1.518144
C	1.820237	-2.431294	0.125436
C	1.095556	-2.35451	-1.075116
C	2.202224	-3.720894	0.567397
C	0.787536	-3.502259	-1.817405
C	1.895029	-4.84999	-0.154113
H	2.743474	-3.827699	1.500048
C	1.189231	-4.739803	-1.367353
H	0.220293	-3.375786	-2.73275
H	2.197585	-5.826108	0.208485
H	0.958401	-5.631106	-1.941069
C	1.820021	2.431416	0.125431
C	2.201894	3.721052	0.567391

(d)

Symbol	X	Y	Z
C	2.239039	-1.20953	0.839774
C	2.238915	1.20969	0.839773
C	2.782993	1.205517	2.117709
C	3.097891	0.000124	2.717596
C	2.783118	-1.2053	2.117707
H	2.925041	2.132565	2.654227
H	3.532294	0.000146	3.711621
H	2.925262	-2.13234	2.654224
O	0.884809	-1.16862	-1.57742
O	0.88471	1.168647	-1.57743
C	1.911174	-2.42441	0.146749
C	1.301833	-2.34991	-1.11531
C	2.214679	-3.71768	0.648183
C	0.996081	-3.49703	-1.85786
C	1.935564	-4.84168	-0.08405
H	2.673082	-3.82824	1.623444
C	1.325056	-4.73217	-1.35537
H	0.499803	-3.37301	-2.81384
H	2.180104	-5.81888	0.316993
H	1.110192	-5.62498	-1.93245
C	1.910943	2.424536	0.146745
C	2.214323	3.717837	0.648178

C	1.095358	2.354567	-1.075127	C	1.301625	2.349977	-1.11532
C	1.894609	4.850118	-0.154126	C	1.935114	4.841805	-0.08406
H	2.743126	3.827905	1.500047	H	2.672702	3.828439	1.623444
C	0.787248	3.502285	-1.817424	C	0.995778	3.497072	-1.85789
C	1.188832	4.739865	-1.367373	C	1.324633	4.73224	-1.35539
H	2.197077	5.826264	0.20847	H	2.179559	5.819028	0.31698
H	0.220023	3.375759	-2.732774	H	0.499522	3.373002	-2.81387
H	0.95793	5.631147	-1.941095	H	1.109695	5.625031	-1.93247
C	-1.300026	-0.000057	0.376877	C	-1.52504	-6.2E-05	0.455067
H	-1.22958	0.90681	0.981576	H	-1.50975	0.901838	1.068222
H	-1.229519	-0.906923	0.98157	H	-1.50969	-0.90196	1.068217
C	-2.397584	-0.00009	-0.650654	C	-2.57538	-9.4E-05	-0.63415
H	-2.296409	-0.876753	-1.303039	H	-2.45107	-0.87732	-1.28184
H	-2.296458	0.876575	-1.303042	H	-2.45112	0.877135	-1.28184
B	0.869496	0.000019	-0.865369	B	1.132928	0.000028	-0.93605
N	1.979633	0.000069	0.159007	N	2.184021	0.000076	0.151438
C	-3.794002	-0.000127	-0.055252	C	-3.99768	-0.00013	-0.10217
C	-4.449988	-1.197991	0.239318	C	-4.66658	-1.19737	0.168061
C	-4.450058	1.197702	0.239303	C	-4.66665	1.197072	0.168044
C	-5.717763	-1.202033	0.812468	C	-5.95529	-1.20179	0.693765
H	-3.957863	-2.140803	0.013446	H	-4.16502	-2.13986	-0.0375
C	-5.717834	1.201677	0.812453	C	-5.95536	1.201418	0.693749
H	-3.957989	2.140541	0.013421	H	-4.16515	2.139586	-0.03753
C	-6.358132	-0.000196	1.103419	C	-6.60704	-0.0002	0.960554
H	-6.209013	-2.146274	1.028667	H	-6.45472	-2.14609	0.891895
H	-6.209139	2.145891	1.028642	H	-6.45485	2.145691	0.891867
H	-7.347727	-0.000222	1.549482	H	-7.61324	-0.00023	1.368551

(e) **(f)**

Symbol	X	Y	Z	Symbol	X	Y	Z
C	2.35059	-1.223379	0.884341	C	2.12932	-1.21092	1.003283
C	2.358796	1.209919	0.886673	C	2.128215	1.210978	1.003424
C	2.928737	1.19442	2.149416	C	2.43566	1.203356	2.359244
C	3.233625	-0.011752	2.764866	C	2.617059	0.000001	3.012225
C	2.922213	-1.214639	2.146313	C	2.437425	-1.20352	2.359125
H	3.109741	2.123029	2.671809	H	2.531892	2.134155	2.898048
H	3.697826	-0.014214	3.744734	H	2.86869	0.000149	4.068445
H	3.097373	-2.145537	2.666599	H	2.534647	-2.1343	2.897767

O	1.032529	-1.165364	-1.593783	O	1.77833	-1.17224	-1.76273
O	1.041347	1.164692	-1.592398	O	1.778661	1.172495	-1.76289
C	2.0356	-2.436104	0.15945	C	1.917463	-2.43962	0.244248
C	1.434535	-2.353007	-1.105108	C	1.786288	-2.3854	-1.14362
C	2.358333	-3.727592	0.639845	C	1.849435	-3.716	0.84881
C	1.180864	-3.49116	-1.874338	C	1.604491	-3.52306	-1.92192
C	2.106612	-4.850757	-0.110014	C	1.672843	-4.8584	0.087564
H	2.807239	-3.841515	1.619091	H	1.899476	-3.80896	1.926555
C	1.52017	-4.732773	-1.385128	C	1.552974	-4.76172	-1.30795
H	0.707689	-3.359124	-2.840852	H	1.487023	-3.4065	-2.9931
H	2.360892	-5.829751	0.280581	H	1.607681	-5.82682	0.57129
H	1.331435	-5.620683	-1.978724	H	1.404601	-5.65595	-1.90441
C	2.054132	2.426134	0.162369	C	1.91639	2.439756	0.244201
C	2.387385	3.715068	0.642819	C	1.847613	3.716288	0.848456
C	1.453083	2.348688	-1.102751	C	1.786405	2.385789	-1.14393
C	2.14593	4.840326	-0.107472	C	1.671636	4.858572	0.086845
H	2.836657	3.825402	1.62228	H	1.896718	3.809496	1.926215
C	1.209691	3.488932	-1.872283	C	1.605114	3.523333	-1.92259
C	1.559488	4.727564	-1.383088	C	1.553	4.761978	-1.30878
H	2.408402	5.817144	0.283132	H	1.605984	5.827025	0.570416
H	0.736134	3.360812	-2.839164	H	1.488501	3.406604	-2.99386
H	1.378965	5.616987	-1.976999	H	1.40508	5.656178	-1.90541
C	-1.920041	0.006426	0.558499	C	-1.8299	-0.00024	0.506616
H	-1.901504	0.91205	1.16648	H	-1.85103	0.902153	1.118471
H	-1.906148	-0.899479	1.166203	H	-1.85109	-0.90281	1.118215
C	-2.919161	0.008299	-0.571166	C	-2.78046	-0.0003	-0.67208
H	-2.766568	-0.869457	-1.211697	H	-2.59989	-0.88026	-1.30496
H	-2.76324	0.88494	-1.212401	H	-2.60004	0.879542	-1.30516
B	1.246361	-0.001506	-0.932755	B	1.831106	0.000138	-1.10492
N	2.192021	-0.00559	0.219463	N	2.114206	0.000102	0.342475
C	-4.361432	0.010805	-0.094009	C	-4.2414	-0.00035	-0.26584
C	-5.041689	-1.185765	0.149058	C	-4.93467	-1.19665	-0.04993
C	-5.03804	1.209769	0.147164	C	-4.93315	1.196182	-0.04545
C	-6.350775	-1.18744	0.620893	C	-6.25767	-1.20127	0.377036
H	-4.533975	-2.129271	-0.035402	H	-4.41918	-2.14079	-0.21835
C	-6.347199	1.216096	0.618876	C	-6.25603	1.200992	0.381349
H	-4.527519	2.151434	-0.038895	H	-4.4163	2.140191	-0.21041
C	-7.010738	0.015508	0.859688	C	-6.92846	-0.00017	0.597841

H	-6.859176	-2.130799	0.79898	H	-6.77032	-2.14763	0.533617
H	-6.852848	2.161228	0.79532	H	-6.76755	2.147389	0.541353
H	-8.032589	0.017308	1.226422	H	-7.96344	-3E-06	0.928437

■ Giese addition

Table S2. Optimization of reaction conditions for Giese addition of **P3a** with dimethyl fumarate **2A**.

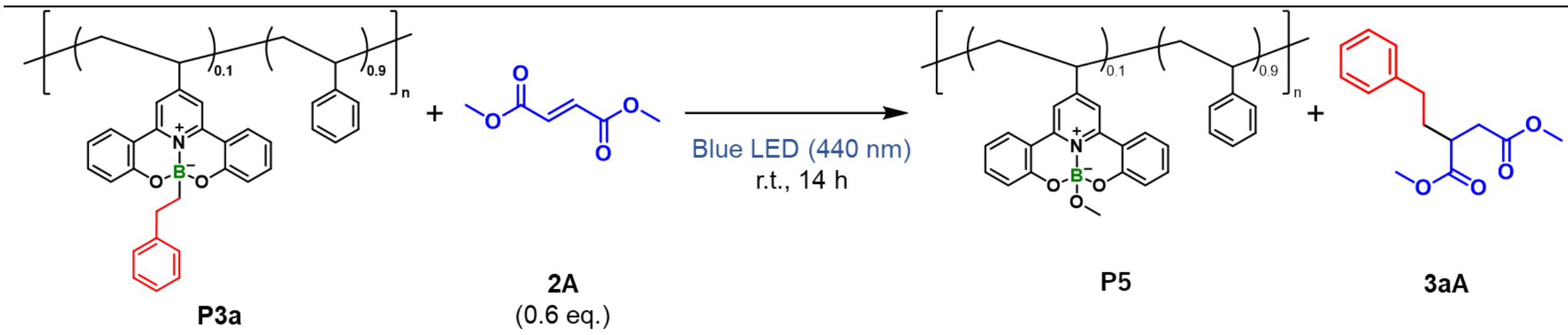

Entry	Solvent	Concentration of **P3a** (mM)	Yield of **3aA** (%)[a]
1	DCE	60	30
2	DCE/MeOH (9:1)	60	43
3	DCE/MeOH (9:1)	3	46

[a]Determined by ^{1}H NMR using nitromethane as the internal standard.

To a 20 mL vial were added **P3a**, **2A**, and DCE/MeOH (9:1). The vial was sealed with a rubber septum and bubbled with Ar for 10 min. The mixture was stirred at room temperature for 14 h under irradiation with 440 nm light. The solvent was removed under reduced pressure, and the residue was dried under vacuum to afford the crude product. The ^{1}H NMR signals of product **3aA** were consistent with those reported in the literature.[34]

P5: yellow solid; ^{1}H NMR (500.13 MHz, CD_2Cl_2, ppm): δ = 7.46 (br), 7.06 (br), 6.59 (br), 3.09 (br), 1.80 (br), 1.47 (br); ^{13}C NMR (125.76 MHz, CD_2Cl_2, ppm): δ = 157.1, 147.8, 145.7, 134.0, 128.4, 128.0, 126.1, 120.6, 120.1, 49.1, 46.2, 44.5, 40.8; ^{11}B NMR (128.38 MHz, CD_2Cl_2, ppm): δ = 2.12. M_n = 19,300, M_w = 65,900, $Đ$ = 3.4 (GPC relative to polystyrene standards); elemental analysis (%): calcd.: C 87.10, H 6.98, N 1.14, O 3.90, B 0.88, found: C 86.61, H 7.12, N 1.70. B prevented detection of O.

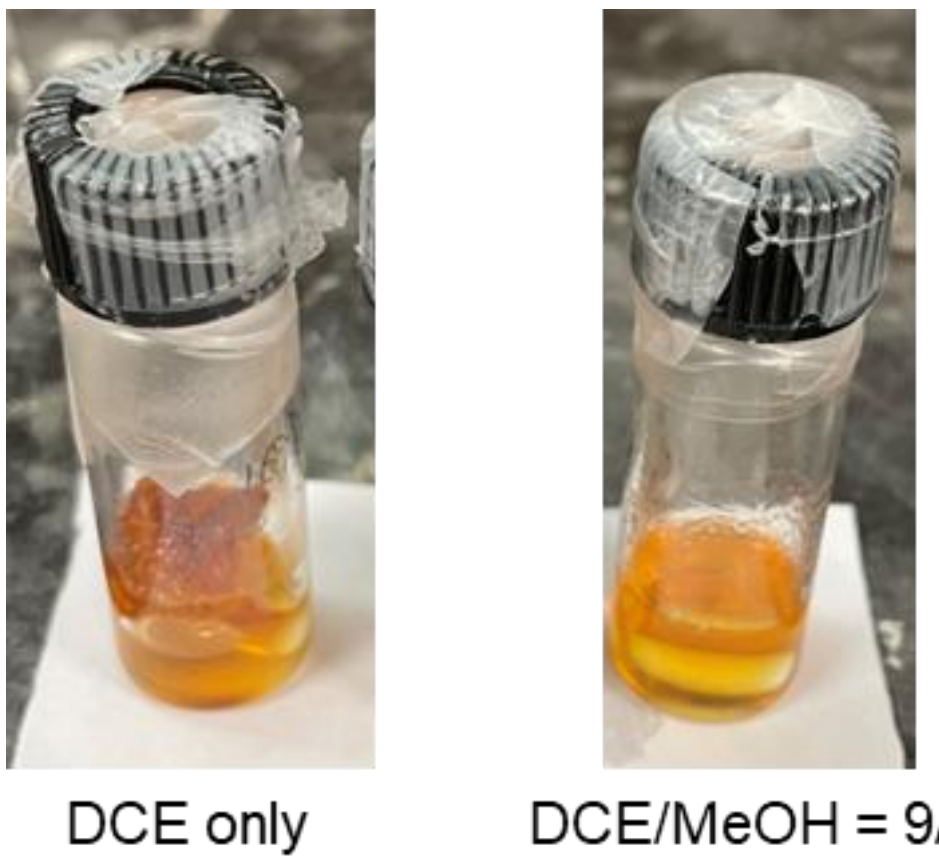

Figure S6. Photographs of the solutions after light irradiation (entries 1 and 2 of Table S2).

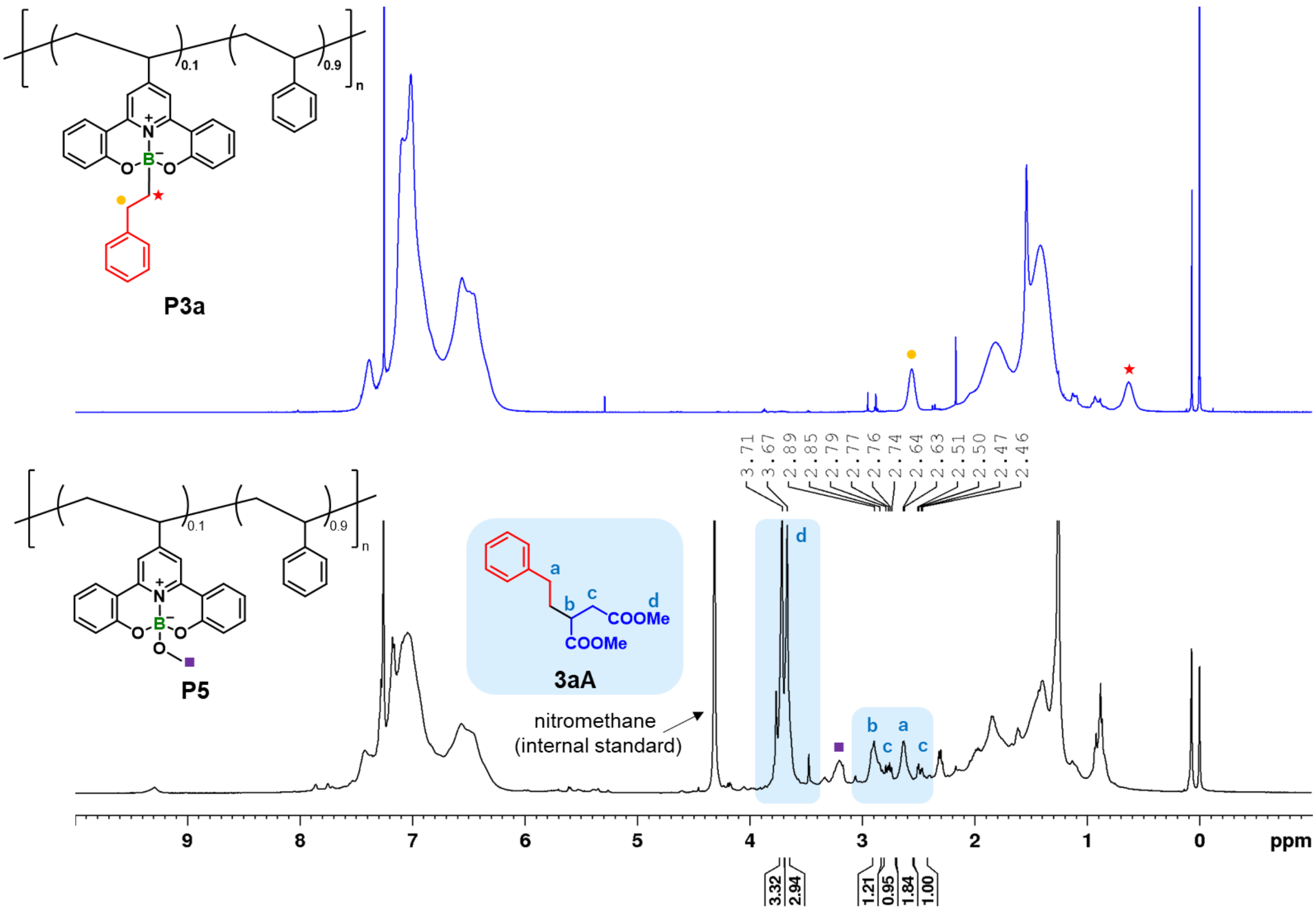

Figure S7. ^{1}H NMR spectra (500.13 MHz, $CDCl_3$) of **P3a** and the crude product after Giese addition reaction using radical acceptor **2A** in entry 3 of Table S2.

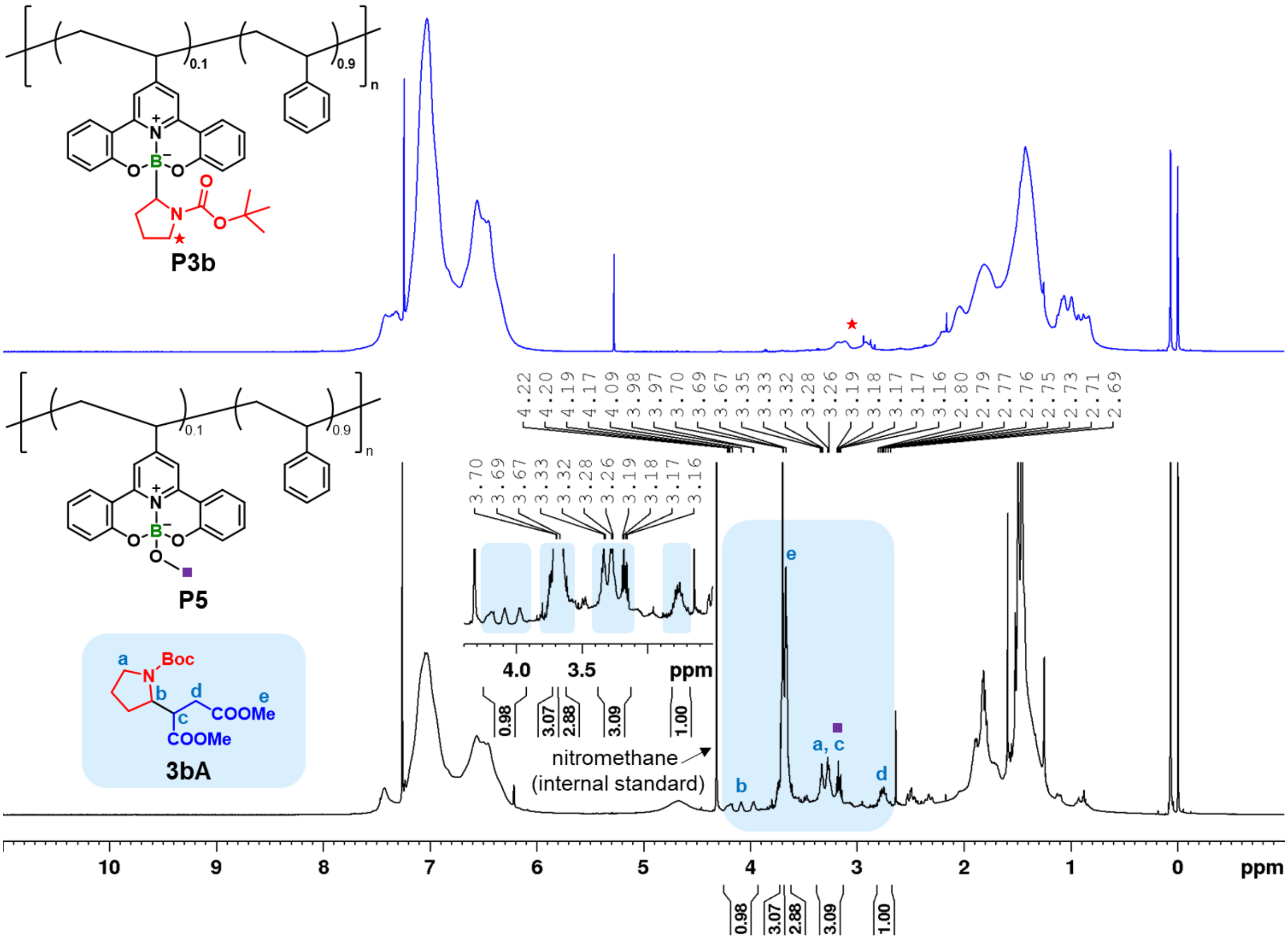

Figure S8. ^{1}H NMR spectra (500.13 MHz, $CDCl_3$) of **P3b** and the crude product after Giese addition reaction using radical acceptor **2A**.

Detailed experimental conditions for Figure 3

3aA: **P3a** (77.7 mg, 0.0474 mmol, 1.5 eq), **2A** (4.52 mg, 0.0313 mmol, 1.0 eq), and DCE/MeOH (9:1, 11 mL) were used to afford the crude product in 46% NMR yield.

3aB: **P3a** (54.61 mg, 0.0333 mmol, 1.5 eq), **2B** (3.63 mg, 0.0224 mmol, 1.0 eq), and DCE/MeOH (9:1, 7 mL) were used to afford the crude product in 24% NMR yield.

3aC: **P3a** (49.86 mg, 0.0304 mmol, 1.5 eq), **2C** (3.07 mg, 0.0199 mmol, 1.0 eq), and DCE/MeOH (9:1, 7 mL) were used to afford the crude product in 84% NMR yield.

3bA: **P3b** (53.80 mg, 0.0268 mmol, 1.5 eq), **2A** (2.60 mg, 0.0180 mmol, 1.0 eq), and DCE/MeOH (9:1, 6 mL) were used to afford the crude product in 94% NMR yield.

3bB: **P3b** (75.50 mg, 0.0374 mmol, 1.5 eq), **2B** (4.11 mg, 0.0253 mmol, 1.0 eq), and DCE/MeOH (9:1, 9 mL) were used to afford the crude product in >99% NMR yield.

3bC: **P3b** (70.88 mg, 0.0351 mmol, 1.4 eq), **2C** (3.89 mg, 0.0252 mmol, 1.0 eq), and DCE/MeOH (9:1, 9 mL) were used to afford the crude product in 39% NMR yield.

The ^{1}H NMR signals of the products were consistent with those reported in the literatures.[34–38]

■ Recycle use

Recovery of PDP polymer from P5

Table S3. Investigation of conditions for recovery of **P2** from **P5** using fluoride sources.

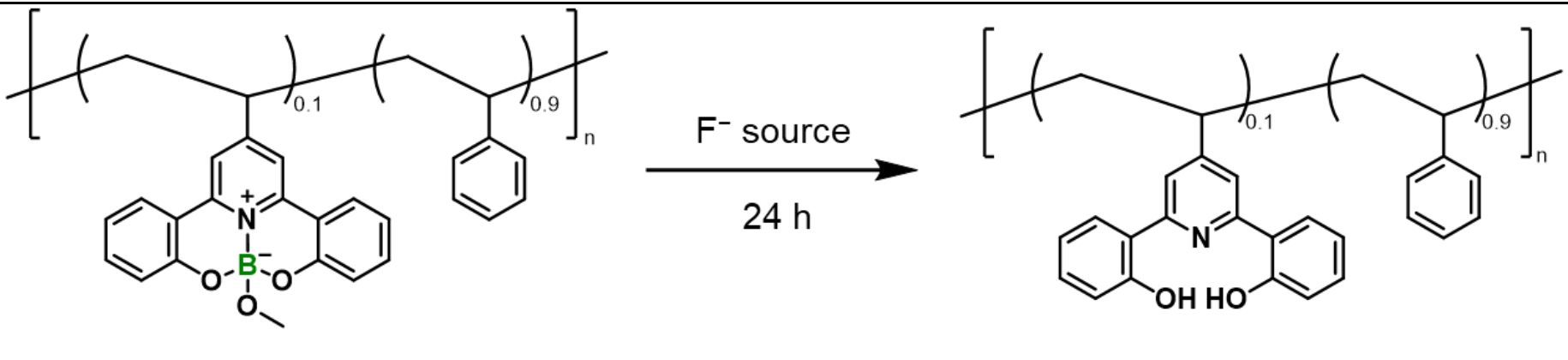

Entry	F^- source	Solvent	Temperature	Recovery rate of PDP moiety in **Re-P2** (%)[a]
1	TBAF	Nitrobenzene	130 °C	26
2	Et_3N-3HF	Nitrobenzene	r.t.	–[b]
3	CsF	Diglyme	130 °C	22
4	TBAF	Diglyme	130 °C	48
5	TBAF[c]	Diglyme	130 °C	26

[a]Determined by ^{19}F NMR using nitromethane as the internal standard after triflation of **Re-P2**.
[b]Not investigated because no significant change was observed in the ^1H NMR spectrum of the crude product.
[c]Prepared using dry Molecular Sieves 3A.

To a Schlenk tube were added TBAF (1.0 M in THF, 3.0 mL, 3.0 mmol, 112 eq) and diglyme (1 mL). THF was removed using a rotary evaporator, and then **P5** (33.08 mg, 0.0269 mmol, 1.0 eq) was added. The mixture was stirred at 130 °C for 24 h. After cooling to room temperature, the mixture was reprecipitated using methanol as a poor solvent. After decantation, the precipitate was collected by filtration to afford a yellow solid (23.38 mg).

Re-P2: M_n = 10,600, M_w = 35,300, $Đ$ = 3.3 (GPC relative to polystyrene standards).

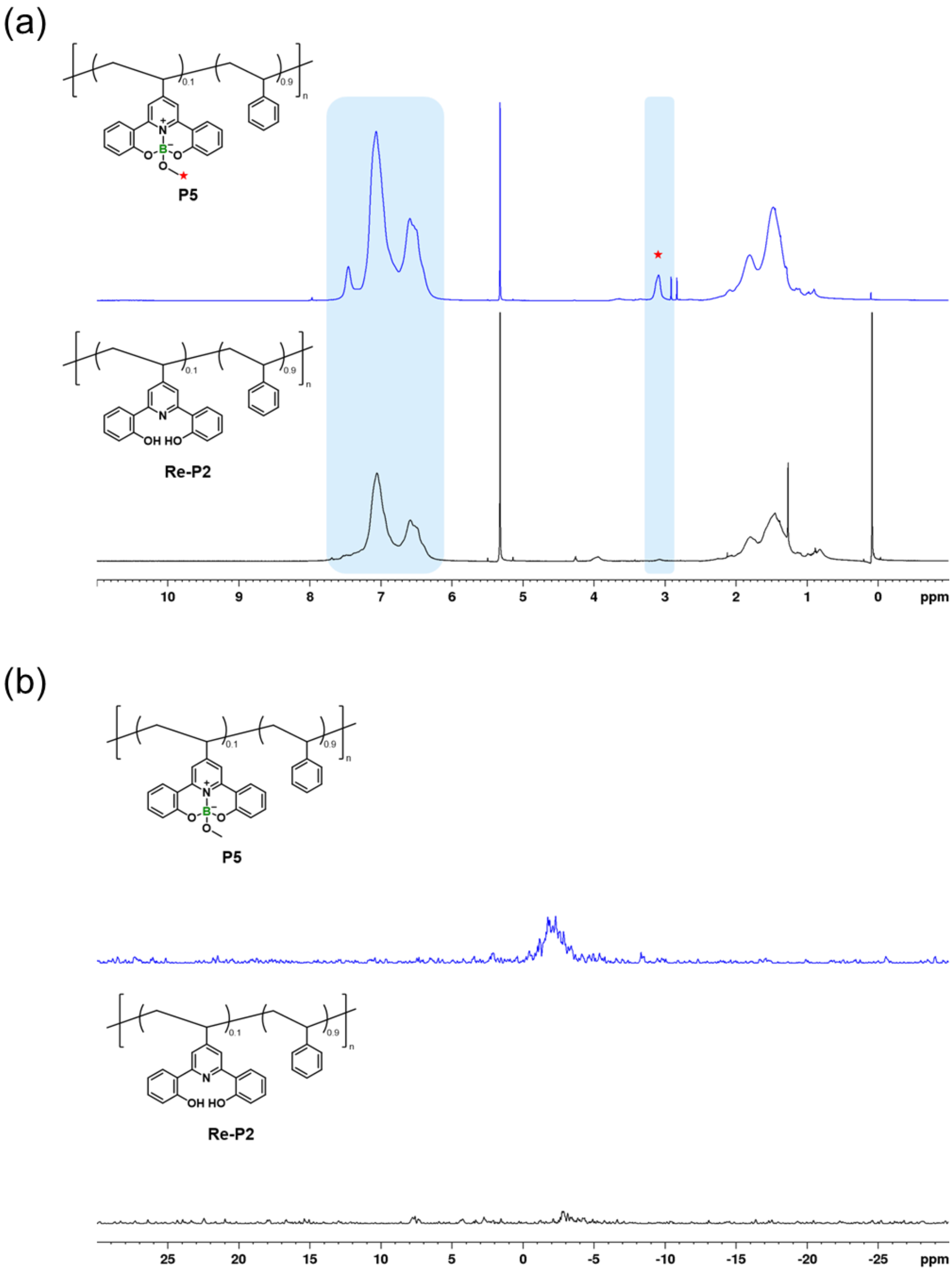

Figure S9. (a) ^{1}H NMR spectra (500.13 MHz, CD_2Cl_2) of **P5** and **Re-P2**. (b) ^{11}B NMR spectra (128.38 MHz, $CDCl_3$) of **P5** and **Re-P2**.

Triflation of P2

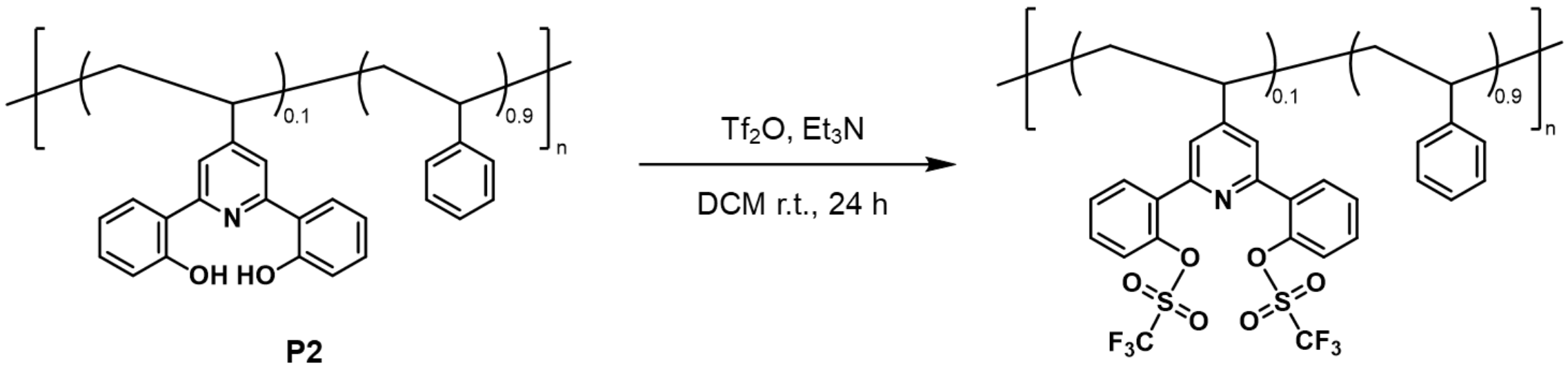

Scheme S7. Triflation of **P2**. Quantitative triflation was confirmed by ^{19}F NMR using benzotrifluoride as the internal standard.

To a 5 mL vial were added **P2** (22.68 mg, 0.020 mmol, 1.0 eq), Et_3N (0.011 mL, 0.079 mmol, 4.0 eq), and DCM (1 mL). The mixture was cooled to 0 °C, and Tf_2O (0.020 mL, 0.12 mmol, 6.0 eq) was added dropwise. The mixture was stirred at room temperature for 24 h. Distilled water was added, and the mixture was extracted with DCM, washed with brine, and dried over anhydrous Na_2SO_4. The solvent was removed under reduced pressure, and the residue was dried under vacuum to afford a yellow solid (10.9 mg, 0.0078 mmol, 39%).

P2OTf: yellow solid; ^{1}H NMR (500.13 MHz, CD_2Cl_2, ppm): δ = 7.64 (br), 7.49 (br), 7.38 (br), 7.05 (br), 6.80 (br), 6.58 (br), 1.81 (br), 1.47 (br); ^{13}C NMR (125.76 MHz, CD_2Cl_2, ppm): δ = 154.0, 147.4, 145.7, 134.3, 132.7, 130.5, 128.9, 128.4, 128.2, 128.0, 126.1, 123.3, 122.4, 120.1, 117.5, 44.5, 40.8; ^{19}F NMR (470.59 MHz, CD_2Cl_2, ppm): δ = −73.60, −73.66, −162.20. M_n = 19,700, M_w = 73,600, $Đ$ = 3.7 (GPC relative to polystyrene standards); elemental analysis (%): calcd.: C 74.50, H 5.70, N 0.96, O 6.60, F 7.83, S 4.41, found: C 80.04, H 6.52, N 1.52, F 4.51, S 2.25. F prevented detection of O.

Re-complexation of Re-P2 with 2-phenylethylboronic acid

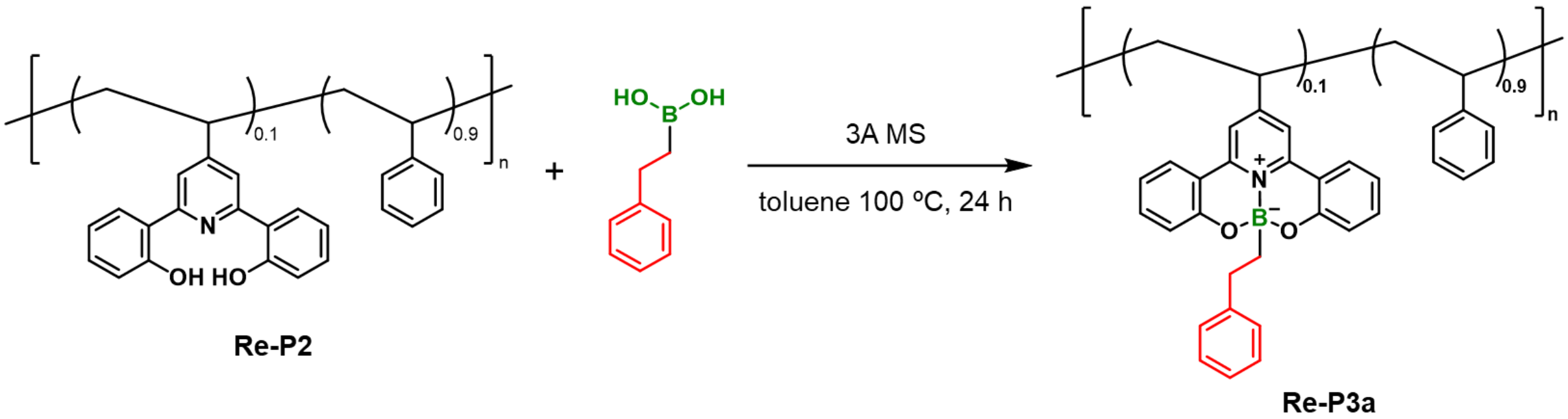

Scheme S8. Borate complex formation of **Re-P2** with 2-phenylethylboronic acid.

Re-P3a: M_n = 11,100, M_w = 32,000, $Đ$ = 2.9 (GPC relative to polystyrene standards).

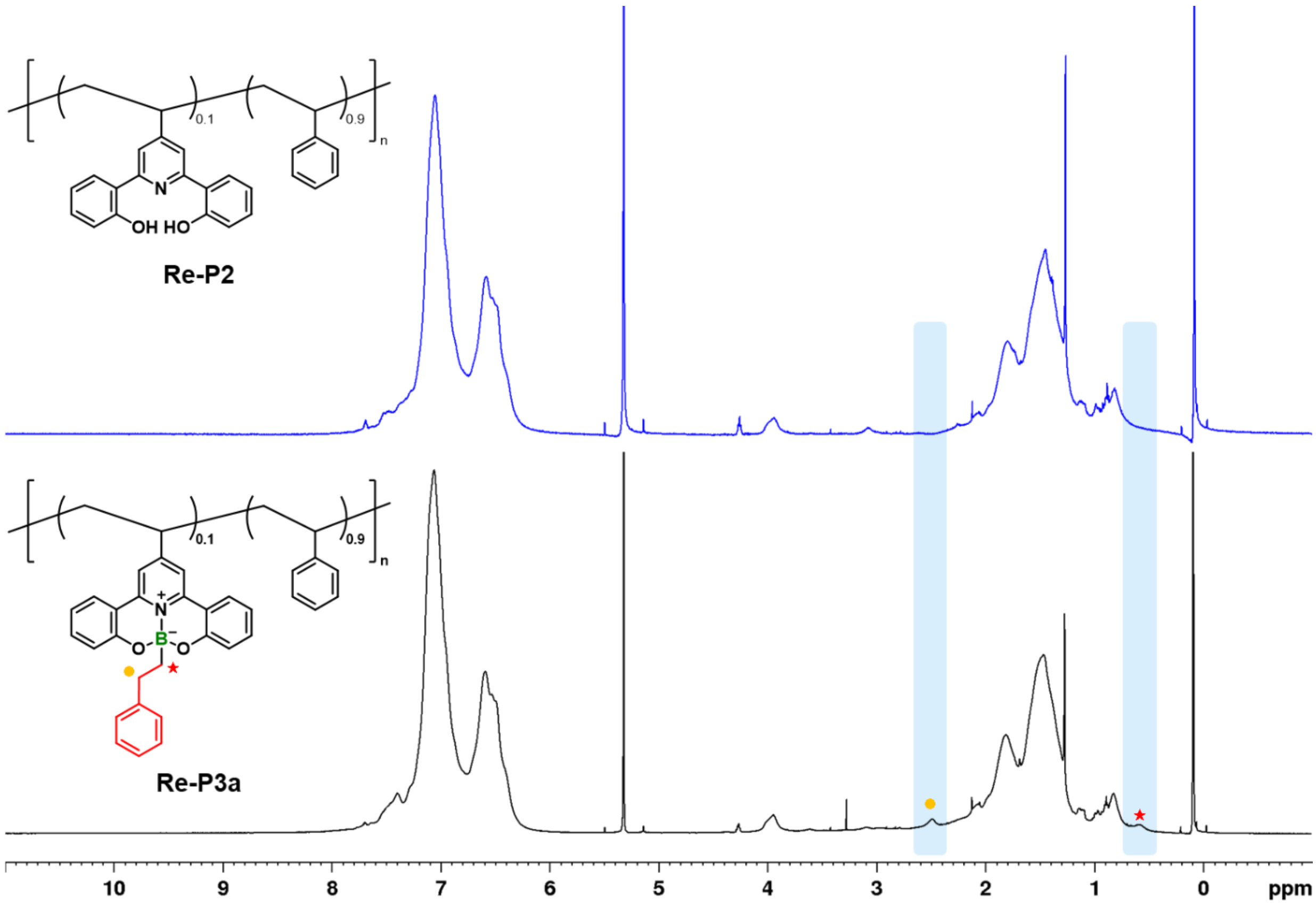

Figure S10. ^{1}H NMR spectra (500.13 MHz, CD_2Cl_2) of **Pe-P2** and **Re-P3a**.

A signal at approximately 3.8 ppm is observed in the ^{1}H NMR spectra of **Re-P2** and **Re-P3a** but is absent in **P5**. The signal intensity remains comparable between **Re-P2** and **Re-P3a**, suggesting that it originates from a species formed during the TBAF treatment step rather than the re-complexation step. While this signal could not be assigned, its broad nature suggests a polymeric origin, possibly arising from a side reaction during fluoride-mediated cleavage, which may contribute to the incomplete PDP recovery.

■ **GPC profiles**

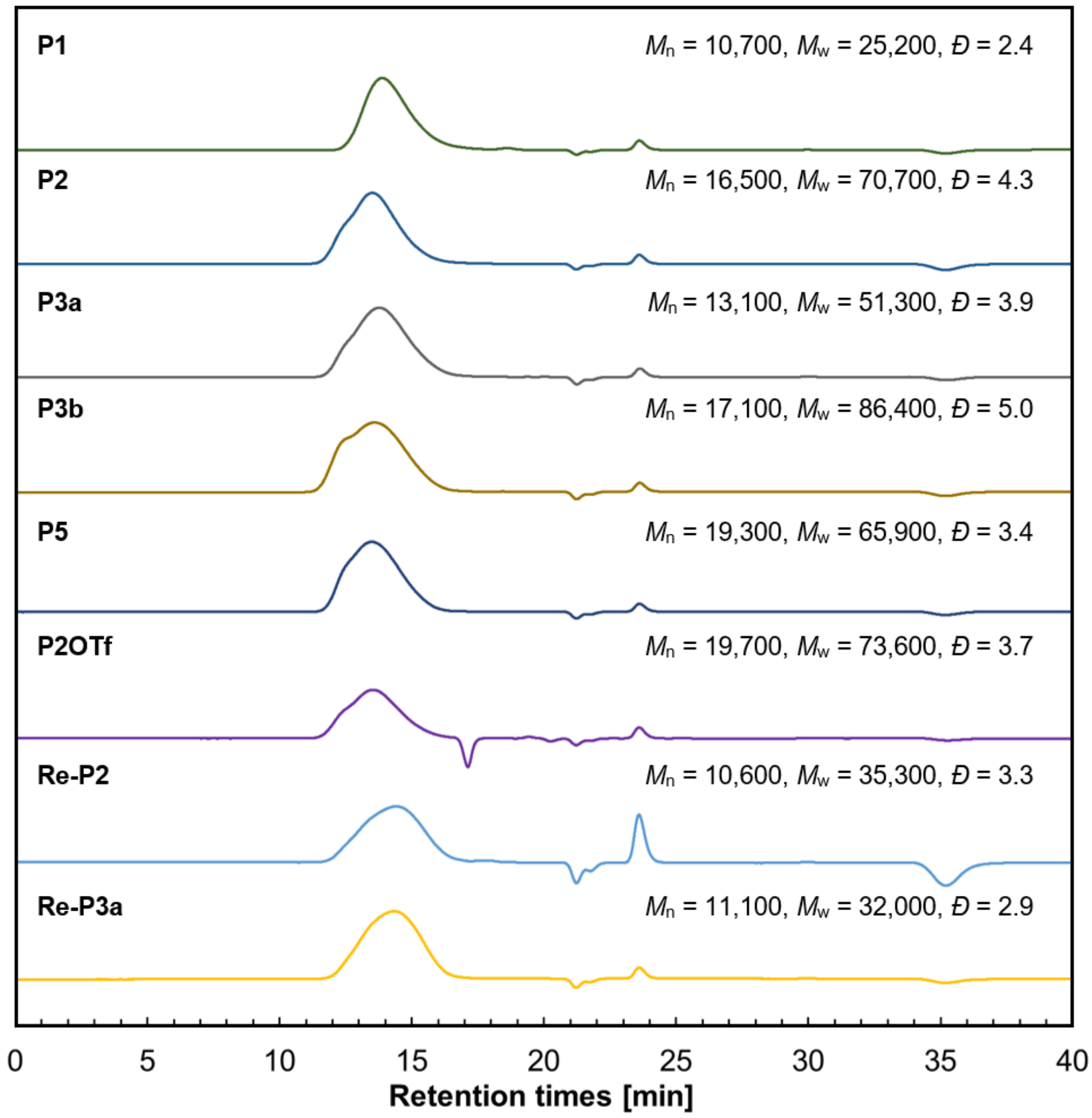

Figure S11. GPC profies of each polymer.

■ Network polymer formation

Synthesis of bistrifluoroborate (4)

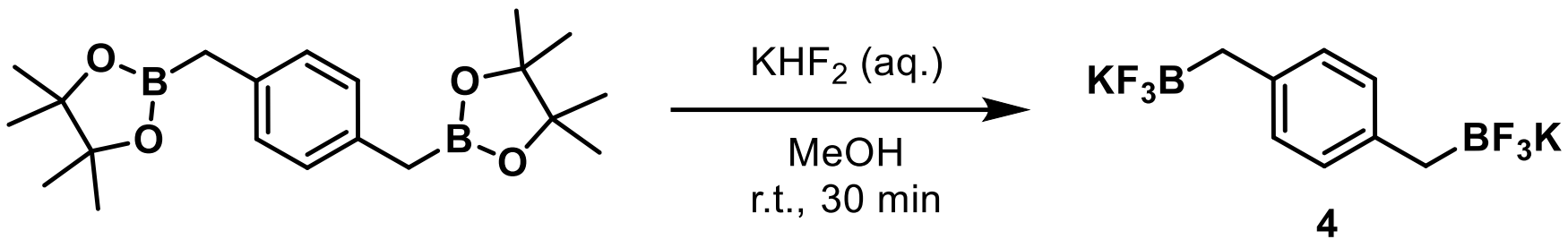

Scheme S9. Synthesis of **4**.

1,4-Bis((4,4,5,5-tetramethyl-1,3,2-dioxaborolan-2-yl)methyl)benzene was synthesized according to the literature.[39] Following a literature procedure,[40] 1,4-bis((4,4,5,5-tetramethyl-1,3,2-dioxaborolan-2-yl)methyl)benzene (511.8 mg, 1.43 mmol, 1.0 eq) was added to a 50 mL round-bottom flask and dissolved in methanol (40 mL). KHF_2 (4.5 M in water, 1.27 mL, 5.72 mmol, 4.0 eq) was added dropwise, and the mixture was stirred at room temperature for 30 h. After washing with Et_2O, the mixture was filtered using DMF. The solvent was removed under reduced pressure to afford a white solid (433.3 mg, 1.36 mmol, 90%).

4: white solid; 5% and 10% weight loss temperatures ($T_{5\%}$, $T_{10\%}$); $T_{5\%}$: 236 °C, $T_{10\%}$: 253 °C ^{1}H NMR (500.13 MHz, DMSO-d_6, ppm): 6.67 (s, 4H, Ar*H*), 1.31 (br, 4H, CH_2); ^{13}C NMR (125.76 MHz, DMSO-d_6, ppm): δ = 139.57, 127.47; ^{11}B NMR (160.46 MHz, DMSO-d_6, ppm): δ = 4.2 (br); ^{19}F NMR (470.59 MHz, DMSO-d_6, ppm): δ = −136.17; HRMS (ESI) calcd for $C_8H_8B_2F_6K$ ($[M+H]^+$) m/z 279.0359, found 279.0357.

Crosslinking of P2 with 4 through borate formation

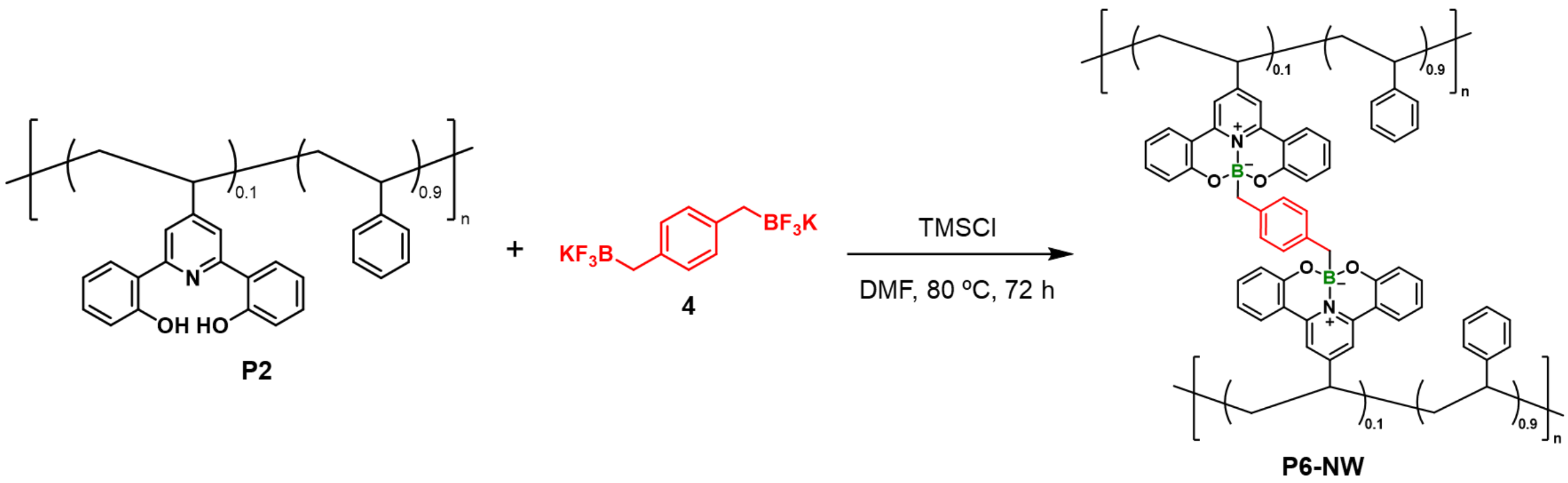

Scheme S10. Synthesis of **P6-NW**.

To a Schlenk tube were added **P2** (114.22 mg, 0.101 mmol, 1.0 eq) and **4** (15.98 mg, 0.0503 mmol, 0.5 eq), and the mixture was dissolved in DMF (1 mL). TMSCl (0.092 mL, 0.72 mmol, 7.2 eq) was added dropwise, and the mixture was heated at 80 °C for 72 h. After cooling to room temperature, the mixture was washed with DMF and DCM to afford an insoluble gel product (128.15 mg).

P6-NW: elemental analysis (%): calcd.: C 88.52, H 6.94, N 1.12, O 2.56, B 0.86, found: C 81.23, H 6.85, N 2.7.

Photo-degradation of P6-NW in the presence of TEMPO

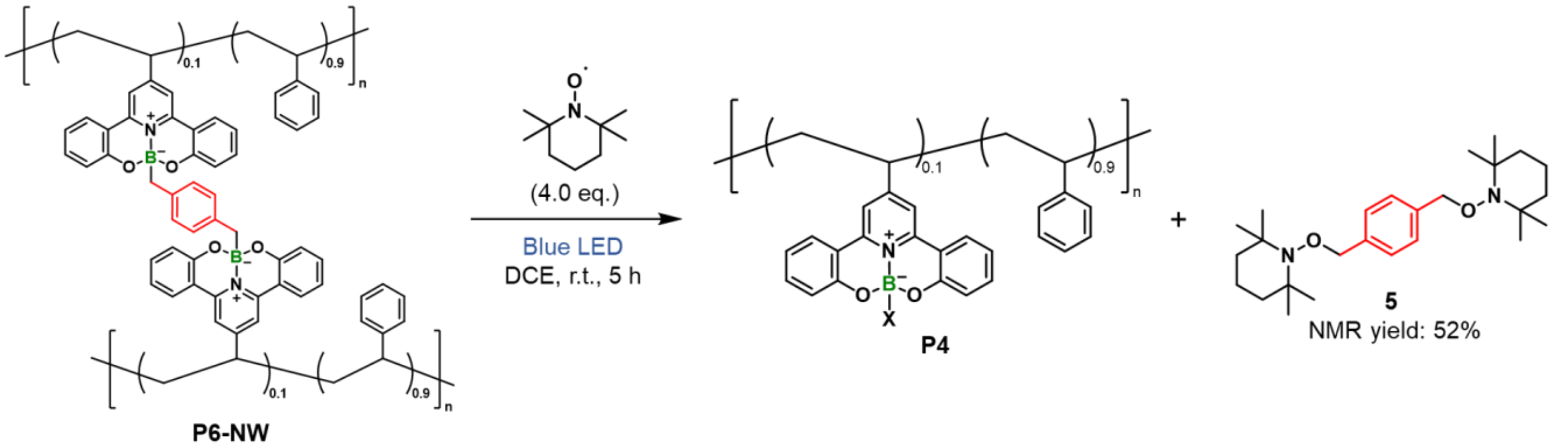

Scheme S11. Synthesis of **P6-NW**. Yield of **5** was determined by ^{1}H NMR using 1,1,2,2-tetrachloroethane as the internal standard. B–X in **P4** is an unidentified boron-residue.

To a 5 mL vial were added **P6-NW** (40.23 mg, 0.017 mmol, 1.0 eq), TEMPO (10.30 mg, 0.066 mmol, 3.9 eq), and DCE (0.5 mL). The mixture was degassed with Ar for 5 min. The mixture was then stirred at room temperature for 5 h under irradiation with 440 nm light. After the reaction, the mixture was dissolved in DCM and reprecipitated using methanol as a poor solvent to afford a yellow powder and the filtrate was collected. The ^{1}H NMR signals of **5** were consistent with those reported in the literature.[41]

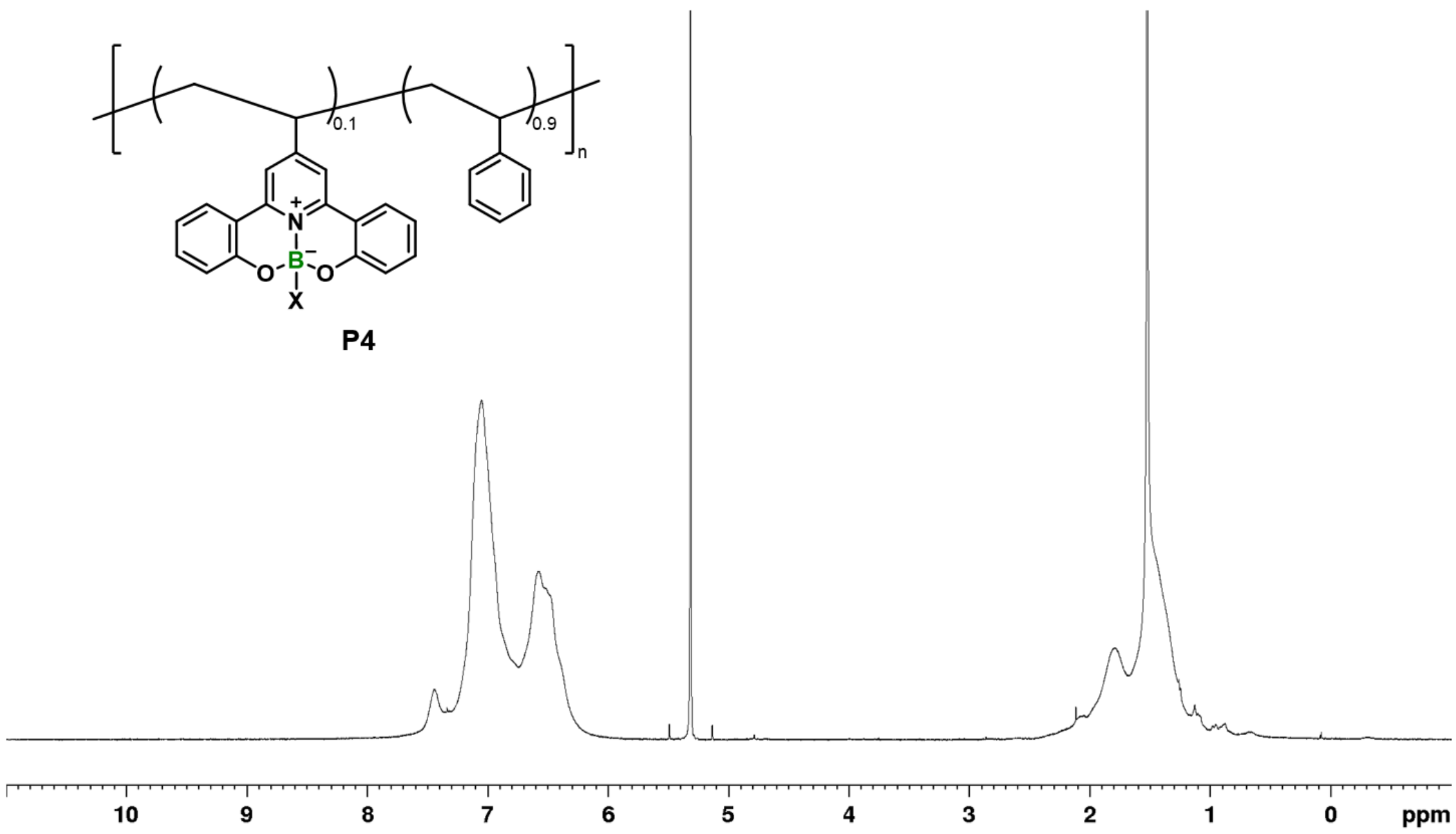

Figure S12. ^{1}H NMR spectra (500.13 MHz, CD_2Cl_2) of **P4** obtained in **Scheme S11**.

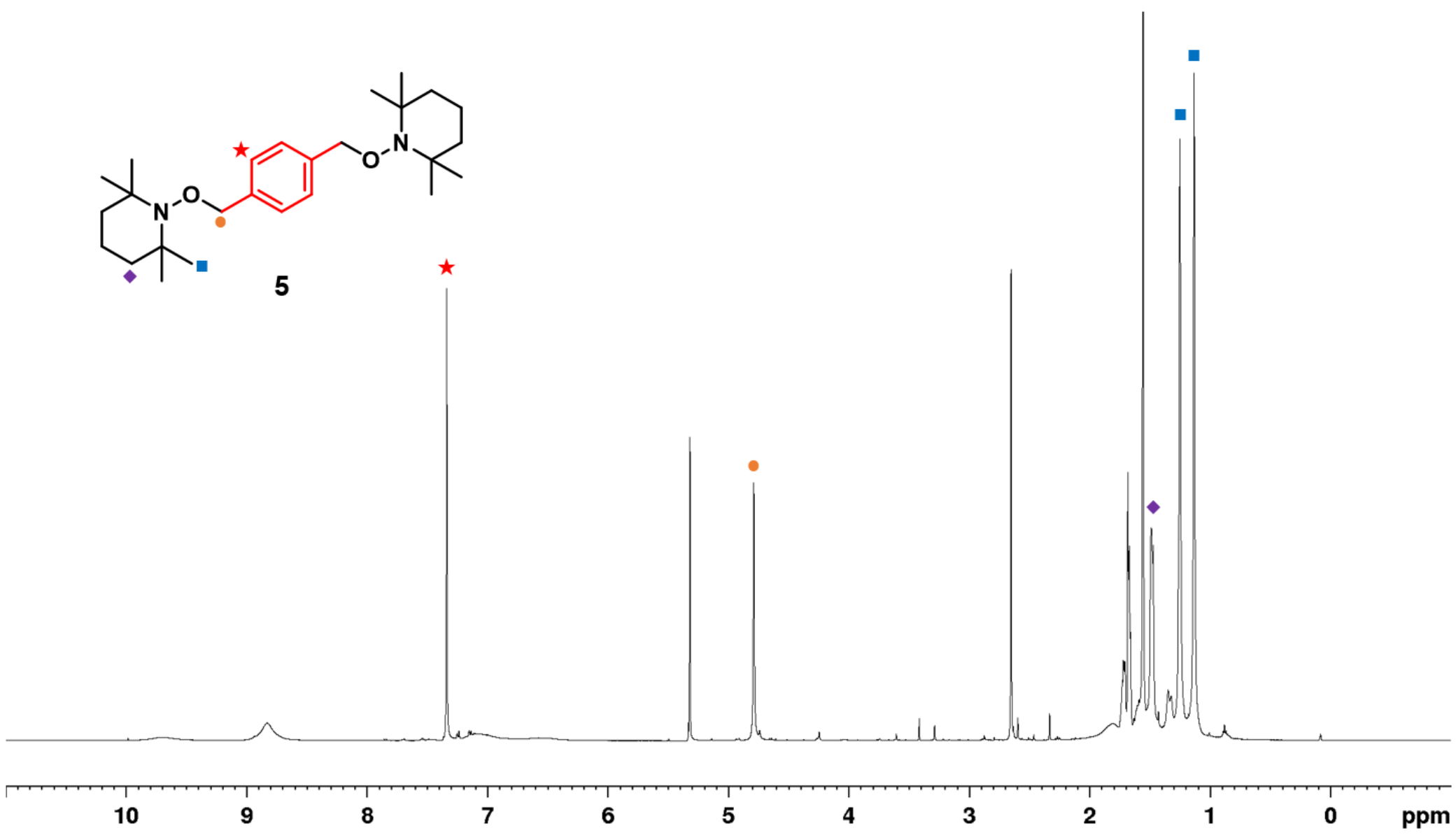

Figure S13. ^{1}H NMR spectra (500.13 MHz, CD_2Cl_2) of crude **5**.

■ **NMR spectra**

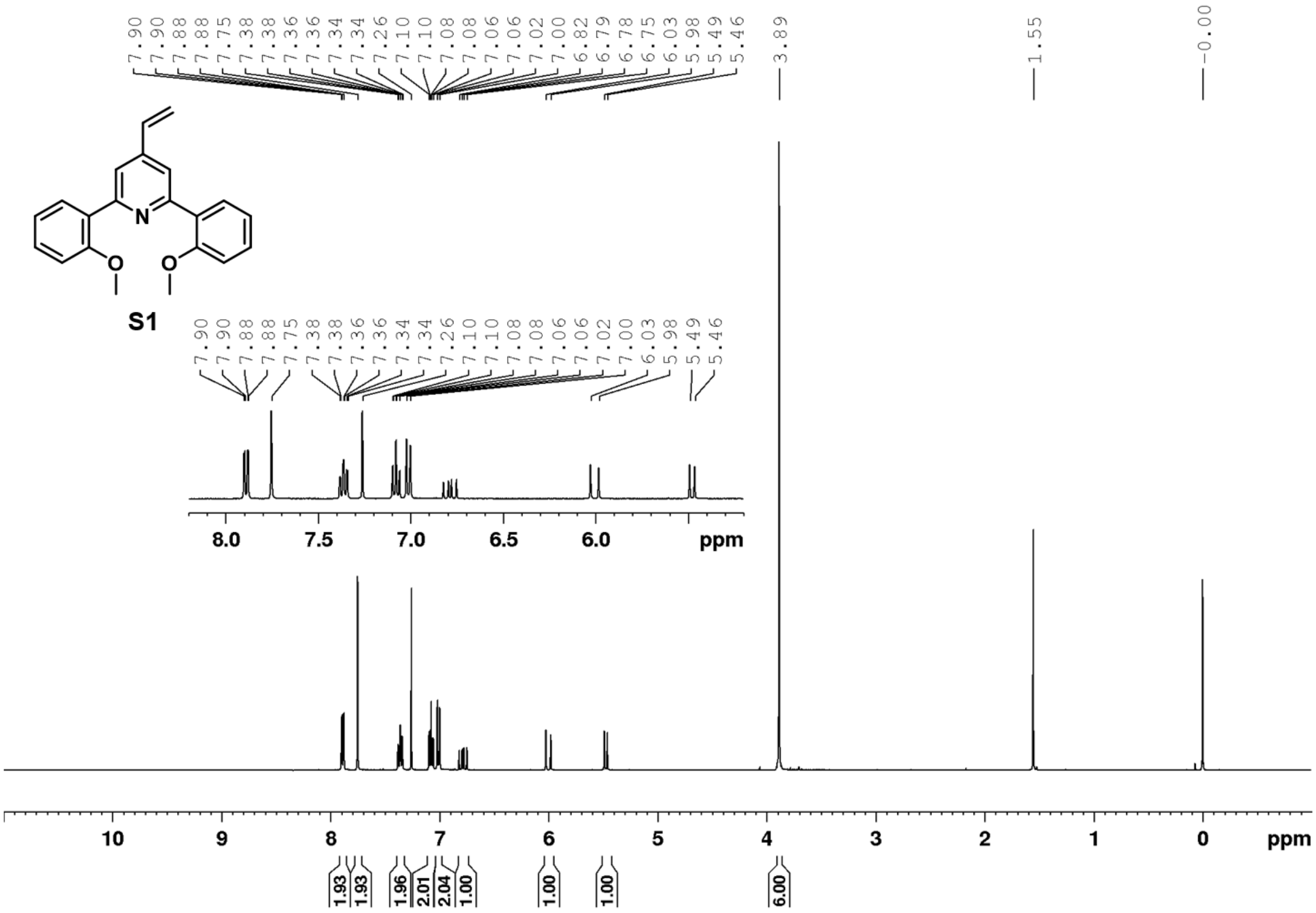

^{1}H NMR spectrum (500.13 MHz, $CDCl_3$) of **S1**

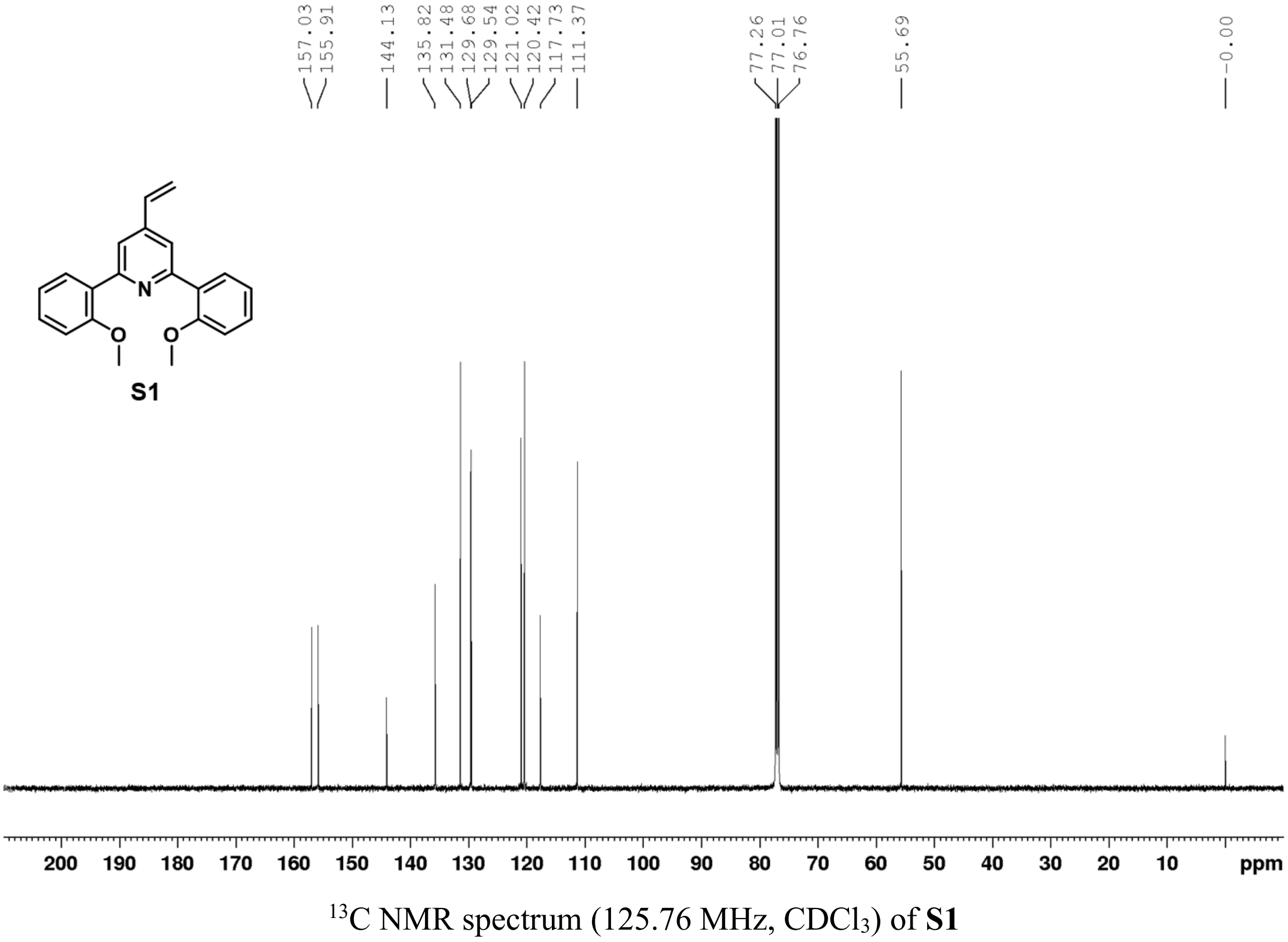

^{13}C NMR spectrum (125.76 MHz, $CDCl_3$) of **S1**

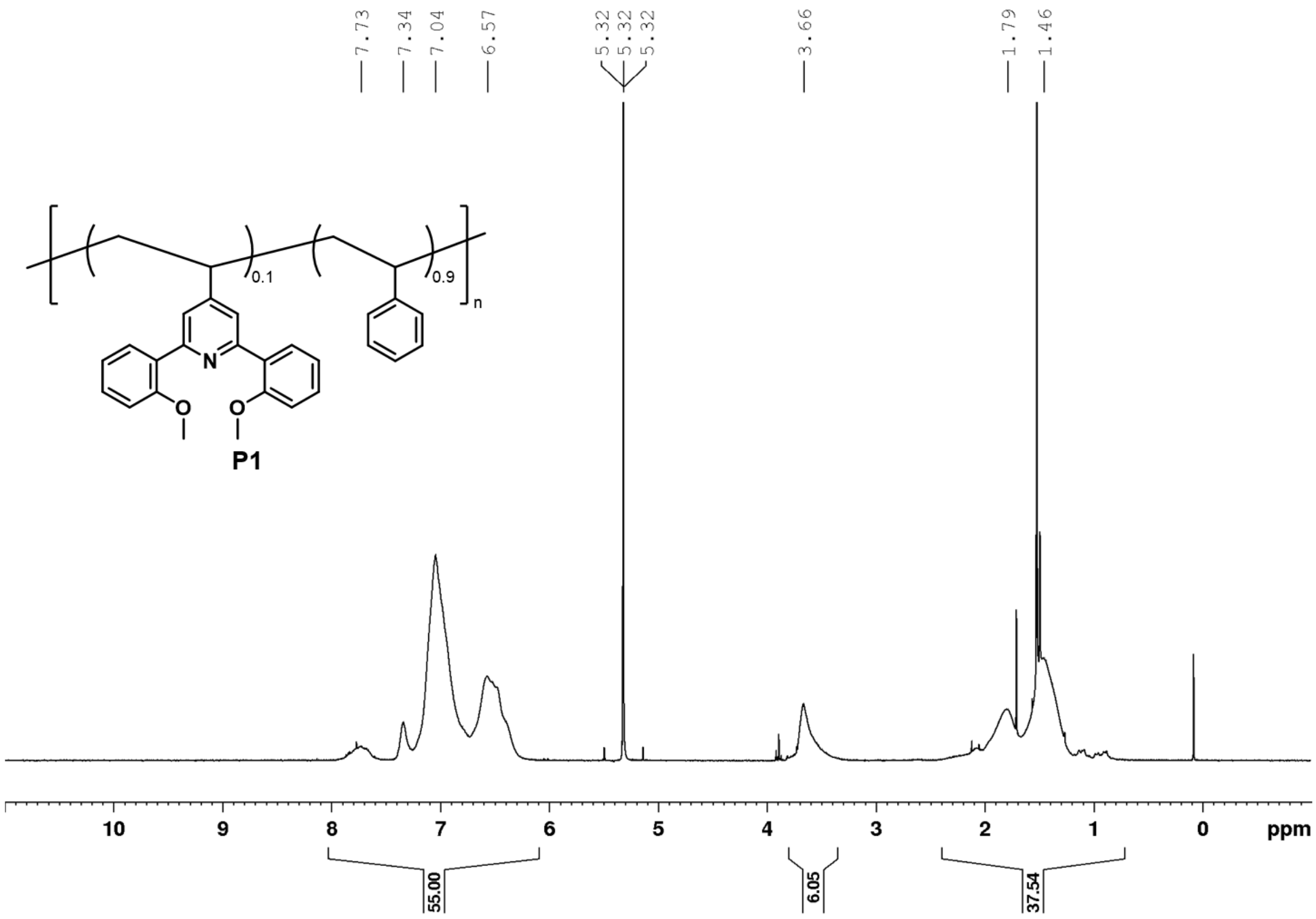

^{1}H NMR spectrum (500.13 MHz, CD_2Cl_2) of **P1**

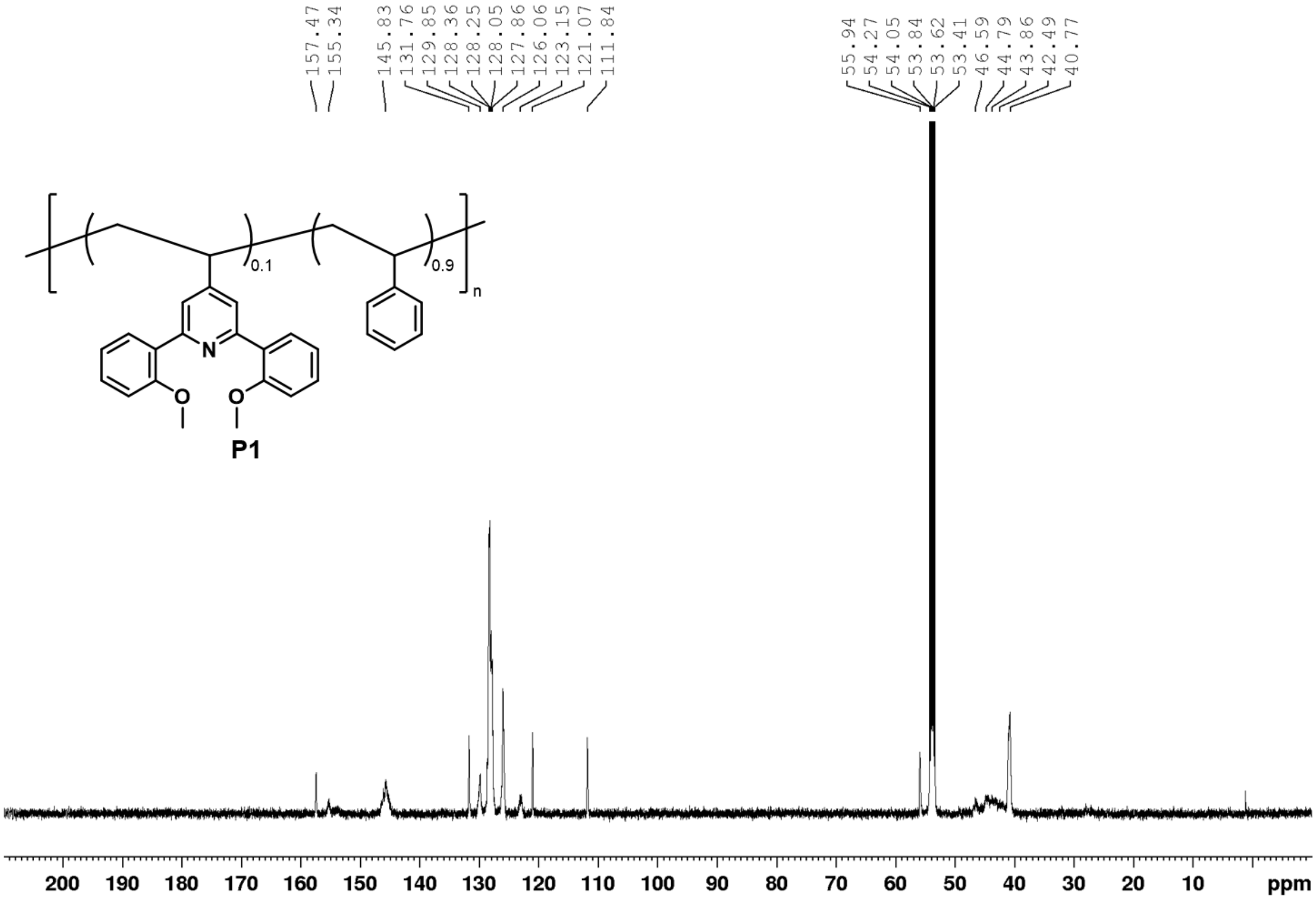

^{13}C NMR spectrum (125.76 MHz, CD_2Cl_2) of **P1**

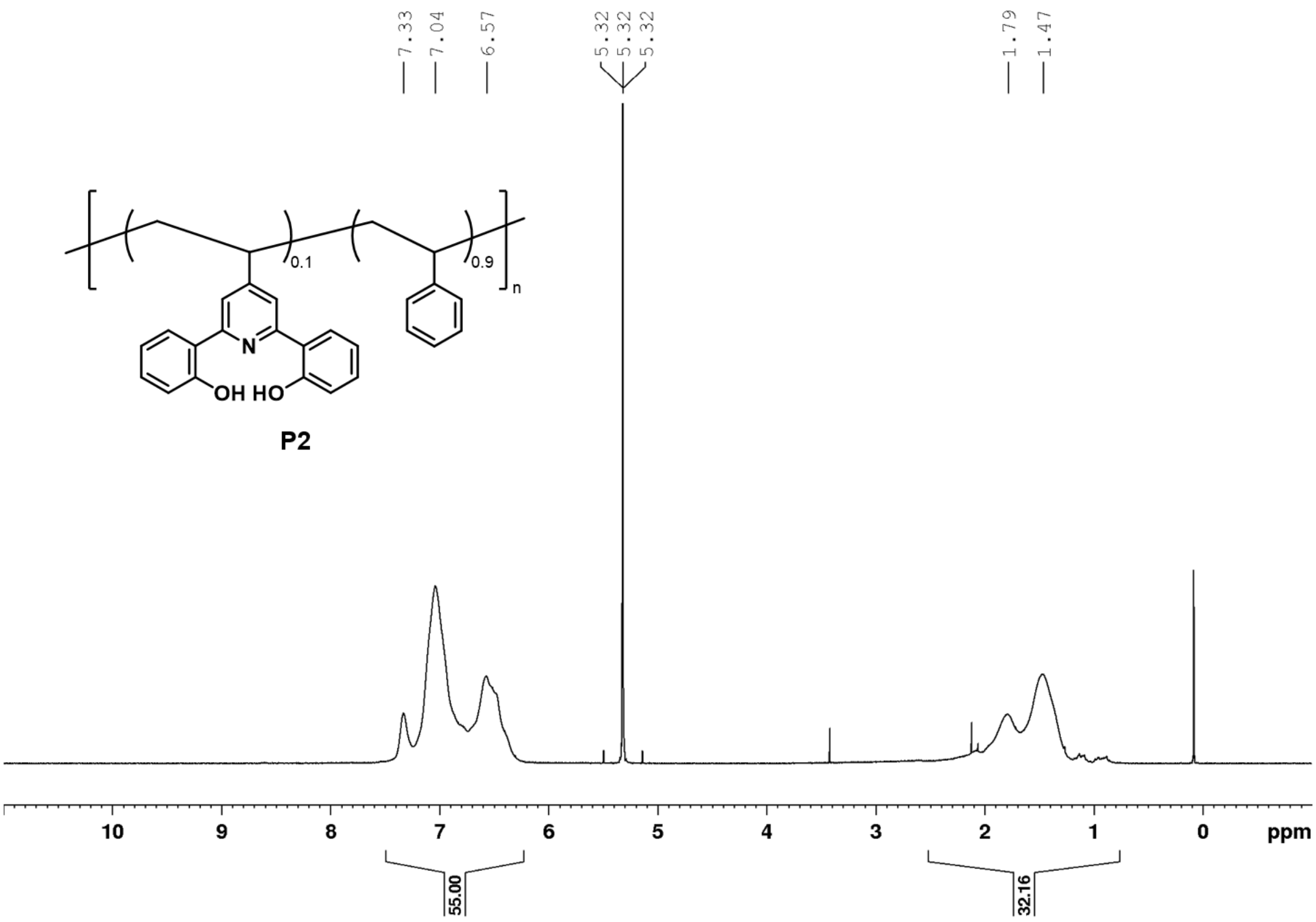

^{1}H NMR spectrum (500.13 MHz, CD_2Cl_2) of **P2**

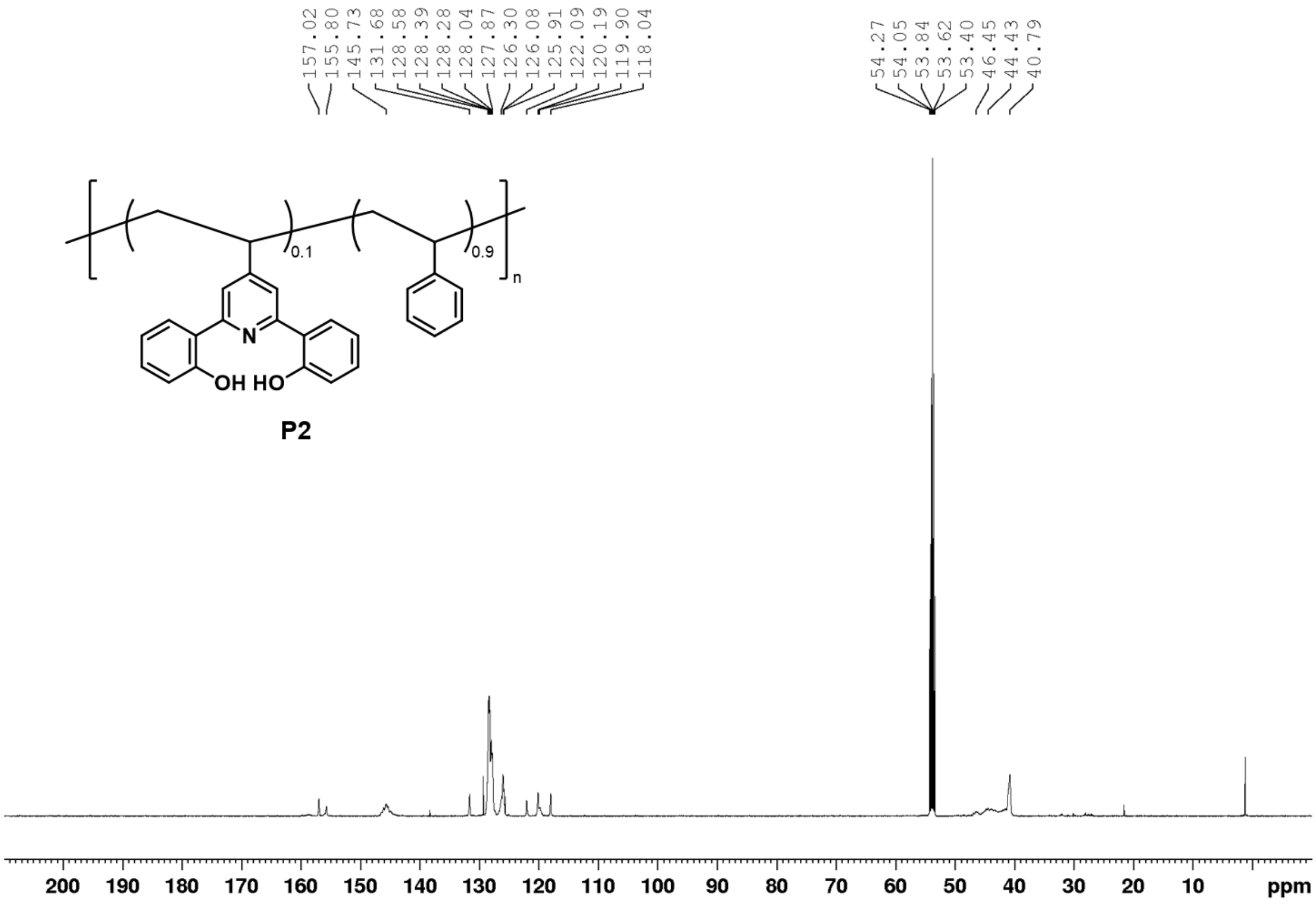

^{13}C NMR spectrum (125.76 MHz, CD_2Cl_2) of **P2**

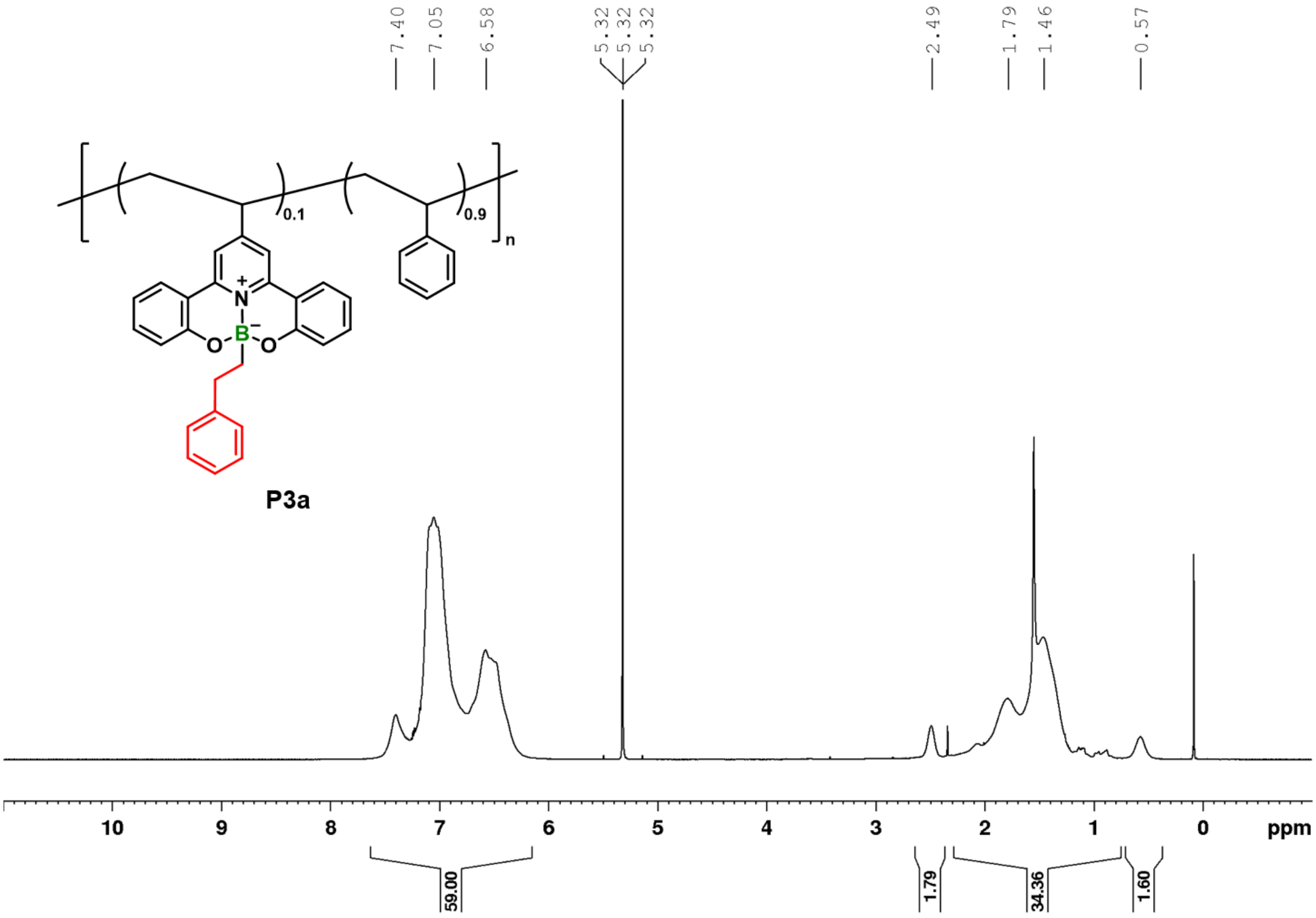

^{1}H NMR spectrum (500.13 MHz, CD_2Cl_2) of P3a

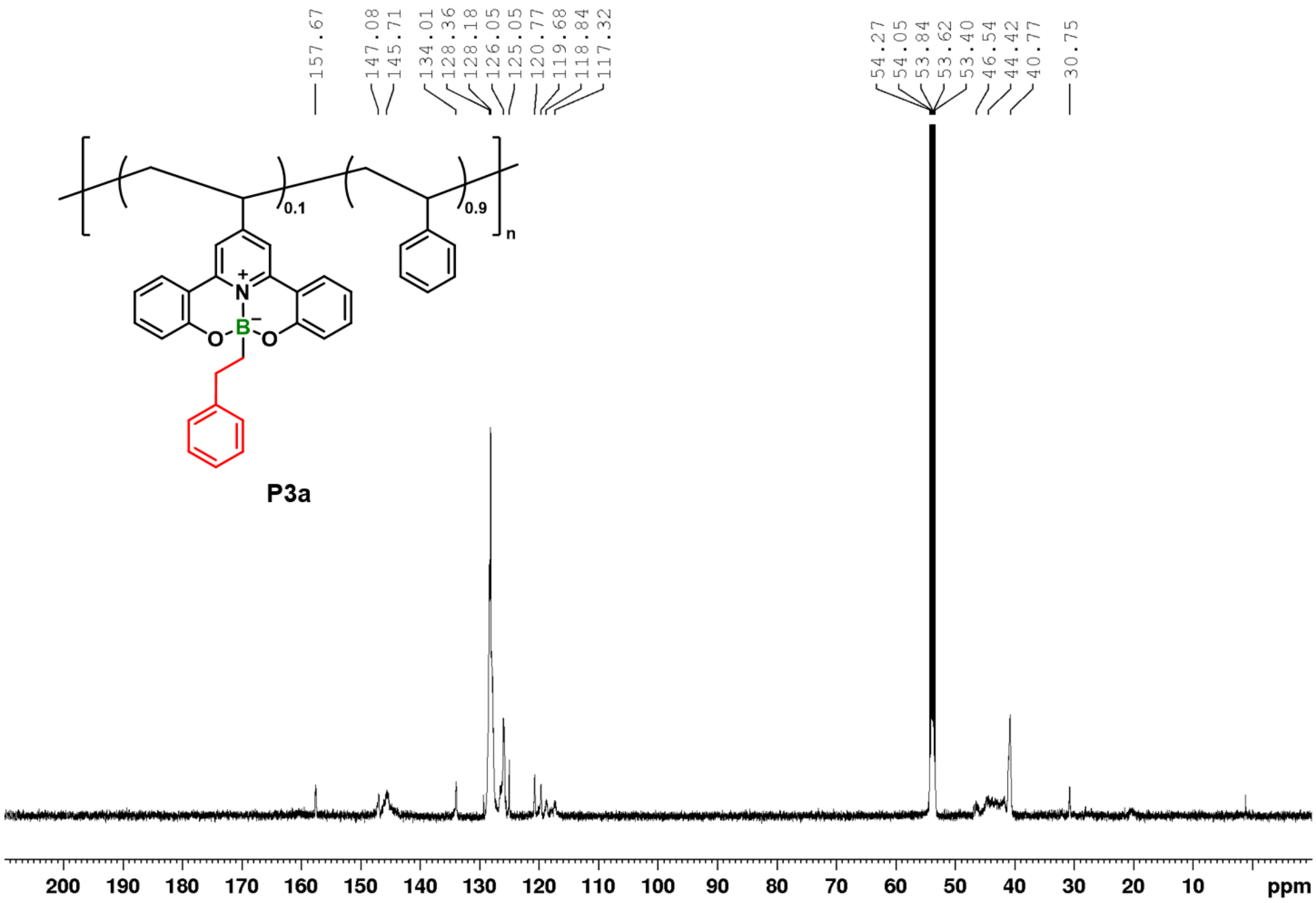

^{13}C NMR spectrum (125.76 MHz, CD_2Cl_2) of **P3a**

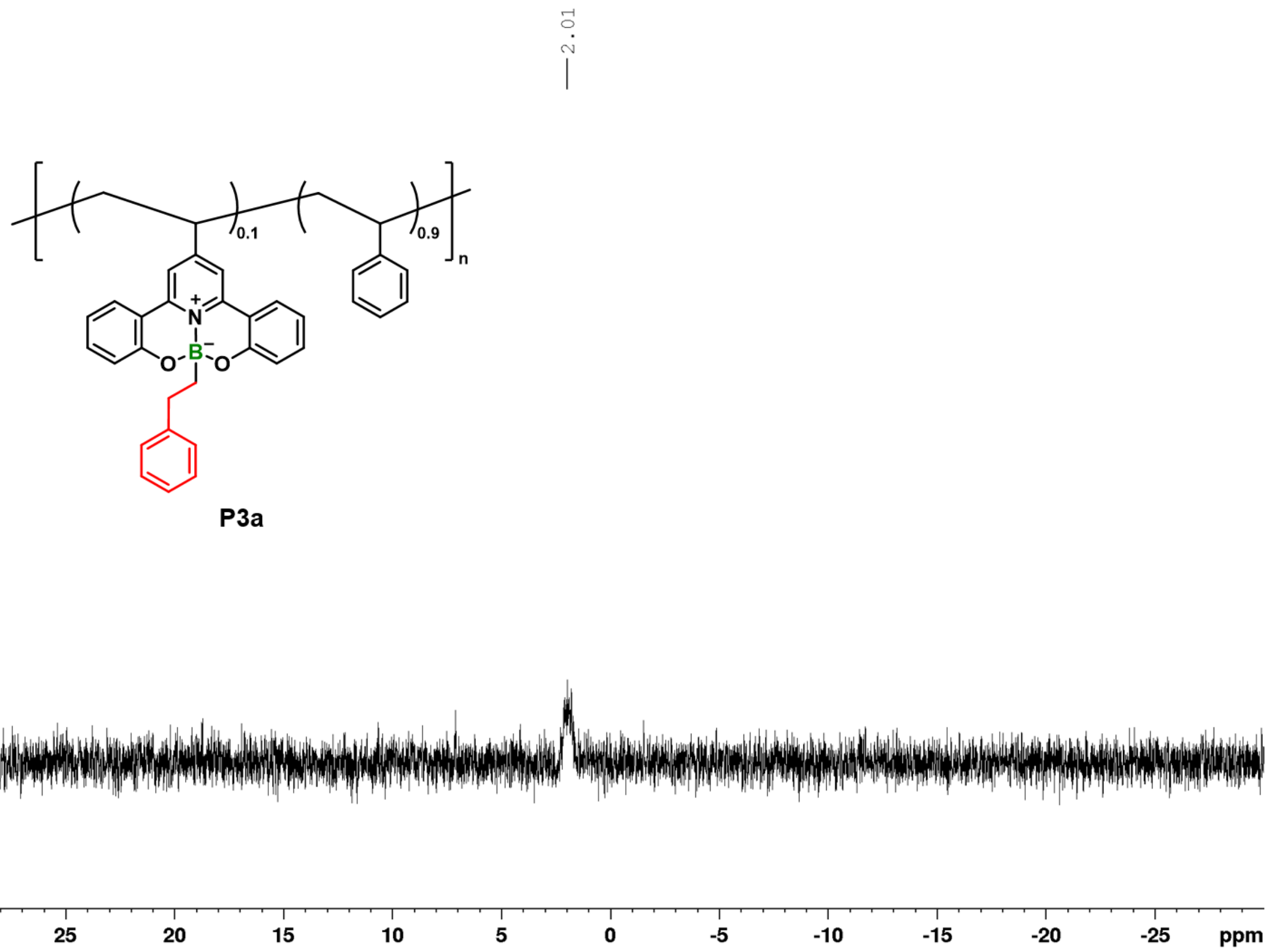

^{11}B NMR spectrum (160.46 MHz, CD_2Cl_2) of **P3a**

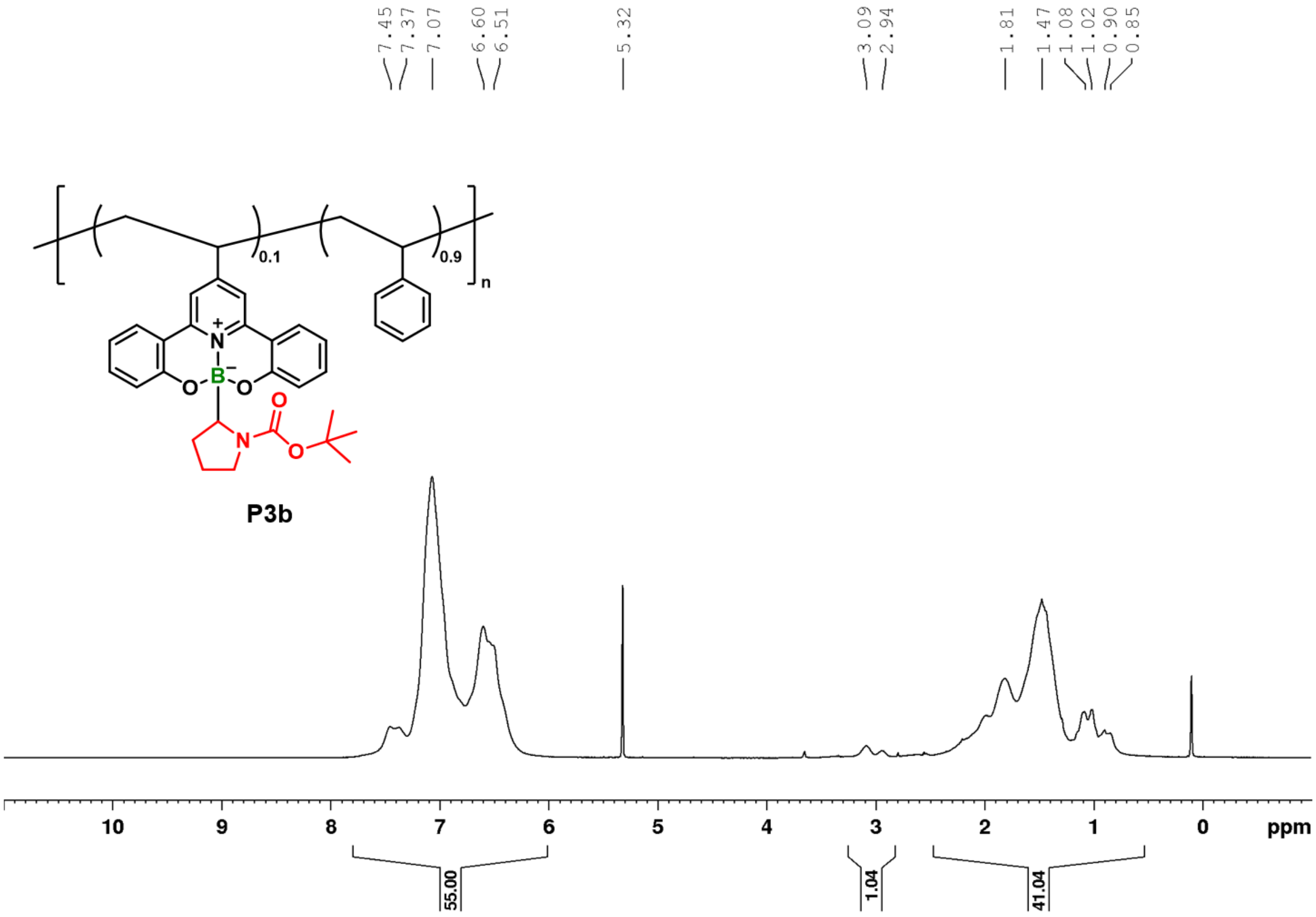

^{1}H NMR spectrum (500.13 MHz, CD_2Cl_2) of **P3b**

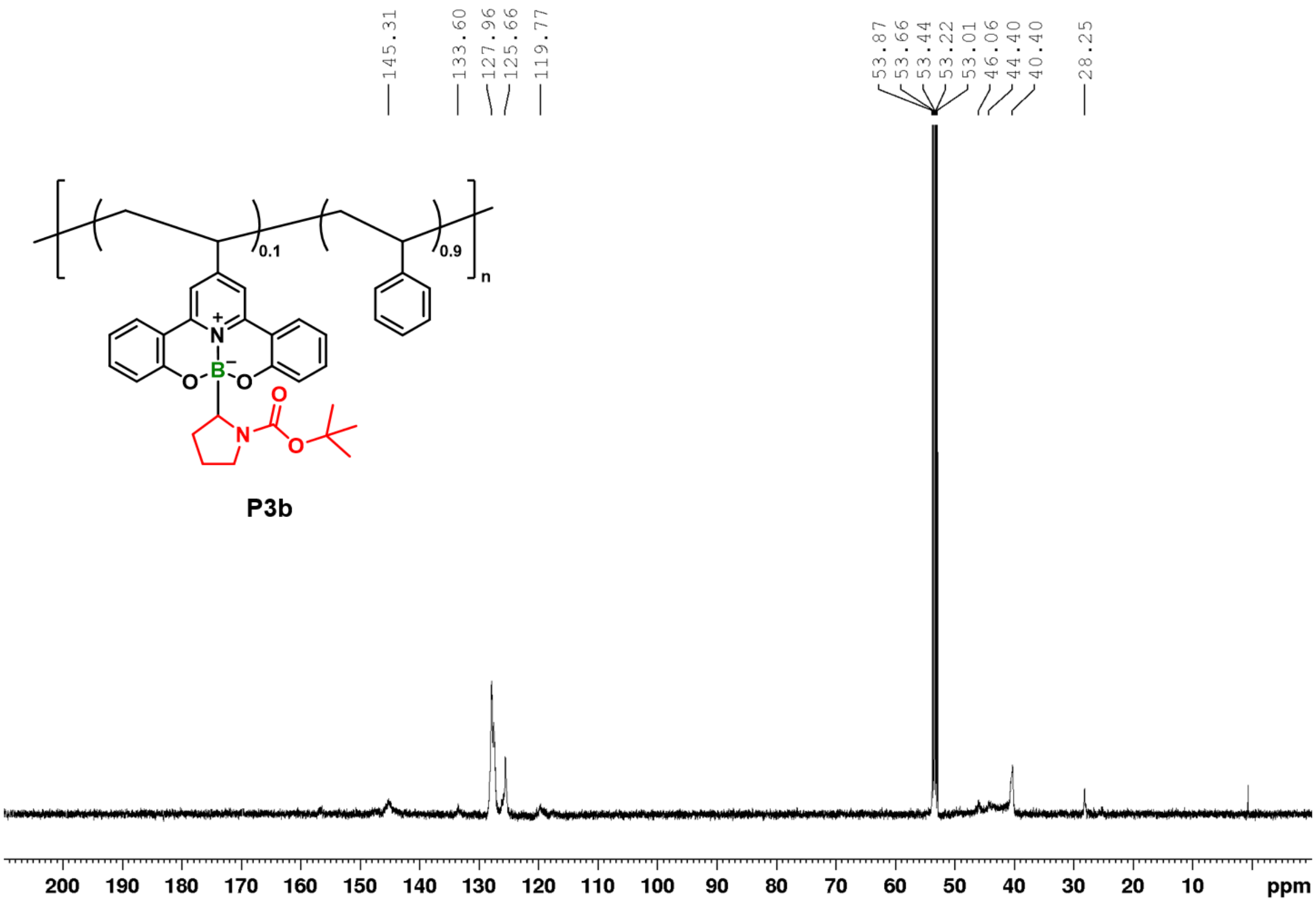

^{13}C NMR spectrum (125.76 MHz, CD_2Cl_2) of **P3b**

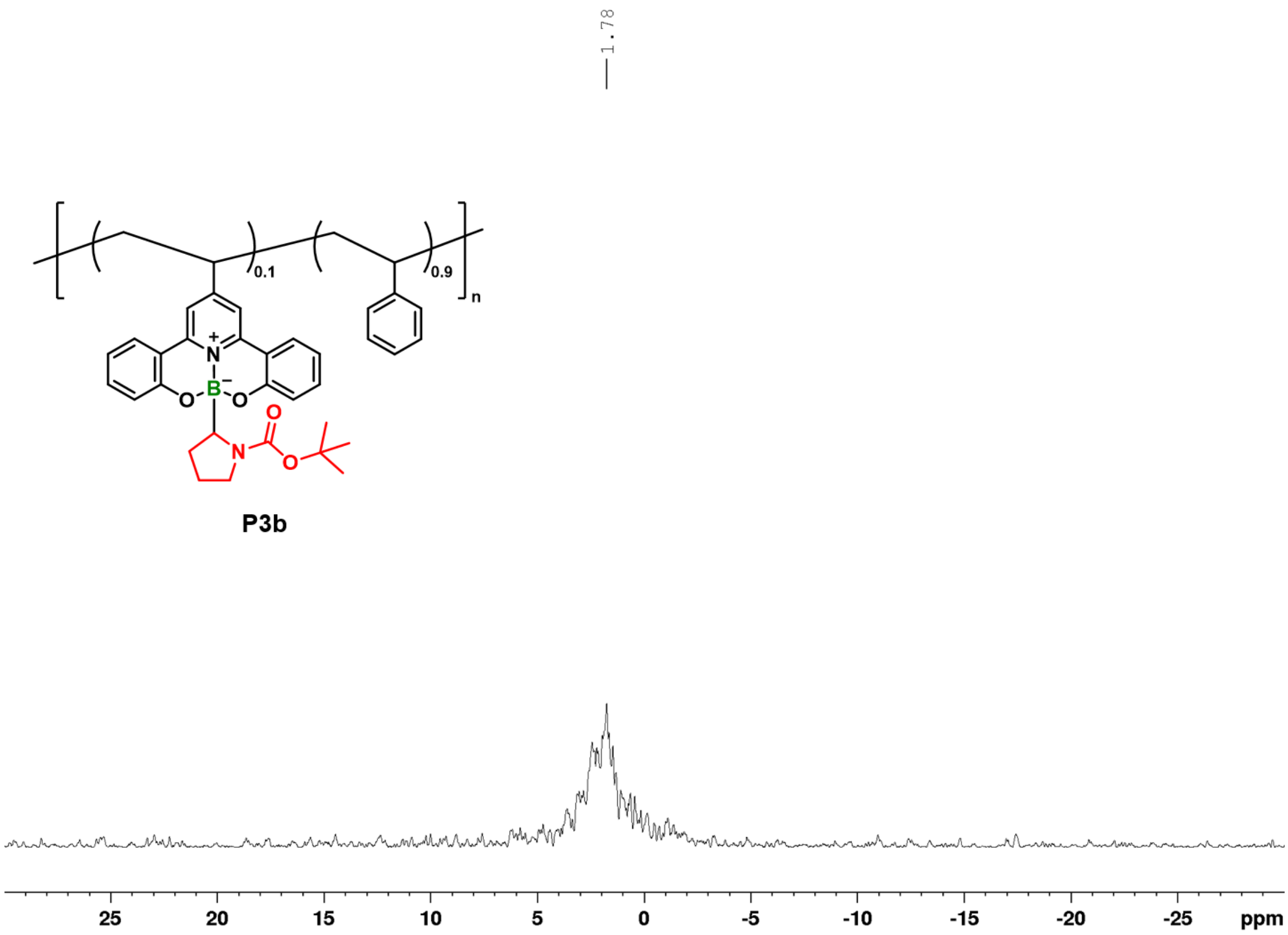

^{11}B NMR spectrum (128.38 MHz, CD_2Cl_2) of **P3b**

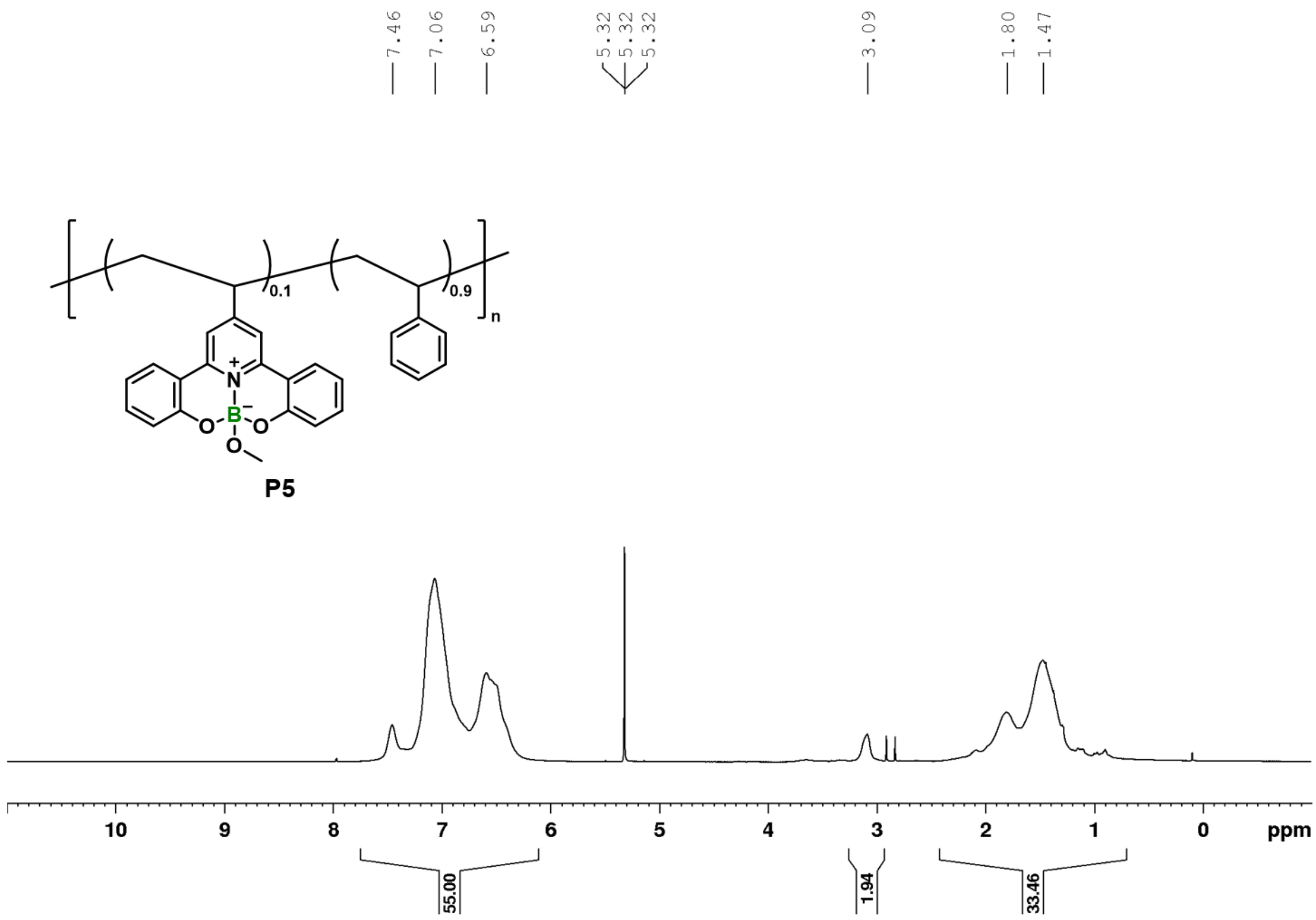

^1H NMR spectrum (500.13 MHz, CD_2Cl_2) of **P5**

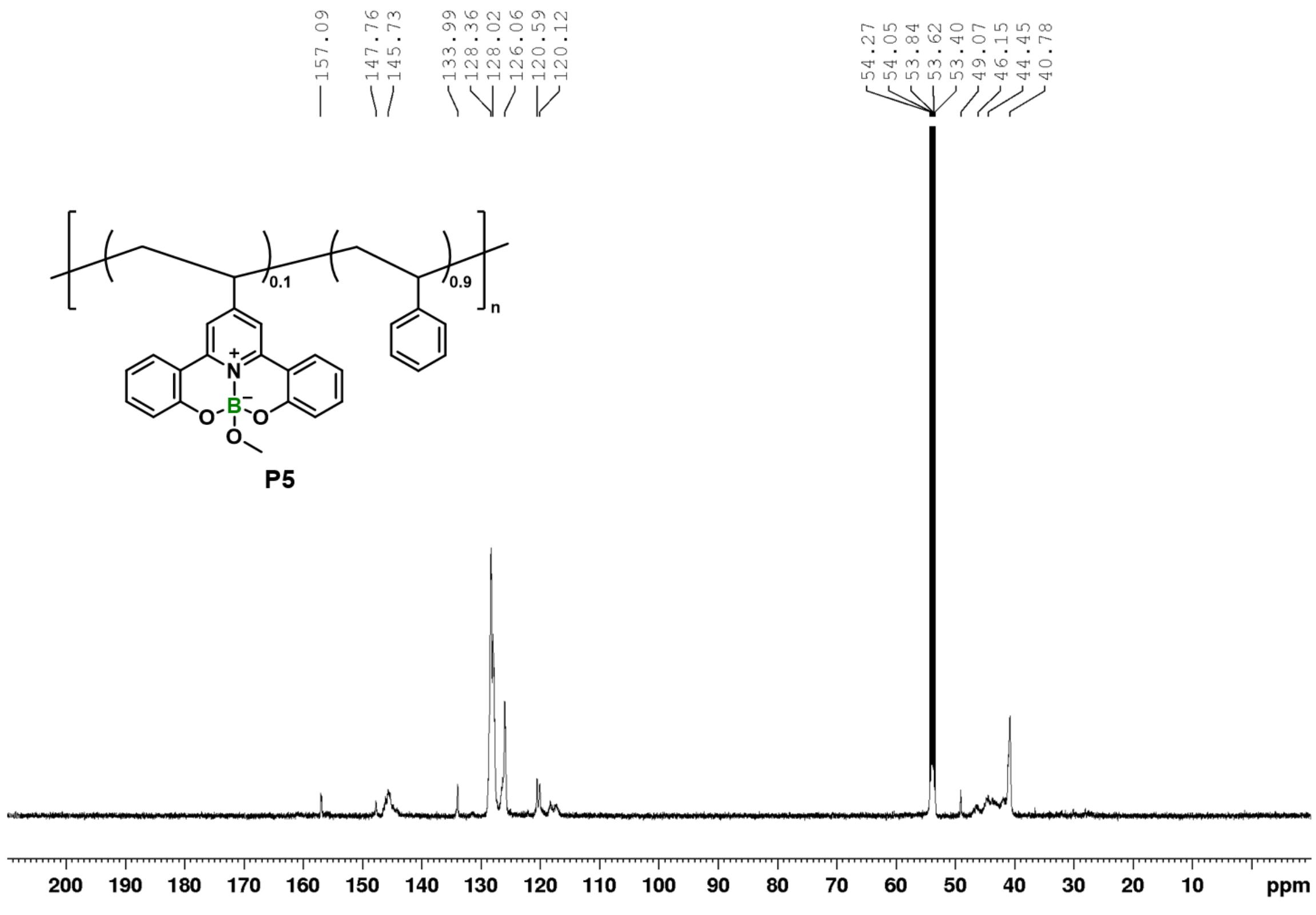

^{13}C NMR spectrum (125.76 MHz, CD_2Cl_2) of **P5**

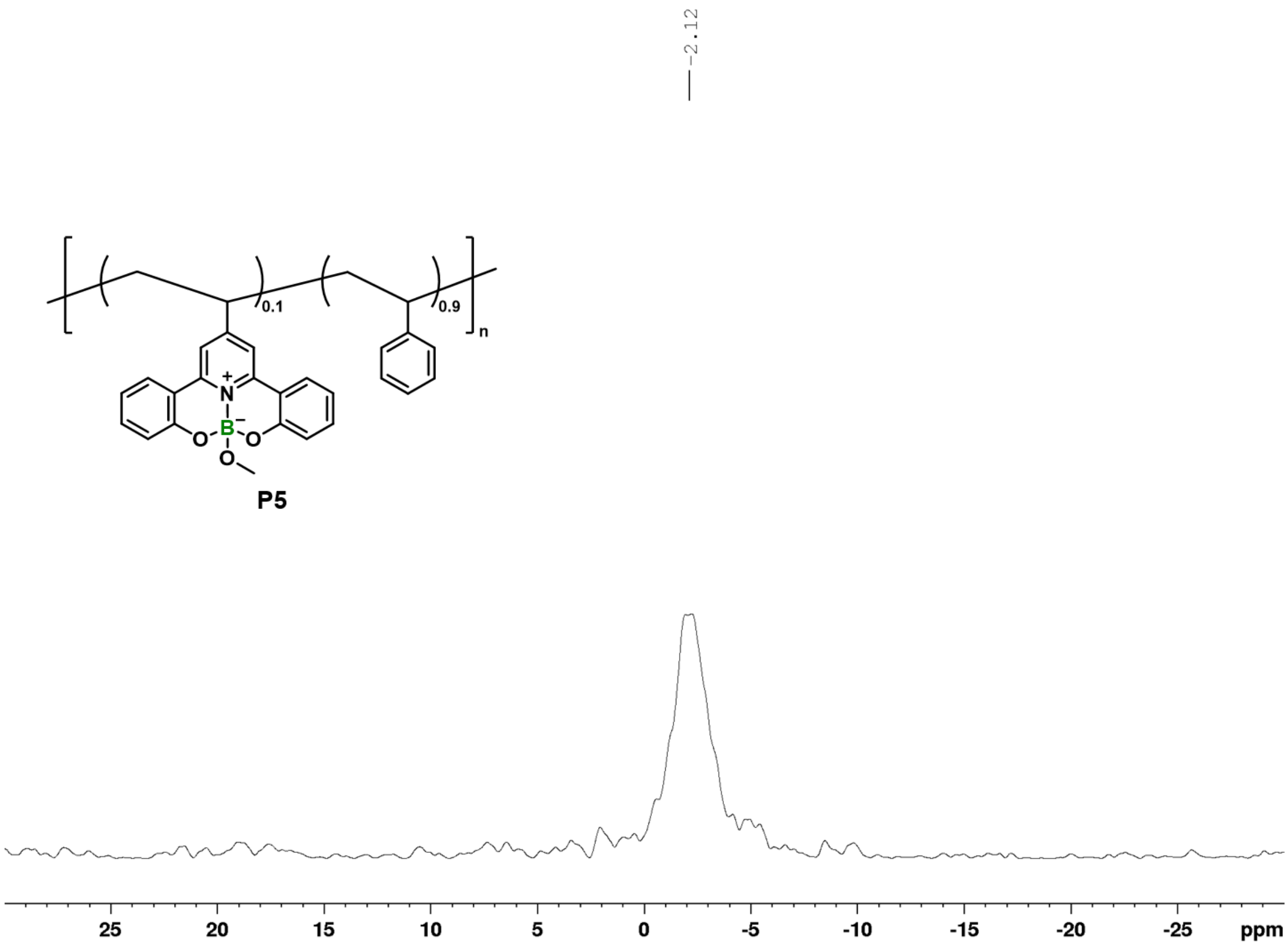

^{11}B NMR spectrum (128.38 MHz, $CDCl_3$) of **P5**

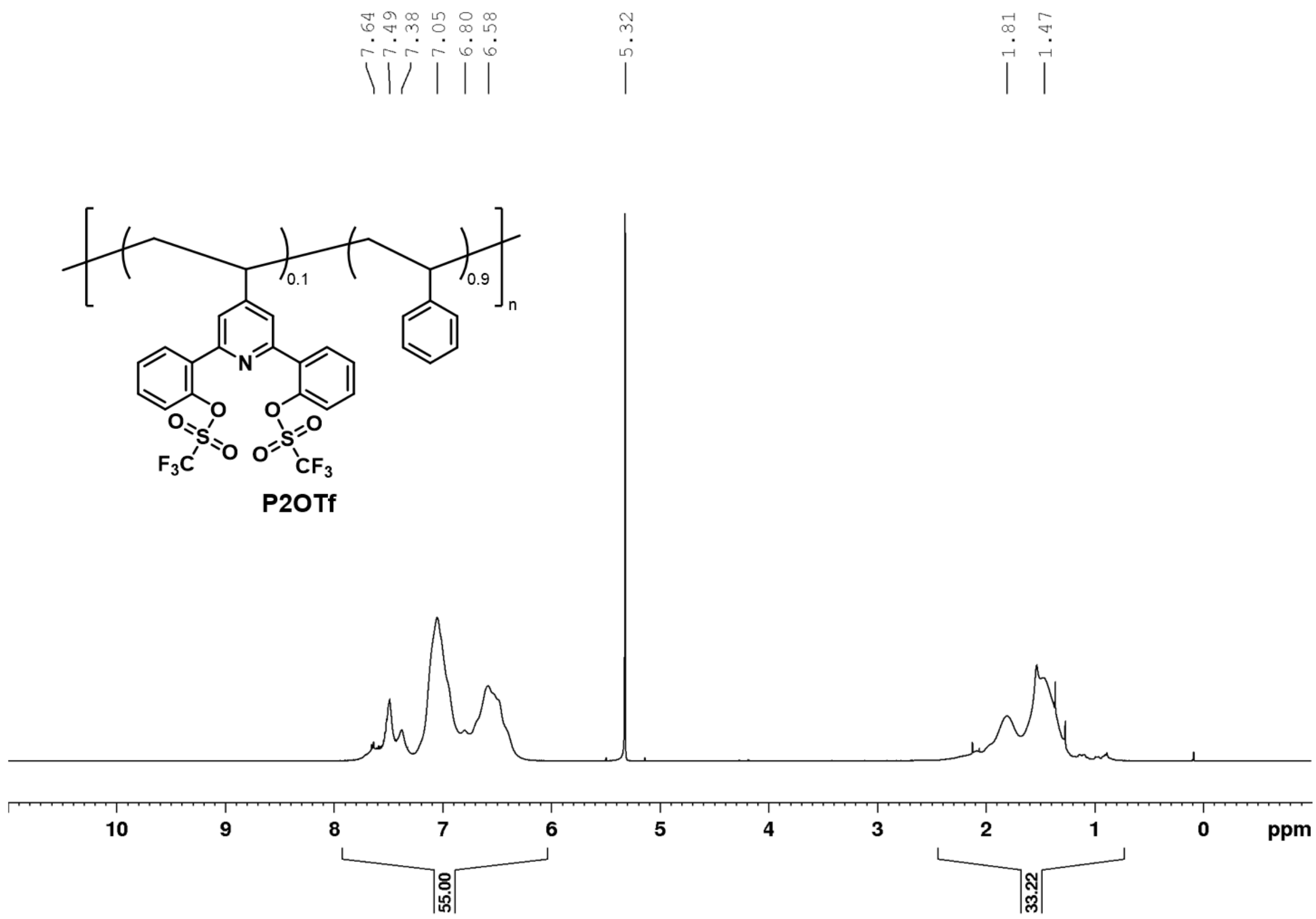

^{1}H NMR spectrum (500.13 MHz, CD_2Cl_2) of **P2OTf**

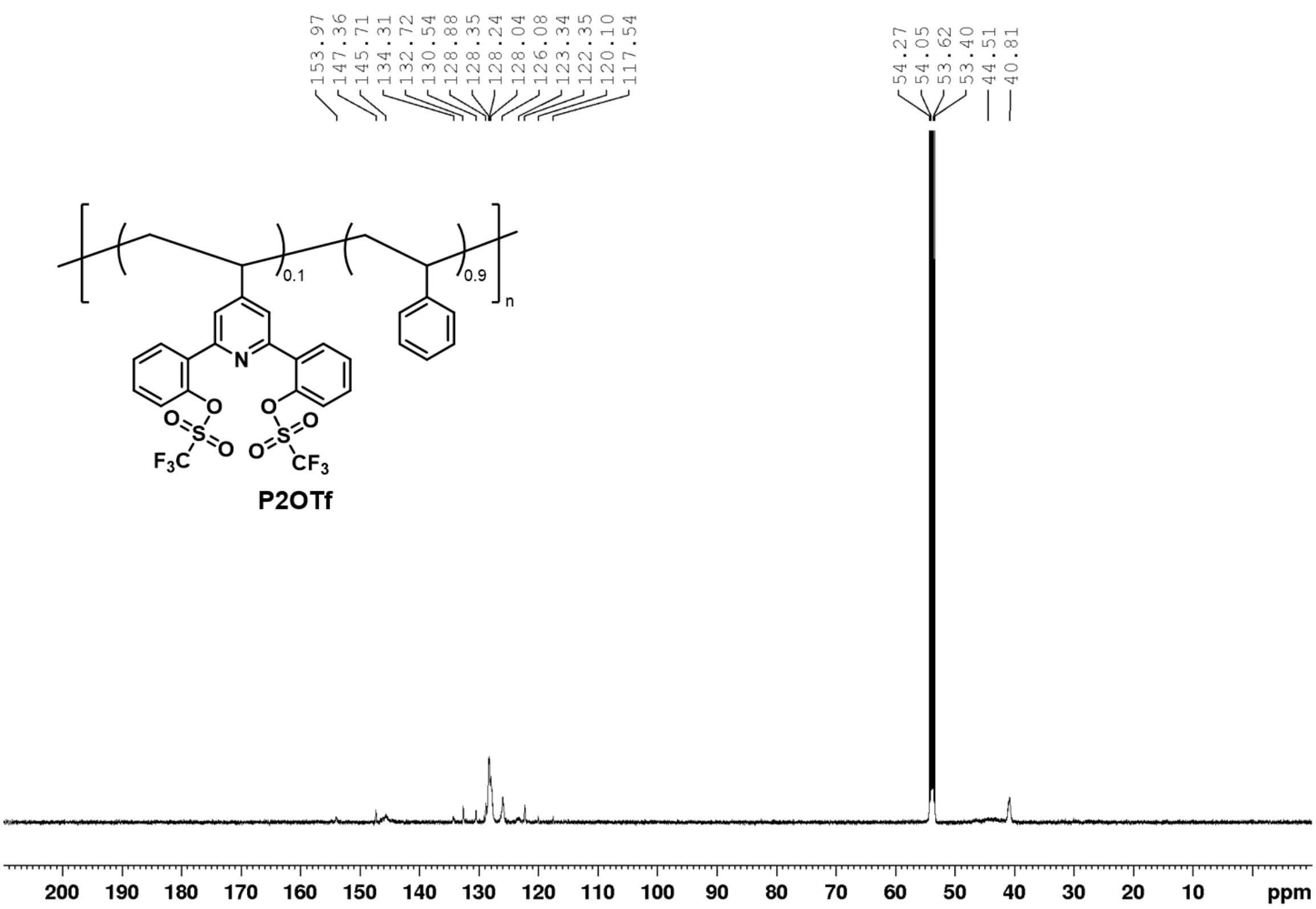

^{13}C NMR spectrum (125.76 MHz, CD_2Cl_2) of **P2OTf**

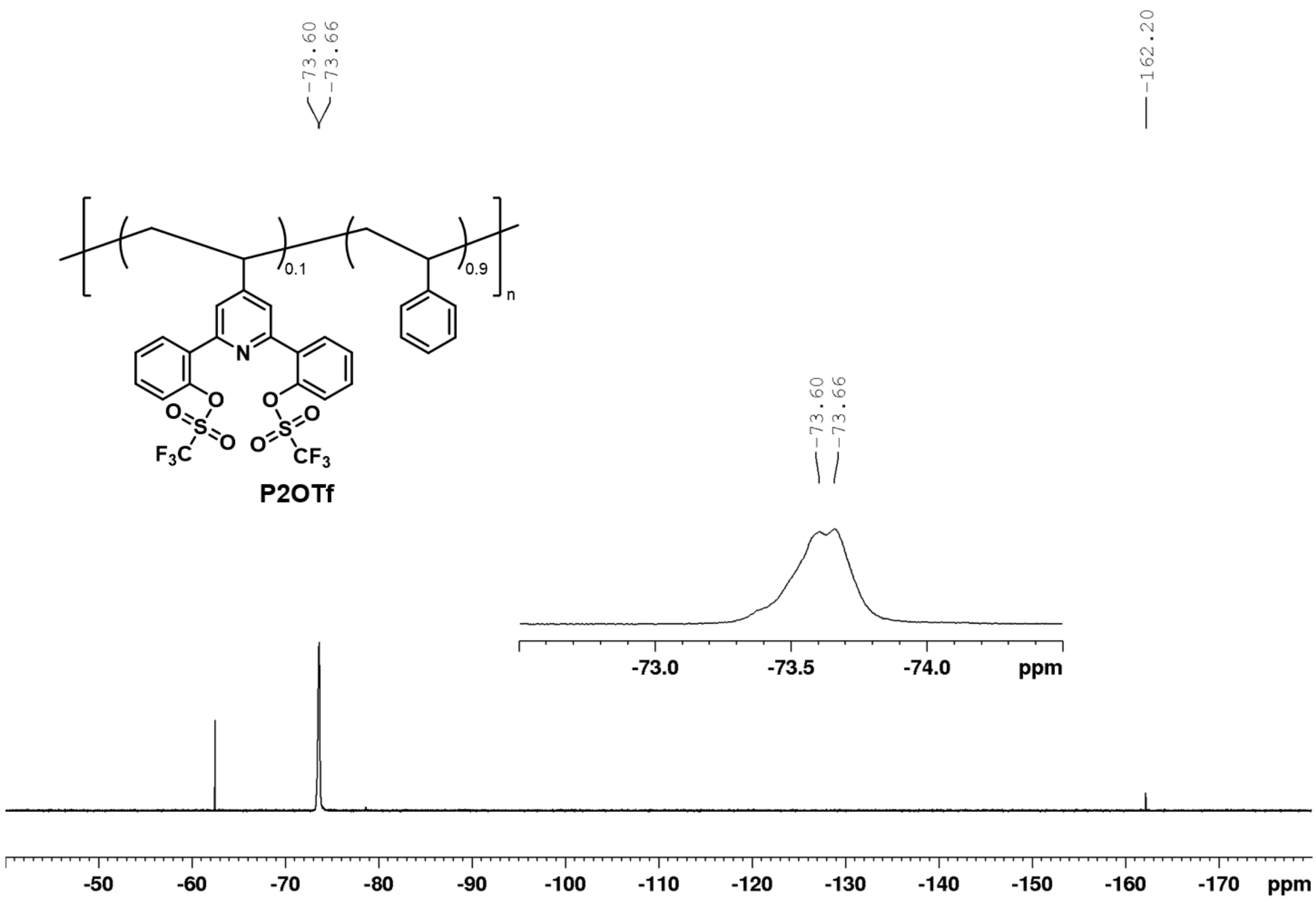

^{19}F NMR spectrum (470.59 MHz, CD_2Cl_2) of **P2OTf**

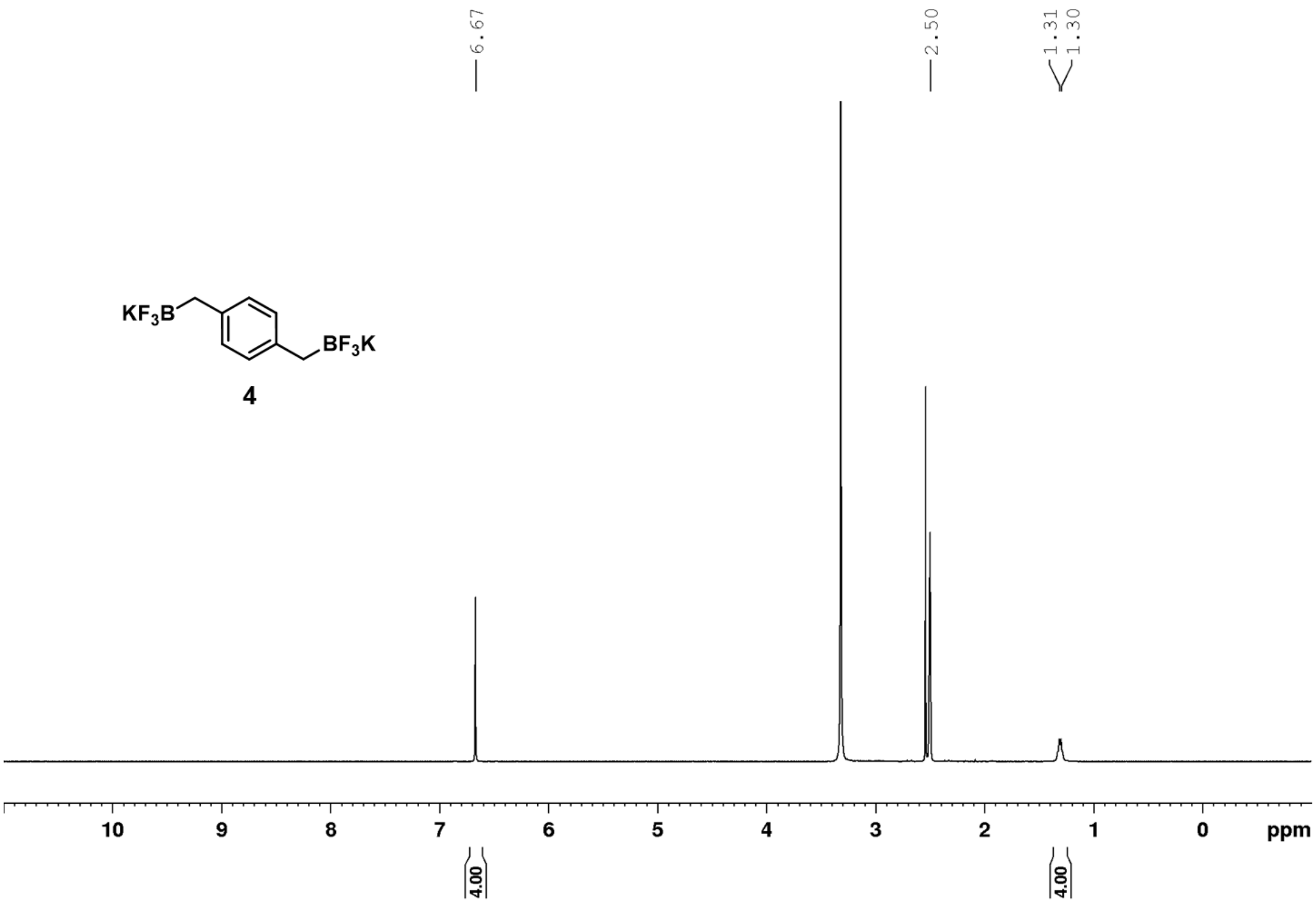

^{1}H NMR spectrum (500.13 MHz, DMSO-d_6) of **4**

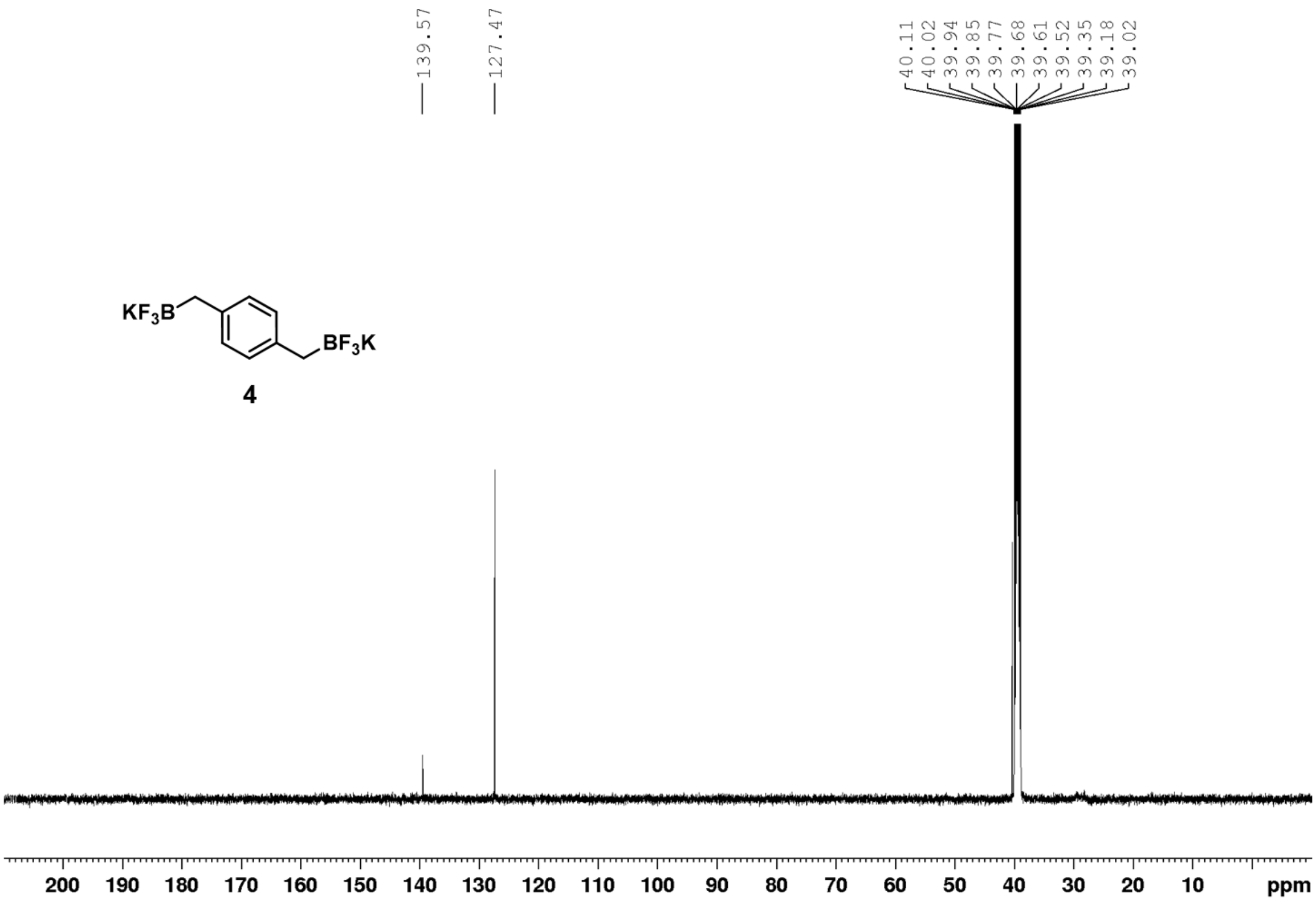

^{13}C NMR spectrum (125.76 MHz, DMSO-d_6) of **4**

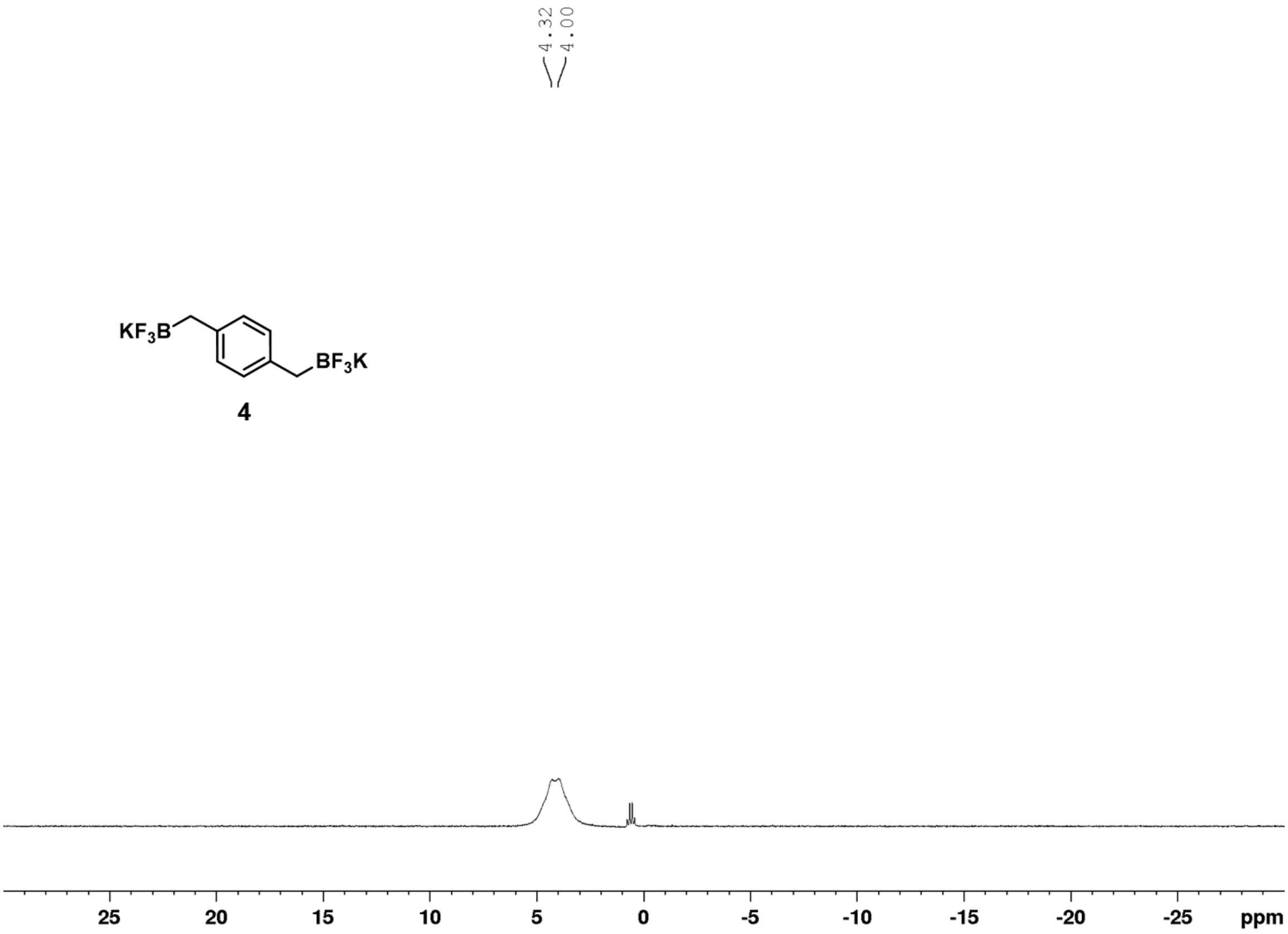

^{11}B NMR spectrum (160.46 MHz, DMSO-d_6) of **4**

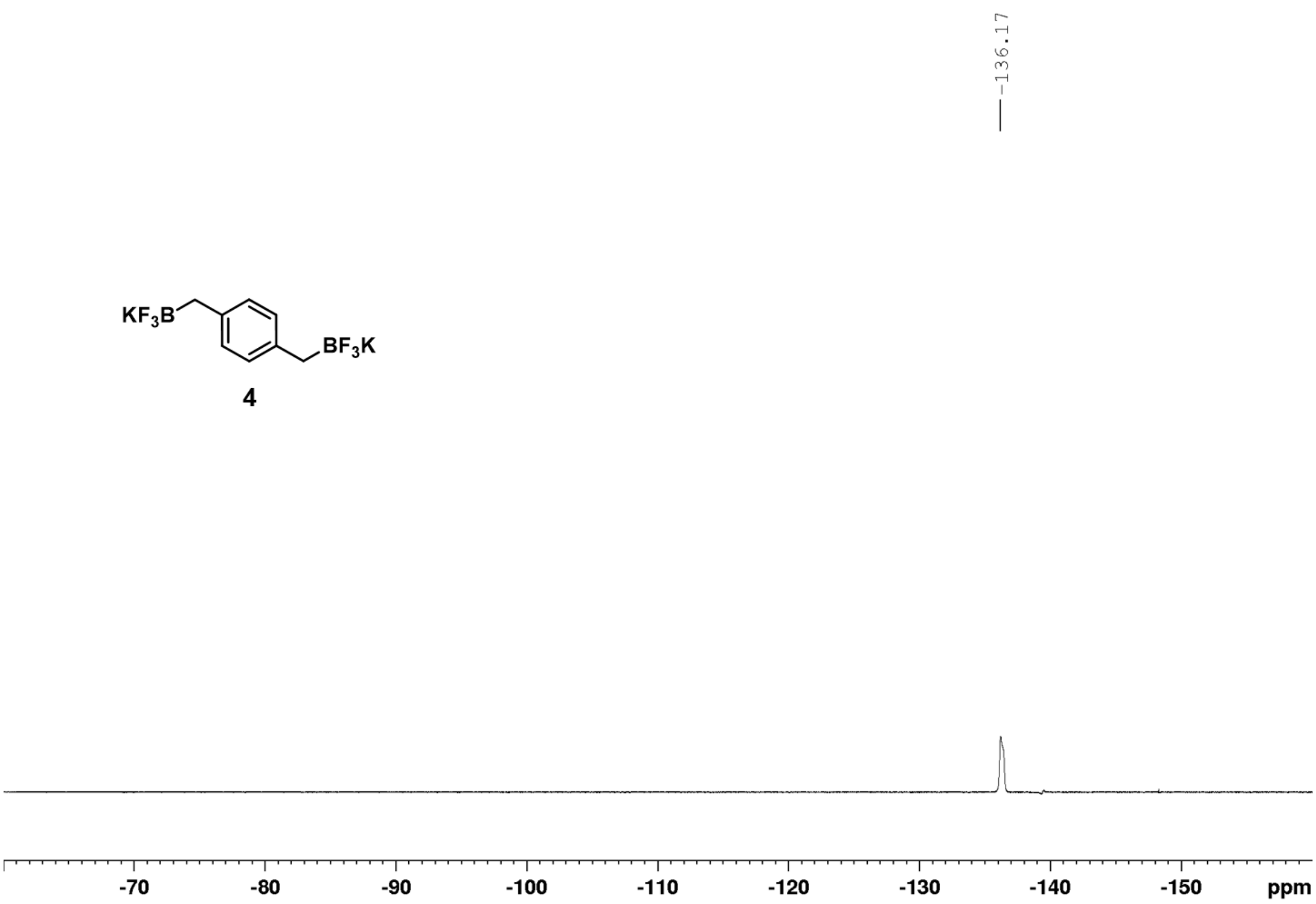

^{19}F NMR spectrum (470.59 MHz, DMSO-d_6) of **4**
